# Supplementary material for: Prostate cancer androgen receptor splice variant 7 biomarker study - a multicentre randomised feasibility trial of biomarker-guided personalised treatment in patients with advanced prostate cancer (the VARIANT trial) study protocol
Source: BMJ Open. 2019 Dec 18;9(12):e034708. doi: 10.1136/bmjopen-2019-034708 (PMC6937062; doi:10.1136/bmjopen-2019-034708)
Supplement: Supplementary data [file bmjopen-2019-034708supp001.pdf]

Supplementary Data – VARIANT Protocol

The constitution of the Trial Management Group (TMG): Chief Investigator(s), Sponsor representative, Co-Lead Investigator, Laboratory and Translational Science Lead, Statistician Lead, Trial Management Team, Co-Applicants and Collaborators who will attend TMG meetings as required.

The constitution of the Trial Oversight Committee (TOC) as a combined TOC whose members are independent of the trial: independent Chair, Independent Lab representative, Independent statistician and two independent patient representatives.

<u>Roles and Responsibilities</u>

Chief Investigator - Dr Rakesh Heer, Senior Lecturer and Consultant Urological Surgeon, Translational and Clinical Research Institute, Faculty of Medical Sciences, Newcastle University

Co-Lead Chief Investigator - Prof. John Staffurth, Professor in Oncology and Consultant Oncologist, Velindre Cancer Centre, Cardiff University

Laboratory and Translational Science Lead - Dr Emma Clark, Translational Research Associate, Translational and Clinical Research Institute, Faculty of Medical Sciences, Newcastle University

Principle Investigator - Prof Rob Jones, Professor of Clinical Cancer Research/ Honorary Consultant in Medical Oncology, University of Glasgow

Principle Investigator - Dr Ian Pedley, Clinical Director of NCCC and Clinical Oncologist, The Newcastle upon Tyne Hospitals NHS Foundation Trust

Co-applicants – [1] Dr Amit Bahl, Senior Lecturer, Consultant Oncologist and Clinical Director, University Hospitals Bristol NHS Foundation Trust; [2] Dr Simon Crabb, Associate Professor and Honorary Consultant in Medical Oncology, University of Southampton; [3] Dr Suneil Jain, Senior Lecturer and Consultant in Clinical Oncology, Queen's University Belfast.

PPI Representative - Dr John Marshall

Senior Statistician - Denise Howel, Population Health Sciences Institute, Faculty of Medical Sciences, Newcastle University

Statistician - Dr Holly Fisher, Population Health Sciences Institute, Faculty of Medical Sciences,, Newcastle University

Data Manager – Ruth Wood, Newcastle Clinical Trials Unit, Newcastle University

Senior Trial Manager – Jenn Walker, Newcastle Clinical Trials Unit, Newcastle University

Clark E, *et al. BMJ Open* 2019; 9:e034708. doi: 10.1136/bmjopen-2019-034708

Joint Trial Manager - Dr Miranda Morton and Shriya Sharma, Newcastle Clinical Trials Unit, Newcastle University

Sponsor - The Newcastle upon Tyne Hospitals NHS Foundation Trust

Funder - National Institute for Health Research – Research for Patient Benefit

Out of Hours Contact - Dr Rakesh Heer, Newcastle University

Trial Oversight Committee (TOC) Chair - Dr Alison Tree, Uro-oncology Trials Team Leader and Consultant Clinical Oncologist, The Institute of Cancer Research

Recruitment and screening

Patients will be approached during routine clinic appointments from urology or oncology clinical services. Potentially eligible patients will have the trial explained to them, provided with a Patient Information Sheet (PIS) and their medical notes reviewed to establish if they are likely to be eligible to take part in the trial. In addition to assessing patient eligibility against the inclusion and exclusion criteria, a complete medical history including the patient's age and detailed information about their prostate cancer history and metastases will be collected after consent.

Consent Procedure

Full written informed consent will be received by signing, dating and initialling the consent form, which will be witnessed by a member of the research team, who has documented and delegated responsibility, and will and check eligibility and counter-sign the consent form. The participant will specifically consent to; [1] their GP being contacted and informed of participation in the study; [2] access to relevant sections of their medical notes to carry out follow-up after the trial has ended; and [3] serial collection of blood samples for biomarker testing and storage in the Androgen Receptor Biology Bio-Bank (AR-3B) biobank for up to 10 years after the trial has ended.

Data collection methods

In addition to collecting standard care assessment of disease status data from intervention and control groups, trial specific questionnaire assessment (EORTC QLQ-C30 Quality of Life of Cancer Patients, EORTC QLQ-PR25 Quality of Life Prostate Cancer Module) and blood sample collection will take place at baseline, 12 and 24 week visits. Use of Health Services Questionnaire will be completed end of trial assessments. Participant data will only be identified using a unique individual participant identifier.

Standard care assessments:

The following clinical assessments will be conducted;

1. Cause of death (if appropriate)

2. Evidence of PSA progression (>25% and >2 ng/mL above the nadir and confirmed by a second value >3 weeks later)
3. Clinical evidence of progression, stage and type of progression (biochemical, radiological or symptomatic)
4. Result of routinely collected PSA and testosterone measurements, full blood count and biochemistry tests
5. Details of anti-cancer therapy, including dates of treatments for ongoing anti-cancer therapy.

Questionnaires:

The EORTC quality of life questionnaire is an integrated system for assessing the health-related quality of life (QOL) of cancer patients participating in clinical trials. There is a set of core questions (QLQ-C30), supplemented by a prostate cancer specific module (PR25). PR25 is a diagnosis-specific module designed to be used in conjunction with the QLQ-C30, it is intended for use among a wide range of patients with prostate cancer varying in disease stage and treatment modality.

The QLQ-C30 consists of thirty questions incorporating; [1] functional scales, symptom scales and a number of items assessing additional symptoms commonly reported by patients with cancer, assessed on a four point scale and [2] a global health status/quality of life scale, assessed on a seven point scale. The PR25 module consists of 25 questions incorporating functional and symptom scales, all assessed on four point scale. The Use of Health Services questionnaire consists of ten questions assessing participant's use of health services over the course of their participation in the trial.

Data Management and Archiving

Data including the number of patients screened, approached and interested in taking part will be collected via a screening log. Trial and screening data is collected on electronic case report forms (eCRFs) using password limited access, secure web-based interface for data entry with inbuilt back-up facility, will be managed using a Clinical Data Management System (Elsevier's MACRO™) overseen by the Newcastle Clinical Trials Unit (NCTU). Individual access will be limited according to delegated roles and duties. Data will be handled, computerised and stored in accordance with the UK Data Protection Act 2018. All trial data will be retained in accordance with the latest Directive on GCP (2005/28/EC).

Participant clinical information will not be released without the written permission of the participant, except as necessary for monitoring and auditing by the Sponsor, its designee, Regulatory Authorities, the Trial oversight committee (TOC) or the Research Ethics Committee (REC). Trial data will be released to the trial statistician for analysis, to Paul O'Gorman Newcastle University biobank researchers after trial analysis and used in planning any future, definitive trial. All trial data will be stored for 10 years, in accordance with GCP and sponsor approved SOPs.

Clark E, *et al. BMJ Open* 2019; 9:e034708. doi: 10.1136/bmjopen-2019-034708

Supplementary Data – VARIANT Protocol

AR-V7 biomarker driven personalised treatment pipeline

Biomarker driven personalised treatment will be based on current scientific evidence from the Adnatest assay biomarker founding lab (John Hopkins, Baltimore, USA). A biomarker positive result is when a read is detected at 35 qPCR cycles or less and the recommendation will be to proceed with chemotherapy. A biomarker negative result is when a read is detected over 35 qPCR cycles (or there is no read at any qPCR cycle), and the recommendation will be to proceed with hormonal therapy (Enzalutamide or Abiraterone).

Participants will be required to give 30ml of blood at baseline (0 weeks), 12 weeks and 24 weeks. Samples will be routinely processed for biomarker read-out and AR-3B biobank storage at the Newcastle University central analysis lab as detailed in the VARIANT biomarker flow chart below. Following biomarker data analysis and data verification (according to sponsor agreed analytical and validation plan and SOPs), for participants randomised to the intervention standard treatment arm (AR-V7 biomarker guided), the baseline biomarker result and biomarker treatment recommendation will be sent securely within 10 working days to the local PI and delegated research staff. The PI and local trial team will then communicate with the participant regarding the biomarker guided treatment. Any modifications to the recommended treatment for participants will be made at the discretion of the treating clinician, based on their clinical judgement. Biomarker results from blood samples taken at weeks 12 and 24 will not be made available to the PI, local trial team or participant.

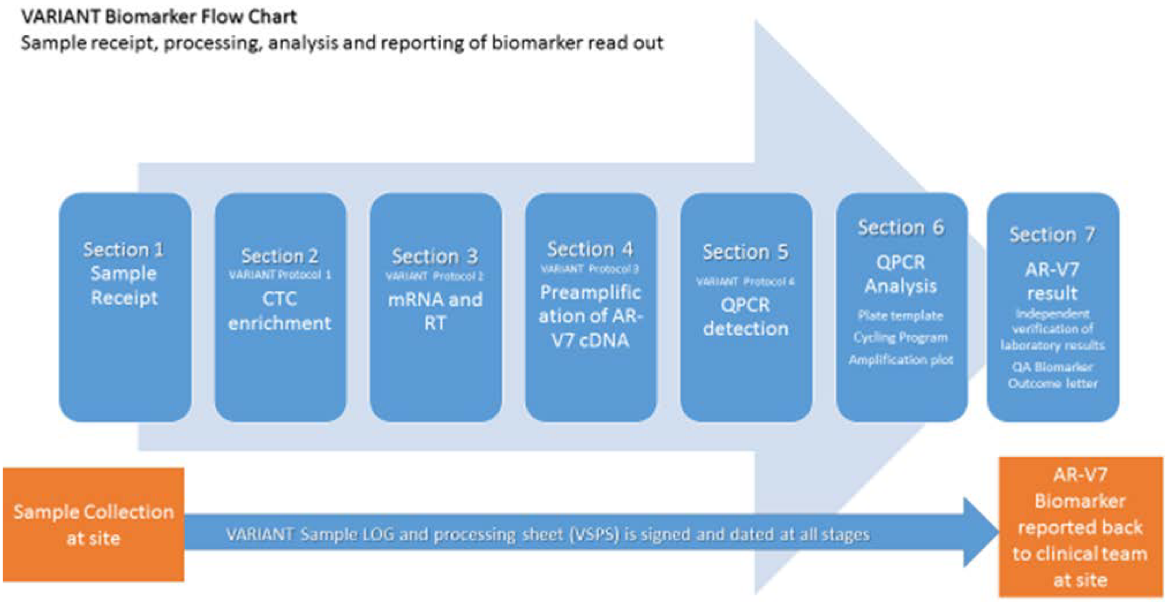

For participants randomised to the standard treatment arm (not AR-V7 biomarker guided), biomarker results will not be made available to the PI, local trial team or participant for any of the blood samples analysed (baseline, week 12 and week 24).

AR-V7 biomarker cross-site validation

An additional twenty participants selected at randomisation will provide a further (10ml) blood sample for cross site validation analysis of the biomarker at the Cardiff Central Analysis Lab using the commercially available AdnaTest ProstateCancerPanel AR-V7 circulating tumour cell (CTC) quantitative RT-PCR (RT-qPCR) assay (Qiagen®).

Androgen Receptor Biology driving hormone resistance

Research suggests that key 'sub-populations' or clones of molecular alterations (of which AR-V7 is an important subtype), compete with each other and are drivers of treatment resistance. In addition to performing AR-V7 biomarker assay, a fuller capture of AR-related CRPC biology will be achieved by collecting CTCs, plasma, buffy coat and red blood cell derivatives to compile an Androgen Receptor Biology Bio-Bank (AR-3B). Whole and CTC depleted blood sample derivatives will be collected and biobanked at 12 weekly intervals from baseline (prior to treatment) throughout follow up (to a maximum of 24 weeks +/- 2 weeks), including all trial captured data.

It is also important to acknowledge the expression of other AR splice variants are also associated with resistance to hormonal treatment(1,2), as are other AR pathway alterations such as AR mutations and amplifications(3). Ongoing discussions in the field as to the discriminatory value of solely detecting AR-V7 expression, may ultimately lead to a combination test that will improve treatment stratification and patient outcomes(4). However, at this time, AR-V7 remains the forerunner and a formal understanding of biomarker characteristics in the advanced prostate cancer treatment setting is required 'to best inform' a formal large-scale testing.

The AR-3B biorepository resource will be used to assess total VARIANT expression (capturing all VARIANTs in a single qPCR reaction) and explore (but not be limited to) AR hot spot mutations/sequencing/amplification and other mutations such as PTEN/p53/MYC gain/RB1 loss/MET gain/PARPi and further molecular pathways based on yet to be defined but new emerging data in time, in blood and blood derivatives.

This resource will provide added value to the feasibility study, by banking processed biomarker tissue for additional biomarker measures that may also contribute to hormone targeting resistance (or the emergence of biomarkers for chemo-sensitivity), and importantly be relevant in prostate cancer management. Blood samples will be transported, stored, accessed and processed in accordance with sponsor approved SOPs following appropriate legislation relating to the use and storage of human tissue for research purposes and such activities shall at least meet the requirements as set out in the 2004 Human Tissue Act and 2006 Human Tissue (Scotland) Act.

The results of any other research utilising blood samples (including those in the control arm), will not be reported back to the clinical team or to participants, this data is for research purposes only and will be published in appropriate peer reviewed scientific journals. Participants will remain anonymised in all publications.

Supplementary Data – VARIANT Protocol

Androgen Receptor Biology Bio-Bank (AR-3B) sample storage governance

Samples will be appropriately labelled in accordance with trial protocol as described in the current VARIANT trial site blood collection manual (Version 1.0 28th March 2019) to comply with the General Data Protection Regulation (GDPR) and Data Protection Act (GPA) 2018 and pseudo-anonymised with linkage to participant details possible only through the Participant Identification Log assessable only to delegated personnel. Sample shipment will be tracked and sample receipt at central analysis labs recorded using a specific sample collection MACRO™ database which is separate to the main trial database.

All AR-3B biobank samples are stored under HTA license 12534, under the Designated Individual, Dr. Christopher Morris (NICR). All samples are held under the custodianship of Mr. Rakesh Heer (Chief Investigator), who is responsible for the curation of these samples and ensuring compliance with the Human Tissue Act (2004) and GCP. Samples will be tracked and stored using Achiever medical LIMS.

Blood samples sent to the Cardiff Central Analysis Lab, will be consumed within 7 days of receipt and are not under the remit of the HTA. All RNA and DNA derivatives will be transferred to the Newcastle University AR-3B biorepository for storage at the end of the trial.

<u>Supplementary Section References:</u>

1. Van Etten JL, Nyguist M, Li Y, Yang R, Ho Y, Johnson R, et al. Targeting a Single Alternative Polyadenylation Site Coordinately Blocks Expression of Androgen Receptor mRNA Splice Variants in Prostate Cancer. Cancer Res. 2017;77(19):5228-35

2. Henzler C, Li Y, Yang R, McBride T, Ho Y, Sprenger C, et al. Truncation and constitutive activation of the androgen receptor by diverse genomic rearrangements in prostate cancer. Nat Commun. 2016;7:13668

3. De Laere B, van Dam PJ, Whitington T, Mayrhofer M, Diaz EH, Van den Eynden G, et al. Comprehensive Profiling of the Androgen Receptor in Liquid Biopsies from Castration-resistant Prostate Cancer Reveals Novel Intra-AR Structural Variation and Splice Variant Expression Patterns. Eur Urol. 2017;72(2):192-200

4. Zhang J, Cunnigham JJ, Brown JS and Gatenby RA. Integrating evolutionary dynamics into treatment of metastatic castrate-resistant prostate cancer. Nat Commun. 2017;8(1):1861

Clark E, *et al. BMJ Open* 2019; 9:e034708. doi: 10.1136/bmjopen-2019-034708

To be printed on local headed paper

PATIENT INFORMATION SHEET

The Variant 7 Biomarker Feasibility Study

The **VARIANT** Study

We are inviting you to take part in a research study

Please read the following information to help you decide if you want to take part. It will explain why we are doing this research and what it might mean for you. You are free to decide whether or not to take part in this study. You do not have to decide straight away and you can talk to your friends/family about it. Ask us if you have any questions or you want to know more. If you choose not to take part, this will not affect the care you get from your doctors.

Study Summary

- In this study we will be taking 3 blood samples and asking patients to complete some questionnaires during their usual hospital appointments.
- Patients with **advanced metastatic prostate cancer** (cancer that has spread from the prostate to other parts of the body), often eventually stop responding to initial hormone therapy. After this point, treatment is usually with either:
    1) **Further hormonal treatment called next generation hormone treatment OR**
    2) **Non-hormonal treatment such as chemotherapy or radiotherapy**
- There is currently no clear guidance for which of these two different standard care treatment options a patient should receive.
- Testing the level of AR-V7 (a type of protein) in blood could suggest which of these two treatments patients will respond to best. The AR-V7 test is known as a biomarker test.
- VARIANT is looking to find out if the AR-V7 biomarker test is helpful to support doctors and patients in choosing between these treatment options.
- In this study, half of patients will receive treatment guided by the AR-V7 biomarker test. The other half will receive treatment as usual.

Please read the following information to see if you may be interested in taking part.

Clark E, *et al. BMJ Open* 2019; 9:e034708. doi: 10.1136/bmjopen-2019-034708

What is a biomarker test?

Biomarkers are substances that can be found and measured in parts of the body, in this case, the blood. Biomarker testing is a type of test that looks for these substances to give doctors information about a patient's health. **The AR-V7 blood test is a biomarker test looking for the AR-V7 protein.**

Why is VARIANT needed?

Initial treatments for advanced metastatic prostate cancer include hormone treatment alone or chemotherapy in combination with hormone treatment. Eventually most patients with advanced prostate cancer stop responding to initial hormone treatment. At this point, patients usually receive treatment with either further hormone treatment, known as next generation hormone treatment (such as abiraterone or enzalutamide), or with non-hormonal options (typically chemotherapy, or in some cases radium-223 radiotherapy). Although there are fewer side effects associated with next generation hormone treatment relative to chemotherapy, only 30-50% of patients will respond well to further hormone treatment.

The **AR-V7 biomarker is found in the blood** of some men who have received initial hormone treatment for prostate cancer. Recent studies have suggested that patients who have this biomarker in their blood may be less likely to respond well to advanced hormone therapy. Measuring the amount of this biomarker in blood (which is not usually tested for), may be useful to help guide choice of treatment for patients with advanced metastatic prostate cancer. We hope this will improve patient experience and outcome by spending less time on and experiencing side effects of treatments that might not work, starting a different treatment earlier, and potentially reduce the cost to the NHS.

The VARIANT study is a feasibility study in which we will look at whether doctors and patients are willing to use the results of this blood test to decide on a treatment option. At this stage we do not know that the AR-V7 biomarker will lead to patients having better responses to treatments. However, we hope that the results of VARIANT will help us to plan a similar, but larger, study to find out if the AR-V7 blood test does result in better outcomes for patients and whether AR-V7 testing should be used in standard NHS practice. We hope that 70 patients will take part in VARIANT.

Why have I been invited to take part?

You have been diagnosed with advanced metastatic prostate cancer and have already been treated with hormone therapy (also known as androgen deprivation therapy, or ADT). Your disease has stopped responding to the current therapy and you are due a change in treatment plan.

Do I have to take part?

No, it is up to you to decide if you want to take part in VARIANT. If you do not want to take part, you will still get the standard treatment that has been arranged by your treating doctor.

Clark E, *et al. BMJ Open* 2019; 9:e034708. doi: 10.1136/bmjopen-2019-034708

If you agree to take part, you can change your mind and withdraw from the study at any time without having to give a reason.

What does taking part involve?

- If you decide to take part, you will be selected to have either:
  a) **treatment guided by AR-V7 blood test result**

     In this group, your treating doctor will receive the results of your AR-V7 blood test. Your treating doctor will tell you the result of the AR-V7 blood test and discuss this with you before arranging a treatment option for you. The results of the test may support you and your doctor in deciding whether next generation hormonal therapy or non-hormonal therapy would be more suitable for you.

  b) **treatment as usual**

     In this group, your doctor will arrange a treatment option with you as they usually would if you were not in the study. You and your treating doctor will NOT receive the results of your AR-V7 blood test. The results will only be looked at by the trial management team at the end of the study.
- You will have equal chance of being in group a) or group b) (a 50:50 chance). Your group will be selected by a computer. We call this 'randomisation'. Your doctor will not have any say over which group you are in.
- It is important to note that we are not testing a new treatment in this study. All VARIANT patients will receive one of the usual treatment options available to patients in the NHS. The study is looking at whether doctors and patients are willing to use the AR-V7 blood test and whether it is helpful in deciding **which** treatment option is most suitable for individual patients.

What will I have to do?

A member of the VARIANT team will discuss the study with you and answer any questions you may have. If you decide to take part, and your doctor confirms you are eligible for the study, you will be asked to sign a consent form.

As well as receiving the treatment arranged with your doctor, taking part in the study will mean (for ALL patients, that is patients in group a) and group b)):

- You will be asked to give a **blood sample on 3 occasions** (at the start of the study, after 12 weeks and 24 weeks). We would like to collect around 20 ml of blood (about 4 teaspoons full) each time.
- Some patients will be selected to give an **additional blood samples on one occasion** (at the start of the study). We would like to collect around 10 ml of blood (about 2 teaspoons full) for this sample. Patients will be randomly selected by a computer system to give this additional blood samples. The doctor or a member of their team will tell you if you have been selected to give this sample.

- On 2 of these visits, you will be asked to complete 2 **short questionnaires** about your quality of life. These will take around 15 minutes each to complete.
- Study visits will all take place during your usual hospital visits. There should be no extra visits to hospital required.
- We will use some information that is already collected about you as part of your standard clinical care. This includes information about your diagnosis, your treatment, results of scans and blood tests and your physical health. Taking part in VARIANT does not involve any extra scans to those you would receive normally.
- To take part in VARIANT, you will be asked to give your permission for your blood samples collected during this study to be stored at the Northern Institute of Cancer Research and used for future research as described below.

Study timeline in addition to standard treatment

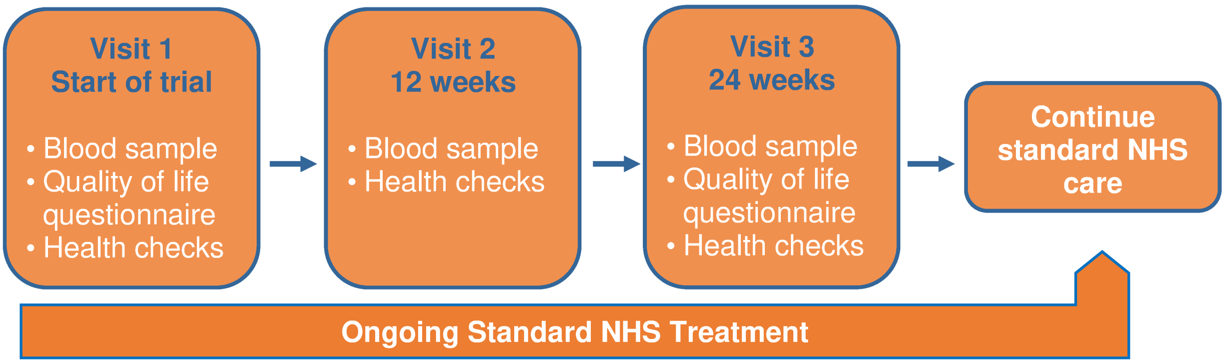

What will happen to my blood samples?

Your blood samples will be tested for the AR-V7 biomarker at the Northern Institute for Cancer Research (NICR). If you are selected to give the additional blood sample this will be sent to the All Wales Medical Genetics Laboratory (AWMGL) for testing. You and your treating doctor will only find out the AR-V7 biomarker result if you are selected to have your treatment guided by the AR-V7 blood test result.

After the AR-V7 test, any remaining sample will be stored at the NICR biobank (a collection of samples) and will be used in future research to look at why people develop advanced prostate cancer and how cancer reacts to treatments. If you do not want your samples to be stored or used through the NICR biobank you will not be able to take part in this trial.

Your samples will be labelled with a unique study number. This number is used to link your sample back to you and only the study team and authorised people will be able to do this. Your sample will be sent with a form that includes your initials, date of birth and the date the sample was taken. This will help us make sure the samples can be linked back to the correct person.

For more detailed information about what will happen with your blood samples please see the section 'Further information about what will happen to blood samples' on page X, we recommend that you read this.

Clark E, *et al. BMJ Open* 2019; 9:e034708. doi: 10.1136/bmjopen-2019-034708

Expenses and payments

You will not receive payment for taking part in VARIANT. All parts of the study will happen when you are already coming to hospital for your usual visits.

What happens when the research study stops?

Your appointment at 24 weeks will be the last time we see you for the study. At the end of the study you will continue to receive usual clinical care as decided by your hospital and doctor. If appropriate, this may include continuing the anti-cancer drugs you are receiving, or changing to another if you and your doctor believe this to be in your best interests.

What are the possible risks and benefits of taking part?

Risks: Taking blood samples may cause some discomfort and minor pain, and occasionally patients can feel faint during or after. Sometimes patients will have some bruising where the blood has been taken. Only trained members of staff will perform the blood tests and every effort will be made to prevent any discomfort.

VARIANT patients will receive one of the usual treatment options available to patients in the NHS. Your doctor will be able to tell you more about possible side effects of these treatments. The risk of these treatments is the same as if you were not taking part in the study.

Benefits: By taking part in VARIANT, you will be helping us gather information to learn about using the AR-V7 biomarker to guide treatment for patients with advanced metastatic prostate cancer. We hope that we can improve the quality of life of patients in the future from VARIANT.

For some patients, taking part in the study will mean that your doctor receives your AR-V7 biomarker result before deciding on a treatment for you. This may help to inform which treatment would be best for you after reviewing your medical history.

If you want to find out more about taking part in research studies, you can visit the NHS Choices Website www.nhs.uk/conditions/clinical-trials/. On this website you will also find contact details for your local Patient Advice Liaison Service (PALS) office if you would like to speak to someone.

Further supporting information

What will happen if I do not want to carry on in the study?

You can withdraw your consent at any time and for any reason, without having to tell us your reason. You will be fully cared for and supported as per your hospital's standard practice.

We will ask if you are happy for us to:

- Use any information already collected about you.
- Continue using information collected as part of your usual care until the end of the study.

Clark E, *et al. BMJ Open* 2019; 9:e034708. doi: 10.1136/bmjopen-2019-034708

You can change your mind about allowing your stored blood samples to be used for research at any time in the future, without giving any reasons, by contacting your local study team as listed on page x. Any samples left at the NICR will be destroyed. Any researchers to whom samples have been sent will be instructed to destroy the samples they have in their laboratories. It will not be possible to withdraw any findings from research work already undertaken using your donated samples.

Can I take part in other research?

If you are already taking part in a clinical trial we would look to see what the trial involved and speak to the other trials team. We would need check that taking part in one trial would not affect the treatment or results in the other.

If you take part in VARIANT and want to join another trial in the future, we ask that you let the VARIANT team know so we can check with their team that there is no conflict. If there was a conflict, the study team would discuss your options with you and you can decide what it best for you.

What if there is a problem?

If you are not happy with any part of VARIANT, you should ask to speak to the study team first who will do their best to help you. **Their contact details are on page X.** If you are still unhappy you may wish to raise your concerns with someone who is not directly involved in your care. You can contact the Patient Advice Liaison Service (PALS) who provide a confidential service on <site to localise with phone number and email address>

In the unlikely event that you are harmed during the research and this is due to someone's negligence (they were careless) you may have grounds for legal action for compensation, but you may need to meet your own legal costs. NHS indemnity does not offer no-fault compensation (for harm that is not anyone's fault).

Will my GP be told about my involvement in VARIANT?

Yes, with your permission we will inform your GP that you are taking part in VARIANT. We will send you a copy of this letter so that you can see exactly what has been said. It will also be noted in your hospital medical records so that staff in the hospital know you took part in the study.

What will happen to the results of the study?

- The study is due to finish at the end of 2020.
- The results will be written in medical journals and presented in meetings to other doctors, nurses and researchers.
- The anonymised data might be shared with other researchers and to help with future studies. Your identity will always be protected.
- A report will be written by the study funder and put on their website.

Clark E, *et al. BMJ Open* 2019; 9:e034708. doi: 10.1136/bmjopen-2019-034708

- You may request a summary of the results at the end of the trial by contacting the study team, their details are on page X.

Will the information about me be kept confidential?

Yes. All of the information collected will be entered on computers that are kept secure and password protected.

- We will use a study number to identify you instead of using your name.
- Your contact details will never be shared with anyone else.
- You will not be named in any published results, reports or anything on our website.

The study information about you and your medical notes will be looked at by people directly involved in the study, clinical and lab staff as well as by people who are checking the study is running as it should. This may include the Newcastle Clinical Trials Unit at Newcastle University as they are managing the study. It may also include regulatory authorities, sponsor and funder.

Your local study team will collect information from you during the study. This will be entered onto a secure database using your study number instead of your name. Your local study team will be the only people that know that the study number refers to you. This means that the data on the database it is not identifiable to anyone outside of your local study team. The database is held by the Newcastle Clinical Trials Unit at Newcastle University. Access to this database is password protected and available to your doctors and research staff for the purpose of the trial. Data from the database will be analysed at the end of the whole study. Anonymised data from the database may be used when applying to carry out future related clinical trials or for other research projects related to prostate cancer.

After you have finished attending study visits, your local study team may review your health records and collect information for the study.

Data Protection

All of the data we collect will be kept strictly confidential and in accordance with the General Data Protection Regulation (GDPR).

We have included information on page X of this document that tells you how we do this and included some websites where you can find out more.

Who is organising and funding VARIANT?

The main study doctor (also called the 'Chief Investigator') is Mr Rakesh Heer, a Urological Surgeon at The Newcastle upon Tyne Hospitals NHS Foundation Trust. The study team includes senior doctors and nurses, university experts in research studies, and members of the public.

It is managed by the Newcastle University Clinical Trials Unit on behalf of the study sponsor – The Newcastle upon Tyne Hospitals NHS Foundation Trust. It is funded by the National Institute for Health Research, Research for Patient Benefit programme.

Clark E, *et al. BMJ Open* 2019; 9:e034708. doi: 10.1136/bmjopen-2019-034708

At least 3 hospitals will be taking part in VARIANT. Each hospital has a study doctor, called an 'Investigator'. The Investigator in your hospital is ………………………….

Who has reviewed the study?

All research in the NHS is looked at by an independent group of people called a Research Ethics Committee. VARIANT has been reviewed and given a favourable opinion by the Wales REC 2 committee.

Patients have been involved in deciding how to do VARIANT from the start. For example, patients were involved in designing and applying for funding for the study, and continue to contribute as part of the study team. We also asked a group of patients and carers who have experienced cancer to look at the study information sheet to check the study is described in a clear way and is easy to understand.

What if relevant new information becomes available?

The treatment management of metastatic prostate cancer is changing all of the time. All VARIANT patients will receive one of the standard treatment options available in the NHS. This will include any new treatment options that become available while the study takes place.

Information gathered during the course of this study will be reviewed by an independent Trial Oversight Committee. The role of this group is to protect the safety and wellbeing of participants by making sure the study is running safely.

What if I have any questions?

Please ask the doctor or nurse who is looking after you. They can put you in touch with the research team or the Investigator for VARIANT at your hospital.

What happens next?

You can take time to think about the study and whether you want to take part. A member of the research team will speak to you when you come back in to discuss your treatment options. They will go through this information sheet with you and answer any questions before you make your final decision.

Thank you for taking the time to read this information sheet.

VARIANT team contact details for your hospital:

Principal Investigator:
Address:

Tel:

Research Nurse:
Address:

Tel:

Clark E, *et al. BMJ Open* 2019; 9:e034708. doi: 10.1136/bmjopen-2019-034708

GDPR and Transparency Information

The Newcastle upon Tyne Hospitals NHS Foundation Trust (NUTH) is the sponsor for this study based in the United Kingdom and will act as the “data controller” for this study. **They are responsible for looking after your information and using it properly.**

This study is managed on behalf of the sponsor by the Newcastle Clinical Trials Unit who will act as the “data processor”. As data processor, this means that we are responsible for processing personal data on behalf of a controller. We will be using information from you in order to undertake this study, and will keep identifiable information about you for 10 years.

Your rights to access, change or move your information are limited, as we need to manage your information in specific ways in order for the research to be reliable and accurate. If you withdraw from the study, we will keep the information about you that we have already obtained. To safeguard your rights, we will use the least amount of personally-identifiable information possible.

You can find out more about how your information is used at http://www.newcastle-hospitals.org.uk/about-us/freedom-of-information_how-we-use-information

To find out more about research and general use of patient information please refer to the Health Research Authority Website https://www.hra.nhs.uk/information-about-patients/

The sponsor as an NHS Organisation and the Newcastle Clinical Trials Unit as a University use personally-identifiable information to conduct research to improve health, care and services. As a publicly-funded organisation, we have to ensure that it is in the public interest when we use personally-identifiable information from people who have agreed to take part in research. This means that when you agree to take part in a research study, we will use your data in the ways needed to conduct and analyse the research study.

Health and care research should serve the public interest, which means that we have to demonstrate that our research serves the interests of society as a whole. We do this by following the UK Policy Framework for Health and Social Care Research.

If you wish to raise a complaint on how we have handled your personal data, you can contact our Data Protection Officer who will investigate the matter. If you are not satisfied with our response or believe we are processing your personal data in a way that is not lawful you can complain to the Information Commissioner’s Office (ICO).

The sponsor Data Protection Officer is Richard Oliver and you can contact them at nuth.dpo@nhs.net.

The local study team at your hospital will collect information from you and/or your medical records for this research study in accordance with our instructions.

The local study team will use your name, NHS number and contacts details to contact you about the research study, and make sure that relevant information about the study is recorded for your care, and to oversee the quality of the study. Individuals from the sponsor, Newcastle Clinical Trials Unit and regulatory organisations may look at your medical and research records to check the accuracy of the research study. The local

Clark E, *et al. BMJ Open* 2019; 9:e034708. doi: 10.1136/bmjopen-2019-034708

study team will pass these details to the sponsor or the Newcastle Clinical Trials Unit along with information collected from you and/or your medical records. The only people at sponsor or the Newcastle Clinical Trials Unit who will have access to information that identifies you will be people who need to contact you or audit the data collection process. The people who analyse the information will not be able to identify you and will not be able to find out your name, NHS number or contact details.

The local study team will keep identifiable information about you from this study for 10 years after the study has finished.

When you agree to take part in a research study, the information about your health and care may be provided to researchers running other research studies in this organisation and in other organisations. These organisations may be universities, NHS organisations or companies involved in health and care research in this country or abroad. Your information will only be used by organisations and researchers to conduct research in accordance with the UK Policy Framework for Health and Social Care Research.

This information will not identify you and will not be combined with other information in a way that could identify you. The information will only be used for the purpose of health and care research, and cannot be used to contact you or to affect your care. It will not be used to make decisions about future services available to you, such as insurance.

Clark E, *et al. BMJ Open* 2019; 9:e034708. doi: 10.1136/bmjopen-2019-034708

Further information about what will happen to blood samples

- Your samples will be sent securely to the Northern Institute of Cancer Research (NICR) for testing. For selected patients, a sample will also be sent to the All Wales Medical Genetics Laboratory (AWMGL) for testing (to confirm the results of the biomarker test).
- At the start of the study, your blood sample will be tested for the AR-V7 biomarker. Your doctor will receive the result of this test if you are in the group receiving treatment guided by AR-V7 blood test result. If you are in the treatment as usual group neither you nor your doctor will receive the result of the test.
- We may also test your blood sample for the AR-V7 biomarker at the end of the trial. These results will be used to inform the research. Neither you nor your doctor will not receive these results.
- Your blood samples will be stored at the NICR biobank (a collection of samples) and will be used in future research to look at why some patients develop advanced prostate cancer or how the cancer reacts to treatment. The DNA and RNA (the genetic material inside a cell), will be taken out of these stored samples and will also be stored at the NICR biobank. If you do not want your samples to be stored or used through the NICR biobank you will not be able to take part in this trial.
- Although unlikely for the VARIANT trial, there is a small possibility that your blood samples which have been stored in the NICR biobank may be used in research involving rodents (rats or mice). This is only done when it is essential to further our understanding of the way in which a disease develops or responds to treatment. These experiments are performed according to strict guidelines set out by the government and involve minimal stress to the rodents. **If you do not wish for your samples to be used in research using animals you should not complete the box referring to this on the consent form we will ask you to sign.** If you do not consent to your samples being used in research using animals you will still be able to participate in the VARIANT trial.
- If you are selected to provide an additional sample to be sent to AWMGL, the DNA and RNA (the genetic material inside a cell) may be taken out and stored at AWMGL before being transferred to the NICR biobank.
- You and your doctor will not find out the results of any tests done on your stored blood samples. Results of future research with your stored samples will be used to improve care of patients with advanced prostate cancer in the future.
- Your blood samples will be labelled with a unique study number. This number is used to link your sample back to you and only the study team and authorised people will be able to do this. Your sample will be sent with a form that includes your initials, date of birth and the date the sample was taken. This will help us make sure the samples can be linked back to the correct person which is especially important for feeding back the test results.
- Your stored samples may be used by researchers in the UK or overseas (including USA or Europe). Commercial partners may also use your samples for research

Clark E, *et al. BMJ Open* 2019; 9:e034708. doi: 10.1136/bmjopen-2019-034708

purposes. In most cases the commercial partners will be small companies that were started in universities (university/academic spin-off companies), or drug companies. Working with commercial partners is often important to have the resource to develop tests or products. We will ask partners to sign a legal contract to make sure samples are handled appropriately. Although the research will not be conducted to make money, it is possible that some of the results will be of value to commercial companies, for example in the development of new tests or treatments

- Any money made by sending samples to commercial laboratories will be put in to local research or used to improve patient care. Under UK law, sample donors are not entitled to a share of any profits that may result from this activity.
- The samples will be destroyed after 10 years of the last follow up for the last patient enrolled in the study. This will include all the blood samples and any linked data.

Clark E, *et al. BMJ Open* 2019; 9:e034708. doi: 10.1136/bmjopen-2019-034708

Consent Form

VARIANT V7

To be printed on local headed paper

The Variant 7 Biomarker Feasibility Study

The **VARIANT** Study

PATIENT CONSENT FORM

Site ID number: ☐☐ **Participant ID Number:** ☐☐☐

Principal Investigator Name: _______________

Please INITIAL these boxed if you agree:

1. I have read and understood the Patient Information Sheet version _______ dated______ for the above study. I have had the opportunity to consider the information, ask questions and I am happy with the answers given.	
2. I understand that I do not have to take part in this study. I know that I can withdraw at any time without giving a reason and without my medical care or legal rights being affected.	
3. I understand that information about me will be collected, recorded and used for this study unless I withdraw my consent. I understand that my information will be kept securely and confidentially.	
4. I agree for a copy of my consent form to be sent securely to the Newcastle University Clinical Trials Unit for checking.	
5. I agree to my General Practitioner (GP) being informed about me taking part in this study.	
6. I understand that relevant sections of my medical notes and information collected during the study may be looked at by people from Newcastle Clinical Trials Unit, the study sponsor, regulatory authorities and the local NHS Trust, where it is relevant to my taking part in this research. I give permission for these individuals to have access to my records.	

Clark E, *et al. BMJ Open* 2019; 9:e034708. doi: 10.1136/bmjopen-2019-034708

Consent Form

7. I understand that any personal information collected for the study will be kept confidential and not be made public. I understand that anonymised data from the study may be used for other research projects related to prostate cancer and will be published in medical journals, at research meetings and shared with other researchers.	
8. I consent to relevant sections of my medical notes being accessed by members of my local study team to carry out follow-up, including after my participation in the trial has ended, and for there to be a link between my healthcare records and the data collected for the trial.	

Sample collection

9. I consent to the collection of my blood samples as described in the VARIANT patient information sheet and understand that my samples will be sent to the Northern Institute of Cancer research (NICR) and the All Wales Medical Genetic Lab (AWMGL) for testing.	
10. I understand that my samples will not be identifiable except to the trial management team and I give permission for my date of birth and initials to be sent with the blood samples to the lab and with the biomarker result to my study doctor.	
11. I consent to my samples being stored in the NICR biobank for up to 10 years, and give permission for samples to be used as described in the VARIANT Patient Information Sheet. I understand that the biobank will keep my identity confidential and any information collected about me during the study will be anonymised in a way that protects my identity.	
12. OPTIONAL: I consent to my samples to be used in experiments using rodents (rats or mice).	

Agree to Participate

13. I agree to take part in the VARIANT study.	

______________________ ________________ ________________

Name of patient **Signature** **Date**

______________________ ________________ ________________

Name of Person **Signature** **Date**

Clark E, *et al. BMJ Open* 2019; 9:e034708. doi: 10.1136/bmjopen-2019-034708

Consent Form

taking consent

When completed: 1 copy for participant; 1 copy (original) for Investigator Site File; 1 copy to be kept in medical notes and 1 copy to be sent securely to Newcastle Clinical Trials Unit

Clark E, *et al. BMJ Open* 2019; 9:e034708. doi: 10.1136/bmjopen-2019-034708
