# Supplementary material for: Prostate cancer androgen receptor splice variant 7 biomarker study - a multicentre randomised feasibility trial of biomarker-guided personalised treatment in patients with advanced prostate cancer (the VARIANT trial) study protocol
Source: BMJ Open. 2019 Dec 18;9(12):e034708. doi: 10.1136/bmjopen-2019-034708 (PMC6937062; doi:10.1136/bmjopen-2019-034708)
Supplement: Author's manuscript [file bmjopen-2019-034708.draft_revisions.pdf]

BMJ Open

BMJ Open is committed to open peer review. As part of this commitment we make the peer review history of every article we publish publicly available.

When an article is published we post the peer reviewers' comments and the authors' responses online. We also post the versions of the paper that were used during peer review. These are the versions that the peer review comments apply to.

The versions of the paper that follow are the versions that were submitted during the peer review process. They are not the versions of record or the final published versions. They should not be cited or distributed as the published version of this manuscript.

BMJ Open is an open access journal and the full, final, typeset and author-corrected version of record of the manuscript is available on our site with no access controls, subscription charges or pay-per-view fees (http://bmjopen.bmj.com).

If you have any questions on BMJ Open's open peer review process please email info.bmjopen@bmj.com

BMJ Open

# The Prostate Cancer Androgen Receptor Splice Variant 7 Biomarker Study - A multicentre randomised feasibility trial of biomarker-guided personalised treatment in patients with advanced prostate cancer (The VARIANT Trial) study protocol.



| | |
|---|---|
| Journal: | *BMJ Open* |
| Manuscript ID | bmjopen-2019-034708 |
| Article Type: | Protocol |
| Date Submitted by the Author: | 02-Oct-2019 |



| | |
|---|---|
| Complete List of Authors: | Clark, Emma; Newcastle University, Northern Institute for Cancer Research<br>Morton, Miranda; Newcastle University, Newcastle Clinical Trials Unit<br>Sharma, Shriya; Newcastle University, Newcastle Clinical Trials Unit<br>Fisher, Holly; Newcastle University, Institute for Health and Society<br>Howel, Denise; Newcastle University, Institute of Health & Society<br>Walker, Jenn; Newcastle University, Newcastle Clinical Trials Unit<br>Wood, Ruth; Newcastle Clinical Trials Unit,<br>Hancock, Helen; Newcastle University, Newcastle Clinical Trials Unit<br>Maier, Rebecca; Newcastle University, Newcastle Clinical Trials Unit<br>Marshall, John<br>Bahl, Amit; University Hospitals Bristol NHS Foundation Trust<br>Crabb, S; University of Southampton<br>Jain, Suneil; Queen's University Belfast<br>Pedley, Ian; Newcastle Upon Tyne Hospitals NHS Foundation Trust<br>Jones, Rob; University of Glasgow<br>Staffurth, John; Velindre Cancer Centre, Research; Cardiff University School of Medicine, Division of Cancer and Genetics<br>Heer, Rakesh; Northern Institute for Cancer Research |



| | |
|---|---|
| Keywords: | Protocols & guidelines < HEALTH SERVICES ADMINISTRATION & MANAGEMENT, Prostate disease < UROLOGY, Adult oncology < ONCOLOGY, Urological tumours < ONCOLOGY, Cell biology < BASIC SCIENCES |
| | |


SCHOLARONE™
Manuscripts

# Title

The Prostate Cancer Androgen Receptor Splice Variant 7 Biomarker Study - A multicentre randomised feasibility trial of biomarker-guided personalised treatment in patients with advanced prostate cancer (The VARIANT Trial) study protocol.

Authors: Emma Clark[1], Miranda Morton[2], Shriya Sharma[2], Holly Fisher[3], Denise Howel[3], Jenn Walker[2], Ruth Wood[2], Helen Hancock[2], Rebecca Maier[2], John Marshall[4], Amit Bahl[5], Simon Crabb[6], Suneil Jain[7], Ian Pedley[8], Rob Jones[9*], John Staffurth[10]* and Rakesh Heer[1]*
*Joint senior authors

1. Northern Institute for Cancer Research, Newcastle University, Newcastle Upon Tyne, UK
2. Newcastle Clinical Trials Unit, Newcastle University, Newcastle Upon Tyne, UK
3. Institute for Health and Society, Newcastle University, Newcastle Upon Tyne, UK
4. VARIANT Trial patient representative
5. University Hospitals Bristol NHS Foundation Trust
6. University of Southampton
7. Queen's University Belfast
8. The Newcastle upon Tyne Hospitals NHS Foundation Trust
9. University of Glasgow
10. Velindre Cancer Centre, Cardiff University, Cardiff, UK

Corresponding authors:

1. Emma Clark:

• e-mail: emma.clark@ncl.ac.uk

• Phone: 0191 208 4456

2. Rakesh Heer:

• e-mail: rakesh.heer@ncl.ac.uk

• Phone: 0191 208 4300

Key Words:

- Prostate Disease
- Protocols and guidelines
- Adult Oncology
- Urological tumours
- Cell biology

This study opened to recruitment on the 09/07/2019 with the first patient consented 29/07/2019 and is expected to report in 18 months. This protocol is the current approved VARIANT protocol Version 2.0 8th March 2019



For peer review only - http://bmjopen.bmj.com/site/about/guidelines.xhtml

## Abstract

### Introduction:

Prostate Cancer is the most common male cancer with 1 in 4 developing non-curable metastatic disease. Initial treatment responses to hormonal therapies are transient and further management options lie between (1) further hormone therapy or (2) a non-hormonal approach involving additional chemotherapy or molecular radiotherapy (radium-223). There is no clear rationale for choosing between these mechanistically different treatment approaches. The biology of hormone resistance is driven through abnormal androgen receptor activity and we can assay this through a blood test measuring androgen receptor variant 7 (AR-V7) expression in circulating tumour cells (CTCs). Despite increasing evidence supporting AR-V7's role as a prognostic marker, the clinical utility of such measures remains unknown in helping personalise treatment decisions.

### Methods and Design:

The VARIANT feasibility trial is a pragmatic design, to be run over 18 months with participants randomised into the intervention arm receiving biomarker (AR-V7) guided clinical treatment and participants randomised into the control arm with conventional standard management (no biomarker guidance). AR-V7 positive participants (likely to be insensitive to further hormone treatment) will receive chemotherapy or in other cases radium-223 (where routinely available). Seventy male ≥18 years old participants with metastatic castrate resistant prostate cancer clinically indicated to proceed to further hormone therapy or chemotherapy will be recruited from three National Health Service (NHS) Trusts based in England, Scotland and Wales. The feasibility primary outcome is willingness of patients to be randomised and clinicians to recruit to a biomarker-based treatment strategy, with trial data informing the basis of a definitive and appropriately powered randomised control trial (RCT).

### Ethics and Dissemination:

Formal ethics review was undertaken with a favourable opinion, through Wales NRES Committee 2 18/WA/0419. Findings to be disseminated through patient and professional organisations that have expressed their support, media outlets and peer-reviewed journal publication.

## Article Summary

### Strengths and limitations of this study:

- **Focuses on a priority area of need in advanced prostate cancer clinical practice.**
- **To date, the feasibility of delivering a randomised biomarker guided-treatment trial in prostate cancer to formally assess clinical utility is not established and will be addressed in this study.**
- **As a feasibility study, the planned sample size (70 participants) does not have sufficient power or precision to compare the 'event' rate between treatment arms, but will allow informed planning for a definitive randomised controlled trial with prominent clinicians from non-recruiting centres involved in feasibility to aid with follow-on trial.**



For peer review only

For peer review only - http://bmjopen.bmj.com/site/about/guidelines.xhtml

- **Emerging evidence points to additional Androgen Receptor (AR) biology driving hormone resistance, such as other variant expression and mutations – these along with alternative biomarkers can be explored in the associated biobanked samples (including cell free tumour DNA).**
- **Strong patient and public involvement to inform study design with a clear commitment to informing participants of project outcomes setting a clear new gold standard for PPI.**

## INTRODUCTION

### Background

Prostate cancer (PC) is the most common male cancer in the UK and the second highest cause of male cancer death(1). In large part, PC is a slowly progressive disease and when detected at an early stage is managed by active surveillance, surgery or radiotherapy. However, 25% of patients will present with, or will progress to, advanced metastatic PC(2,3). Metastatic PC is incurable, with less than one third of patients surviving more than 5 years(1).

Medical castration (commonly referred to as hormonal treatment or androgen deprivation therapy (ADT)), blocks production of the hormone testosterone and/or targets the androgen receptor (AR) signalling axis that drives cancer cell growth. Although a good response to hormonal treatment seen often initially, disease progression to a lethal metastatic Castration-Resistant Prostate Cancer (mCRPC) is common(4). Clinical trials have shown that the addition of chemotherapy (docetaxel) or other hormonal approaches (abiraterone acetate or enzalutamide) to initial hormonal therapy have led to a substantial improvement (i.e. delay) in time to the development of mCRPC and overall survival (OS)(8-9). Furthermore, promising recent evidence from randomised trials of androgen-receptor axis-targeted drugs (ARATs) have shown addition of Apalutamide (an inhibitor of the ligand-binding domain of the AR) alongside hormone therapy results in longer overall survival and radiographic progression free survival compared to placebo(10). However despite these rapid advances, mCRPC typically manifests within 3 years and is uniformly fatal(11-13).

### Treatment management for mCRPC

Management pathways for mCRPC are still evolving in response to emerging new treatments however, it broadly follows one of two standard care approaches(14): (1) further hormonal treatment such as abiraterone or enzalutamide or (2) 'non-hormonal' treatment, typically chemotherapy or molecular radiotherapy (radium 223) (where available). There is no clear biological rationale for choosing between these mechanistically different treatment approaches. Suitable patients for this study can receive both approaches in a sequential manner if one is failing. Patients and clinicians often prefer hormonal treatment, being less toxic and easier to manage, however, only 30-50% of men respond well, with the remainder demonstrating a poor or an equivocal response(15,16). As many patients will not respond to either treatment approach, there are considerable costs from our current management pathways, both in terms of patient experience and outcomes (side effects and disease progression) and economic costs to the NHS (large burden of expensive treatments for the commonest male cancer). Personalised management pathways are urgently needed.

### Biology of the Androgen Receptor (AR selective treatment pressure)



For peer review only - http://bmjopen.bmj.com/site/about/guidelines.xhtml

A breakthrough in understanding the biology of PC revealed that hormonal treatments generate a selective pressure at the cellular level inducing complex molecular mechanisms characterised by an adaptation of the androgen-AR signalling axis. This results in tumour resistance mediated by the induced expression of alternative types of androgen receptor. These AR mRNA splice variants lack the important hormone-binding domain, resulting in a constitutively active cellular receptor, despite castration. The most widely studied variant is AR-V7(17,18). AR-V7 activity is not affected by 'hormonal treatment' such as enzalutamide and abiraterone that target the hormone-binding domain, potentially rendering these treatments ineffective in men with AR-V7(19-21). A surge in ARATs available for clinical use (e.g. Apalutamide and Darolutamide) will most likely enhance this burden (although of note, evidence demonstrating reduced effectiveness of these treatment in men who are positive for AR-V7 or other variant splice forms including AR point mutations, have not been published to date).

**Rationale**

Published clinical data demonstrates a strong link between AR-V7 expression and mCRPC progression and highlights the potential for AR-V7 to be utilised as a treatment stratification biomarker to identify those men likely to be sensitive to further hormonal treatment (AR-V7-ve patients) and avoid futile treatments in those predicted to be insensitive (AR-V7+ve patients)(21-29). Notably AR-V7 positivity is not associated with insensitivity to taxane chemotherapy treatment (relative reduction in risk of death of 76% maintained with chemotherapy, hazard ratio: 0.24; 95% CI, 0.10-0.57; P = 0.035)(27,30) and data from the recent PROPHECY trial reports on the prognostic value of the AR-V7 biomarker (prospective observational cohort of poor prognosis patients with advanced prostate cancer who receive abiraterone or enzalutamide treatment)(31). The commercially available AdnaTest ProstateCancerPanel AR V7 assay (Qiagen®) detects AR-V7 mRNA expression in circualtung tumour cells (CTCs) in whole blood and has been independently and robustly clinically validated in terms of reproducibility and comparisons of sensitivity and specificity with other AR-V7 detection platforms(31,41-44). However to date, there have been no formal measures of the clinical utility of AR-V7 as a predictive biomarker

**Evidence gap**

Encouragingly, a cost saving analysis of performing ProstateAdnaTest AR-V7 biomarker testing in mCRPC demonstrated use of the biomarker would result in a substantial cost saving as long as the true prevalence of AR-V7 was >5% (well below the accepted prevalence rate of 30%)(45). However, formal cost effectiveness analyses based on incremental cost-effectiveness ratio (ICER) (cost per quality-adjusted life year gained) and assessing prevalence rates of this biomarker have yet to be carried out.

The National Comprehensive Cancer Network Task Force(32) and CRUK consensus statement on biomarker roadmap for cancer studies(33) have highlighted the key recommendations for accelerating a tumour biomarker into clinical practice by sequentially demonstrating evidence for; (1) analytic reproducibility; (2) clinical validity and; (3) clinical utility. Previous clinical studies on AR-V7 testing focused on retrospective or prospective cohort analyses of associated AR-V7 expression distinguishing subgroups with different clinical outcomes with hormonal treatment in men with metastatic PC(23,26,29,30,34-38). However, the highest level of assessment of clinical worth in improving patient outcomes (clinical utility) remains lacking.



For peer review only - http://bmjopen.bmj.com/site/about/guidelines.xhtml

We have paid particular focus to address clinical utility evidence gaps in the VARIANT trial using published levels of evidence standards for assessing biomarkers to inform study design. We aim to demonstrate improvement in patient outcome sufficiently to justify AR-V7 biomarker incorporation into routine clinical care (including feasibility of collecting quality of life measures for a future health economic evaluation)(39,40).

**Emerging treatment landscape**

The treatment landscape of hormone sensitive (PC) is evolving, altering treatment pathways for mCRPC. Recent data from the USA based PROPHECY trial reporting on a prospective observational cohort showed mRNA AR-V7 (modified Qiagen ProstateCancerAdnaTest, Baltimore, MD) and protein AR-V7 (Epic nuclear-specific, San Diego) biomarker positivity associated with worse progression free survival (PFS) and overall survival (OS) in poor prognosis patients with advanced prostate cancer who receive abiraterone or enzalutamide treatment (31). Criticisms of the study included lack of testing with alternative treatment such as chemotherapy (which we have addressed in this study) and pre-selection of high risk CRPC patients (i.e. those with poor prognosis), ultimately generating results that cannot be extrapolated over the overall CRPC population (46-49). Of note, lower AR-V7 prevalence was reported in the overall CRPC population  in the ARMOR3-SV phase III clinical trial which employed the Adnatest ProstateCancerSelect and Detect CTC assay (Qiagen®) to assess AR-V7 mRNA expression, where only 8% of men were AR-V7 positive (95% CI 6-10)(42,43).

We argue however, irrespective of the evolving treatment landscape, the opportunity to generate feasibility data for a biological (biomarker) informed approach to treatment selection over standard care protocol-based approaches, tests a highly relevant clinical question in these high risk CRPC patients (i.e. those who have more to loose from pursuing a 'try and see' approach). This would provide an appealing long-term strategy (for patients and service providers) to ultimately improve on clinical outcome (specifically for a clinical subgroup of poor prognosis patients, identifying those likely to be sensitive to further hormonal treatment and avoid futile treatments in those that are predicted to be insensitive).

**<u>Main Aim of Study</u>**

To determine the feasibility of conducting a definitive randomised control trial to evaluate the clinical utility of an AR-V7 blood biomarker assay in personalising treatment for men with mCRPC in UK NHS clinical practice.

**<u>Objectives</u>**

Feasibility study

**Primary:**

1. To establish if it is feasible to conduct a definitive trial comparing AR-V7 biomarker-driven management with the current standard care in patients with mCRPC.

**Secondary Objectives:**

2. To estimate AR-V7 biomarker prevalence in the trial population to inform sample size calculations for a definitive randomised control trial.



For peer review only - http://bmjopen.bmj.com/site/about/guidelines.xhtml

3. To assess recruitment, compliance and retention rates
4. To confirm outcome measures for a future definitive trial and establish trial data response rates, variability, and data quality.
5. To establish a blood sample biorepository to include baseline, 12 and 24 week blood samples for future translational studies.

**Exploratory objectives:**

6. To establish a complete serial blood tissue archive to include potential measures of cell free DNA and additional AR-Variants in CTCs and cfDNA biomarker measures (such as AR mutations, other AR splice forms and AR amplification and other mutations such as PTEN/p53/MYC gain/RB1 loss/MET gain and further molecular pathways yet to be defined) to complement AR-V7 reads, depending upon the ultimate biomarker performance characteristic established in this trial population. Blood will be collected, processed and archived at 0 weeks (baseline), 12 weeks and 24 weeks following the first treatment.
7. To explore thresholds of the magnitude of AR-V7 positivity to investigate relationships with outcomes and to estimate AR-V7 positivity rate assumptions regarding a cut-off point.
8. To undertake cross site validation of biomarker reads between two GCP laboratories (NICR, Newcastle and AWMGL, Cardiff).

## METHODS AND ANALYSIS

### Study design

This feasibility study is a multi-centre, two-arm, randomised control trial (RCT). All patients who consent to take part in the trial and who are eligible, will have a blood test to assess prevalence rate of the AR-V7 biomarker. Participants will be randomised in the ratio 1:1 to receive personalised standard treatment (intervention) guided by AR-V7 biomarker status or standard care (control) without biomarker guided treatment. Those in the control group will not receive blood biomarker test results.

The treatment for each patient is expected to be dependent on various factors (e.g. clinician choice, patient choice, previous treatments, co-morbidities, concomitant medication and pattern of disease) as well as randomised allocation and AR-V7 status in the personalised treatment arm. All treatments are part of standard care for these participants. Treatment options for participants randomised to the personalised standard treatment arm will be recommended, but not mandated within this feasibility trial, with reasons for not following the recommendation recorded and reported as outcome. A CONSORT diagram of study protocol (version 2.0 8$^{th}$ March 2019) is shown in figure 1.

### Study Setting

Seventy patients with mCRPC who require a change in treatment will be recruited in three secondary care NHS Trusts in the UK spread across England (The Newcastle upon Tyne Hospitals NHS Foundation Trust), Scotland (NHS Greater Glasgow and Clyde) and Wales (Velindre University NHS Trust). We aim to recruit mCRPC patients with a predicted poor overall survival. We anticipate this group of mCRPC patients have the most to gain from a



For peer review only - http://bmjopen.bmj.com/site/about/guidelines.xhtml

biological-based treatment approach as their disease is more likely to progress during a period of treatment with an inactive agent. Multivariate analysis from the metastatic population of STAMPEDE(52) has shown that worse overall survival was seen in men with the following features: presence of bone metastases (regardless of soft tissue metastases), worse WHO performance status (0 vs 1 or 2), higher (or unknown) initial Gleason sum score category (≥8 vs ≤7), and younger age at randomisation <60yrs. Poorer failure free survival (but not overall survival was additionally seen in men with worse primary tumour stage and higher PSA level before starting ADT. There is overlap between these poor prognostic features and factors associated with a high likelihood of harbouring AR-V7+ve CTCs(43,44).

**Eligibility Criteria**

Patients will be aged ≥18 years old with metastatic castrate resistant prostate cancer (high risk features) clinically indicated to proceed to further hormone therapy or chemotherapy and fulfil all of the following criteria:

1. Histologically or cytologically proven diagnosis of adenocarcinoma of the prostate.
2. Radiographic and/or histological and/or cytological evidence of metastatic disease.
3. Castrate levels of testosterone and documented ongoing medical or surgical castration. Testosterone level ≤50ng/dl /1.73 nmol/L and maintaining on androgen suppression therapy
4. Disease progression since the last change in therapy defined by one or more of the following: (i) PSA progression as defined by the prostate cancer working group 3 (PCWG3) criteria ≥ 2ng/ml; (ii) bone disease progression as determined by the local radiology/ multidisciplinary team; (iii) radiographic progression of nodal or visceral metastases as determined by the local radiology/ multidisciplinary team.
5. Suitable for treatment with at least one novel hormonal treatment (with available treatments abiraterone acetate or enzalutamide) and one non-hormonal therapy (with available treatments docetaxel, cabazitaxel or radium-223).
6. At least two high risk features: (i) age <60 years at time of diagnosis of metastatic disease; (ii) bone metastases present at time of initial metastatic prostate cancer diagnosis (although not mandated, it is considered good clinical practice to have up to date imaging within 8 weeks); (iii) Gleason grade group 4 or 5 (Gleason score 8 to 10); (iv) presence of visceral metastases (e.g. liver or lung) at any time point. This does not include lymph node metastases; (v) PSA doubling time < 3 months; (vi ) elevated alkaline phosphatase above institutional upper limit of normal; (vii) ECOG Performance Status worse than or equal to 1; (viii) previous treatment for castration resistant prostate cancer with docetaxel chemotherapy; (ix) previous treatment for castration resistant prostate cancer with abiraterone and/or enzalutamide or equivalent agent.
7. Estimated life expectancy > 6 months.
8. Provision of written informed consent, including consent for bio-banking of blood samples.



For peer review only - http://bmjopen.bmj.com/site/about/guidelines.xhtml

Exclusion Criteria applied in the VARIANT trial are:

1. Histological variants of prostate cancers with small cell or neuroendocrine features.
2. Prior or current malignancy (except adenocarcinoma of the prostate) with an estimated ≥ 30% chance of relapse/progression within next 2 years.
3. Previously identified brain metastases or spinal cord compression unless treated with full functional recovery.
4. Administration of an investigational agent within 30 days of first dose of trial medication.

**Randomisation**

Patients will be randomised to receive either personalised standard treatment (guided by AR-V7 biomarker status) or standard care (not guided by biomarker status) on a 1:1 basis using a method of random permuted blocks of concealed variable block size and stratified by site.

**Study Intervention**

This three-centre randomised feasibility study incorporates a control and an intervention arm. All patients will undergo AR-V7 biomarker assessment with results only made known to the patients and clinical team in the intervention arm.

**Intervention arm**

Treatment will be given as per standard care with recommendations guided by biomarker status; (1) If the participant is found to be AR-V7 positive, then non-hormonal treatment is recommended (docetaxel chemotherapy, cabazitaxel chemotherapy or radium-223 therapy) or (2) if the participant is found to be AR-V7 negative, then next generation hormonal treatment is recommended (either enzalutamide or abiraterone).

The results of the AR-V7 biomarker assessment will be provided securely to the clinical team to enable tailored treatments based on AR-V7 expression from the biomarker result. Where a decision is made that the participant will receive a non recommended therapy (either by the clinician or patient), this therapy, and the reasons for giving this will be documented.

**Control arm**

Participants with their clinical care team will make an informed and preference-based decision to receive standard care, including either next generation hormone treatments abiraterone or enzalutamide or non-hormonal approaches including docetaxel or cabazitaxel chemotherapy or radium-223. Details of all treatment administered, including doses, will be recorded as part of the trial.

The research team at sites will not receive the participants AR-V7 biomarker results.

**Outcome Measures**

Standardised clinical assessment tools used in monitoring CRPC disease and progression on treatment will be reported (listed in box 1). Primary outcome measures are related to feasibility (recruitment, retention and adherence) and will report the following;

(1) the proportion of prostate cancer patients identified through clinics who meet the eligibility criteria;

(2) the number of patients accrued per site per month over the course of the trial;

(3) baseline prevalence of AR-V7 expression in the participant cohort (this will be presented as a crude percentage of AR-V7 positivity of total participants, and in each arm);

(4) the willingness of patients to be randomised (defined as the proportion of patients consenting to be randomised from all eligible patients approached about the study);



For peer review only - http://bmjopen.bmj.com/site/about/guidelines.xhtml

(5) compliance rate (this will be defined as the number of patients who start randomised treatment as a proportion of the number randomised);
(6) the proportion of patients who: start AR-V7 recommended treatment; start treatment other than the recommended treatment; change treatment before disease progression; or withdraw. (This measure will capture information regarding patients who choose not to take recommended treatment because of strong preferences and patients who progress rapidly while waiting for treatment with a change in eligibility for treatment options).
(7) the proportion of trial participants with assessable blood samples for biomarker status (which would affect treatment targeting);
(8) the median time from the blood sample being drawn to; (i) AR-V7 result being sent back to the site and (ii) patient starting treatment (and compared with standard of care treatment).
(9) the proportion of randomised patients for whom data is collected on each clinical and health economic outcome at baseline, 12 and 24 weeks.

| Box 1 Standardised Clinical Assessment Tools |
|---|
| Clinical Outcome Measures:<br>(1)Time to PSA progression; (i) Confirmed rising PSA more than 12 weeks after randomisation. (*Where there has been a decline in PSA from baseline, progression will be a 25% or greater increase, and an absolute increase of at least 2ng/mL, from the nadir, which is confirmed by a second value obtained 3 or more weeks later. Where no decline from baseline is documented, progression must be a 25% or greater increase from the baseline value along with an increase in absolute value of 2 ng/mL or more. In all cases, the initial rise in PSA must occur after a minimum of 12 weeks from randomisation*).<br>(2)Clinical progression and survival within 6-months; (i) Number of patients who have progressed clinically at 6-months (includes change of systemic anti-cancer therapy and death from PC); (ii) Cancer specific survival at 6-months; and (iii) overall survival at 6-months.<br>(3)Quality of life for patients with cancer (EORTC QLQ-C30).<br>(4)Additional quality of life items patients with prostate cancer (EORTC QLQ-PR25).<br>(5)Participant costs questionnaire (Use of Health Services Questionnaire). |

Further information on recruitment, screening, the patient consent procedure and informed consent literature can be found in the supplementary section.

## Data collection

Table 1 shows a trial schedule of events. A more detailed description of all data collection including a data management plan, can be found in the supplementary section. In summary, in addition to collecting standard care assessment of disease status data from patients in the intervention and control arms, trial specific questionnaire assessment (EORTC QLQ-C30 Quality of Life of Cancer Patients, EORTC QLQ-PR25 Quality of Life Prostate Cancer Module) will take place at the baseline, 12 and 24 week visit.

| Procedure | Screening | VISIT 1 Consent/ Baseline | VISIT 2 12 weeks | VISIT 3 24 weeks |
|---|---|---|---|---|



For peer review only

For peer review only - http://bmjopen.bmj.com/site/about/guidelines.xhtml

| | | | | |
|---|---|---|---|---|
| | | | **(+/- 2 weeks)** | **(+/- 2 weeks)** |
| Medical History and Demographics | X | | | |
| Record results of standard care PSA test | X | X | X | X |
| Eligibility Assessment | X[a] | X[a] | | |
| Patient Information Sheet | X | | | |
| Informed consent | | X | | |
| Testosterone if no previous confirmation | | X[b] | | |
| Confirmation of eligibility | | X[a] | | |
| Randomisation | | X | | |
| Access to standard of care haemoglobin and biochemistry results | | X | | |
| Blood sample collection and shipment for CTC/ctDNA blood assessment and AR-V7 analysis (analysed at NICR labs) | | X | X | X |
| CTC blood sample collection and shipment for cross site validation[c] (analysed at All Wales Medical Genetics Lab) | | X[c] | | |
| EORTC QLQ-C30/PR25 Questionnaires | | X | | X |
| AR-V7 blood test result feedback to patient[d] | | X[d] | | |
| Use of Health Services Questionnaire | | | | X |
| Anti-cancer therapy review | | | X | X |
| Clinical assessment of disease status | | | X | X |

Table 1. Trial Schedule of Events

a = Eligibility assessment performed against trial eligibility criteria in screening, patients likely to be eligible will be given a VARIANT Information Sheet and trial information. Eligibility

For peer review only



For peer review only - http://bmjopen.bmj.com/site/about/guidelines.xhtml

will be confirmed by an Investigator (medically qualified doctor) after patients have provided written informed consent and before randomisation.

b = in those cases where there is no previous confirmation of castrate levels of testosterone only. These patients will not be randomised until castration is confirmed and the patient is documented as eligible.

c = for selected patients only (confirmed at randomisation), for cross site validation of AR-V7 status

d = for patients randomised to the personalised standard treatment arm (guided by AR-V7 biomarker) only.

**AR-V7 biomarker measure**

A validated two-centre pipeline (consisting of preanalytical, analytical and postanalytical phases) to measure AR-V7 biomarker using the commercially available AdnaTest ProstateCancerPanel AR-V7 circulating tumour cell (CTC) quantitative RT-PCR (RT-qPCR) assay (Qiagen®) (intended for molecular biology applications), has been set up according to assay manufacturers recommendations, analytical methods and sponsor agreed SOP's. Following biomarker data analysis and data verification, for participants randomised to the intervention standard treatment arm (AR-V7 biomarker guided), the baseline biomarker result and biomarker treatment recommendation will be sent securely within 10 working days to the local PI and delegated research staff. Further information on the specifics of AR-V7 biomarker driven personalised treatment (sample receipt, processing, analysis and reporting of read-out) can be found in the supplementary section.

**Data Analysis Plan**

Analyses will be conducted on an intention-to-treat basis, with sensitivity analyses used to investigate the impact of non-compliance to allocated arm. Given the feasibility status of this study, all statistical analyses will be descriptive. The majority of the outcome data will be presented in simple descriptive tables presenting percentages, means and standard deviations or 5-number summary (as appropriate), for each arm of the study. Analysis of clinical and biomarker measures will be assessed by; (1) clinical progression and survival within 6-months; (2) PSA response/progression (confirmed rising PSA more than 12 weeks after randomisation); (3) clinical progression and survival (overall and cancer specific) within 6-months (includes change of cancer therapy for progression); and (4) survival (overall and progression free) estimates will be derived using the Kaplan Meier method and presented as 6-month rates with confidence intervals. The relationship between survival estimates and continuous AR-V7 biomarker expression will be modelled considering non-linear transformations in a univariate Cox model, or parametric alternative, presented as parameter estimates (HR) with confidence intervals.

Compliance with quality of life and health economic measures will be assessed by; (1) number of patients completing measures as a proportion of the number randomised; (2) degree of



For peer review only - http://bmjopen.bmj.com/site/about/guidelines.xhtml

For peer review only

completeness of each domain of the EORTC QoL and economic questionnaire measures. These scores will be presented graphically and with numeric descriptive statistics.Data Management

**Study statistical size calculations**

This trial is designed as feasibility trial according to definition of Eldridge *et al*.(53). Feasibility includes the deliverability of the intervention and in this case, assessment of the frequency of the positive assay measurements (predicted at approximately 30%). It has been recommended that data in an external pilot trial is collected on a minimum of 60 patients per arm to estimate the ‘event’ rate(54). However, we plan to calculate a pooled estimate of overall recruitment rate, and overall biomarker prevalence rate, and will recruit 70 patients in total to allow for attrition.

The performance of potential outcome measures for a definitive trial will be assessed by estimating data completeness of the instruments and any potential bias in the completion of follow-up data. This information will be used to inform the design, choice of outcomes, necessary sample size and approach to the analysis, of a future definitive trial.

**Safety Reporting**

This is a low risk trial and no specific safety reporting is required. Should an Investigator have any concern regarding participant safety as an outcome of their participation in the trial, they will contact the Trial Management Group (TMG) and Chief Investigator as soon as possible. The Trial Oversight Committee (TOC) will monitor concerns as required.

**Trial Conduct and Governance**

The trial will be conducted in accordance with the UK Policy Framework for Health and Social Care and, as applicable, the Guidelines for Good Clinical Practice. The TMG is responsible for the day-to-day management of the trial, overseeing all aspects of the conduct of trial to ensure that the protocol is adhered to and take appropriate actions to ensure patient and data safety. The TOC will review trial conduct and accumulating clinical trial data and provide overall supervision for the trial on behalf of the Sponsor and the Funder. The constitution of the TMG and TOC including roles and responsibilities delegation for this trial can be found in the supplementary section. Aggregated data will be analysed by the Trial Statisticians and reported to an external independent TOC at least annually.

**Public and Patient Involvement**

The design, planning and management of this trial has been supported by two prostate cancer patient representatives (co-applicant on the funding grant and TMG member), both have advocated the dissemination of trial findings to patients and ensured that the public was adequately considered during trial design. PPI has been embedded into the study, with the patient’s voice a strong theme to inform and influence the on-going research and development of participant information resources in collaboration with the ‘Cancer Perspectives’ patient representative group (Newcastle upon Tyne Hospitals NHS Foundation Trust). A strong



For peer review only - http://bmjopen.bmj.com/site/about/guidelines.xhtml

commitment is to inform the participants of the outcome of this project, a clear new gold standard for PPI.

## Ethics and dissemination

Favourable ethical opinion has been obtained from the Wales National Research Ethics Service (NRES) Committee 2 18/WA/0419. All parties will conduct the trial in accordance with this ethical opinion. No amendment to protocol will be made without consideration and approval by the Trial Management Committee.

Feasibility data will be published as a peer-reviewed article and if successful, these findings will contribute to gaining further funding for a HTA full trial. In addition, assessing clinical data and blood derivatives from the participant cohort will provide valuable material (circulating tumour cells CTCs, transcript & plasma ctDNA), to validate translational studies of other AR aberrations and hormone targeting resistance pathways (or the emergence of biomarkers for chemo-sensitivity), to inform and contribute further to the rapidly evolving treatment developments for CRPC. Participants will remain anonymised in all publications.

We will also utilise dissemination through patient and professional organisations that have expressed their support for this trial (PCF, CRUK, NCRI Prostate CSG and BAUS) and through media outlets including web resources, lay press, academic national and international conferences and peer-reviewed journal publication.

Trial registration number: ISRCTN10246848

## Figure legends

Figure 1 VARIANT trial consort diagram. Planned flow of participants throughout the VARIANT study. mCRPC, Metastatic Castrate Resistant Prostate Cancer; PSA, Prostate Specific Antigen; EORTC-QLQ-C30/PR25, European Organisation for Research and Treatment of Cancer Quality of Life Questionnaire; AR-V7, Androgen Receptor Variant 7.

## References

1. Cancer Research UK (CRUK). Prostate Cancer Statistics. Electronic citation. [accessed 2019 August]. Available from: https://www.cancerresearchuk.org/health-professional/cancer-statistics/statistics-by-cancer-type/prostate-cancer.

2. Wu JN, Fish KM, Evans CP, Devere White RW, Dall'Era MA. No improvement noted in overall or cause-specific survival for men presenting with metastatic prostate cancer over a 20-year period. Cancer. 2014;120(6):818-23.

3. Public Health England. Cancer Information Tools. Electronic citation. [accessed 2019 Aug]. Available from: http://www.ncin.org.uk/cancer_information_tools .

4. Feldman BJ, Feldman D. The development of androgen-independent prostate cancer. Nat Rev Cancer. 2001;1(1):34-45.



For peer review only - http://bmjopen.bmj.com/site/about/guidelines.xhtml

5. James ND, Sydes MR, Clarke NW, Mason MD, Dearnaley DP, Spears MR, et al. Addition of docetaxel, zoledronic acid, or both to first-line long-term hormone therapy in prostate cancer (STAMPEDE): survival results from an adaptive, multiarm, multistage, platform randomised controlled trial. Lancet. 2016;387(10024):1163-77

6. Kyriakopoulos CE, Chen YH, Carducci MA, Liu G, Jarrard DF, Hahn NM, et al. Chemohormonal Therapy in Metastatic Hormone-Sensitive Prostate Cancer: Long-Term Survival Analyisis of the Randomized Phase III E3805 CHAARTED Trial. J Clin Oncol. 2018;36(11):1080-87

7. James ND, de Bono JS, Spears MR, Clarke NW, Mason MD, Dearnaley DP, et al. Aberaterone for Prostate Cancer Not Previously Treated with Hormone Therapy. N Engl J Med. 2017;377(4):338-351

8. Fizazi K, Tran N, Fein L, Matsubara N, Rodriguez-Antolin A, Alekseev BY, et al. Aberaterone plus Prednisone in Metastatic, Castration-Sensitive Prostate Cancer. N Engl J Med. 2017;377(4):352-360

9. Davis ID, Martin AJ, Stockler MR, Begbie S, Chi KN, Chowdhury S, et al. Enzalutamide with Standard First-Line Therapy in Metastatic Prostate Cancer. N Engl J Med. 2019;381(2):121-31.

10. Chi KN, Agarwal N, Bjartell A, Chung BH, Pereira de Santana Gomes AJ, Given R, et al. Apalutamide for Metastatic, Castration-Sensitive Prostate Cancer. N Engl J Med. 2019;381(1):13-24.

11. Ross RW, Xie W, Regan MM, Pomerantz M, Nakabayashi M, Daskivich TJ, et al. Efficacy of androgen deprivation therapy (ADT) in patients with advanced prostate cancer: association between Gleason score, prostate-specific antigen level, and prior ADT exposure with duration of ADT effect. Cancer. 2008;112(6):1247-53.

12. Eisenberger MA, Blumenstein BA, Crawford ED, Miller G, McLeod DG, Loehrer PJ, et al. Bilateral orchiectomy with or without flutamide for metastatic prostate cancer. N Engl J Med. 1998;339(15):1036-42.

13. Crawford ED, Eisenberger MA, McLeod DG, Spaulding JT, Benson R, Dorr FA, et al. A controlled trial of leuprolide with and without flutamide in prostatic carcinoma. N Engl J Med. 1989;321(7):419-24.

14. ASCO. Practice Guidelines. https://wwwascoorg/practice-guidelines/qualityguidelines/ guidelines/genitourinary-cancer. Accessed 13/10/2016.

15. Ryan CJ, Smith MR, de Bono JS, Molina A, Logothetis CJ, de Souza P, et al. Abiraterone in metastatic prostate cancer without previous chemotherapy. N Engl J Med. 2013;368(2):138-48.

16. Scher HI, Fizazi K, Saad F, Taplin ME, Sternberg CN, Miller K, et al. Increased survival with enzalutamide in prostate cancer after chemotherapy. N Engl J Med. 2012;367(13):1187-97.



For peer review only - http://bmjopen.bmj.com/site/about/guidelines.xhtml

17. Dehm SM, Tindall DJ. Alternatively spliced androgen receptor variants. Endocr Relat Cancer. 2011;18(5):R183-96.

18. Luo J. Development of AR-V7 as a putative treatment selection marker for metastatic castration-resistant prostate cancer. Asian J Androl. 2016;18(4):580-5.

19. Ware KE, Garcia-Blanco MA, Armstrong AJ, Dehm SM. Biologic and clinical significance of androgen receptor variants in castration resistant prostate cancer. Endocr Relat Cancer. 2014;21(4):T87-T103.

20. Beltran H, Antonarakis ES, Morris MJ, Attard G. Emerging Molecular Biomarkers in Advanced Prostate Cancer: Translation to the Clinic. Am Soc Clin Oncol Educ Book. 2016;35:131-41.

21. Maughan BL, Antonarakis ES. Clinical Relevance of Androgen Receptor Splice Variants in Castration-Resistant Prostate Cancer. Curr Treat Options Oncol. 2015;16(12):57.

22. Daniel M, Dehm SM. Lessons from tissue compartment-specific analysis of androgen receptor alterations in prostate cancer. J Steroid Biochem Mol Biol. 2016.

23. Stone L. Prostate cancer: AR-V7 status in CTCs is a treatment-specific biomarker. Nat Rev Urol. 2016;13(8):433.

24. Wadosky KM, Koochekpour S. Molecular mechanisms underlying resistance to androgen deprivation therapy in prostate cancer. Oncotarget. 2016.

25. Nakazawa M, Lu C, Chen Y, Paller CJ, Carducci MA, Eisenberger MA, et al. Serial blood-based analysis of AR-V7 in men with advanced prostate cancer. Ann Oncol. 2015;26(9):1859-65.

26. Steinestel J, Luedeke M, Arndt A, Schnoeller TJ, Lennerz JK, Wurm C, et al. Detecting predictive androgen receptor modifications in circulating prostate cancer cells. Oncotarget. 2015.

27. Scher HI, Lu D, Schreiber NA, Louw J, Graf RP, Vargas HA, et al. Association of AR-V7 on Circulating Tumor Cells as a Treatment-Specific Biomarker With Outcomes and Survival in Castration-Resistant Prostate Cancer. JAMA Oncol. 2016.

28. Hu R, Dunn TA, Wei S, Isharwal S, Veltri RW, Humphreys E, et al. Ligand-independent androgen receptor variants derived from splicing of cryptic exons signify hormone-refractory prostate cancer. Cancer Res. 2009;69(1):16-22.

29. Antonarakis ES, Lu C, Wang H, Luber B, Nakazawa M, Roeser JC, et al. AR-V7 and resistance
to enzalutamide and abiraterone in prostate cancer. N Engl J Med. 2014;371(11):1028-38.



For peer review only - http://bmjopen.bmj.com/site/about/guidelines.xhtml

30. Antonarakis ES, Lu C, Luber B, Wang H, Chen Y, Nakazawa M, et al. Androgen Receptor Splice Variant 7 and Efficacy of Taxane Chemotherapy in Patients With Metastatic Castration-Resistant Prostate Cancer. JAMA Oncol. 2015;1(5):582-91.

31. Armstrong AJ, Halabi S, Luo J, Nanus DM, Giannakakou P, Szmulewitz RZ, et al. Prospective Multicenter Validation of Androgen Receptor Splice Variant 7 and Hormone Therapy Resistance in High-Risk Castration-Resistant Prostate Cancer: The PROPHECY Study. J Clin Oncol. 2019;37(13):1120-9.

32. Febbo PG, Ladanyi M, Aldape KD, De Marzo AM, Hammond ME, Hayes DF, et al. NCCN Task Force report: Evaluating the clinical utility of tumor markers in oncology. J Natl Compr Canc Netw.2011;9 Suppl 5:S1-32; quiz S3.

33. O'Connor JP, Aboagye EO, Adams JE, Aerts HJ, Barrington SF, Beer AJ, et al. Imaging biomarker roadmap for cancer studies. Nat Rev Clin Oncol. 2017;14(3):169-86.

34. Hornberg E, Ylitalo EB, Crnalic S, Antti H, Stattin P, Widmark A, et al. Expression of androgen receptor splice variants in prostate cancer bone metastases is associated with castration-resistance and short survival. PLoS One. 2011;6(4):e19059

35. Antonarakis ES. Predicting treatment response in castration-resistant prostate cancer: could androgen receptor variant-7 hold the key? Expert Rev Anticancer Ther. 2015;15(2):143-5.

36. Antonarakis ES, Luo J. Prostate cancer: AR splice variant dimerization-clinical implications. Nat Rev Urol. 2015;12(8):431-3

37. De Laere B, van Dam PJ, Whitington T, Mayrhofer M, Diaz EH, Van den Eynden G, et al. Comprehensive Profiling of the Androgen Receptor in Liquid Biopsies from Castration-resistant Prostate Cancer Reveals Novel Intra-AR Structural Variation and Splice Variant Expression Patterns. Eur Urol. 2017;72(2):192-200

38. Sieuwerts AM, Mostert B, van der Vlugt-Daane M, Kraan J, Beaufort CM, Van M, et al. An In-Depth Evaluation of the Validity and Logistics Surrounding the Testing of AR-V7 mRNA Expression in Circulating Tumor Cells. J Mol Diagn. 2018;20(3):316-25.

39. Hayes DF, Bast RC, Desch CE, Fritsche H, Jr., Kemeny NE, Jessup JM, et al. Tumor marker
utility grading system: a framework to evaluate clinical utility of tumor markers. J Natl Cancer Inst. 1996;88(20):1456-66.

40. Simon RM, Paik S, Hayes DF. Use of archived specimens in evaluation of prognostic and predictive biomarkers. J Natl Cancer Inst. 2009;101(21):1446-52.

41. Todenhofer T, Hennenlotter J, Feyerabend S, Aufderklamm S, Mischinger J, Kuhs U, et al. Preliminary experience on the use of the Adnatest(R) system for detection of circulating tumor cells in prostate cancer patients. Anticancer Res. 2012;32(8):3507-13.

42. Attard G, Borre M, Gurney H, Loriot Y, Andresen C, Kalleda R, et al. ASCO 2017: Clinical factors associated with AR-V7 detection in ARMOR3-SV, a randomized trial of galeterone vs



For peer review only - http://bmjopen.bmj.com/site/about/guidelines.xhtml

enzalutamide in men with AR-V7+ metastatic castration-resistant prostate cancer. Abstract 2017 ASCO Annual Meeting. June 2-6, 2017, Chicago.

43. Taplin ME, Antonarakis ES, Ferrante KJ, Horgan K, Blumenstein BA, Saad F, et al. Clinical factors associated with AR-V7 detection in ARMOR3-SV, a randomized trial of galeterone (Gal) vs enzalutamide (Enz) in men with AR-V7+ metastatic castration-resistant prostate cancer (mCRPC). J Clin Oncol. 2017;35:15_suppl, 5005-5005

44. Antonarakis ES, Lu C, Luber B, Wang H, Chen Y, Zhu Y, et al. Clinical Significance of Androgen Receptor Splice Variant-7 mRNA Detection in Circulating Tumor Cells of Men with Metastatic Castration-Resistance Prostate Cancer Treated with First- and Second-Line Abiraterone and Enzalutamide. J Clin Oncol. 2017;35(19):2149-56

45. Markowski MC, Frick KD, Eshleman JR, Luo J, Antonarakis ES. Cost-savings analysis of AR-V7 testing in patients with metastatic castration-resistant prostate cancer eligible for treatment with abiraterone or enzalutamide. Prostate. 2016.

46. Sharp A, Porta N, Lambros MBK, Welti JC, Paschalis A, Raj GV, et al. Dissecting Prognostic From Predictive Utility: Circulating AR-V7 Biomarker Testing for Advanced Prostate Cancer. J Clin Oncol. 2019;37(24):2182-4.

47. De Laere B, Ost P, Gronberg H, Lindberg J. Has the PROPHECY of AR-V7 Been Fulfilled? J Clin Oncol. 2019;37(24):2181-2.

48. Dirix L. Predictive Significance of Androgen Receptor Splice Variant 7 in Patients With Metastatic Castration-Resistant Prostate Cancer: The PROPHECY Study. J Clin Oncol. 2019;37(24):2180-1.
49. Armstrong AJ, Halabi S, Luo J, Nanus DM, Scher HI, Antonarakis ES, et al. Reply to L. Dirix, B. De Laere et al, and A. Sharp et al. J Clin Oncol. 2019;37(24):2184-6

50. Van Etten JL, Nyguist M, Li Y, Yang R, Ho Y, Johnson R, et al. Targeting a Single Alternative Polyadenylation Site Coordinately Blocks Expression of Androgen Receptor mRNA Splice Variants in Prostate Cancer. Cancer Res. 2017;77(19):5228-35

51. Henzler C, Li Y, Yang R, McBride T, Ho Y, Sprenger C, et al. Truncation and constitutive activation of the androgen receptor by diverse genomic rearrangements in prostate cancer. Nat Commun. 2016;7:13668

52. James ND, Spears MR, Clarke NW, Dearnaley DP, De Bono JS, Gale J, et al. Survival with Newly Diagnosed Metastatic Prostate Cancer in the "Docetaxel Era": Data from 917 Patients in the Control Arm of the STAMPEDE Trial (MRC PR08, CRUK/06/019). Eur Urol. 2015;67(6):1028-38

53. Eldridge SM, Lancaster GA, Campbell MJ, Thabane L, Hopewell S, Coleman CL, et al. Defining Feasibility and Pilot Studies in Preparation for Randomised Controlled Trials: Development of a Conceptual Framework. PLoS One. 2016;11(3):e0150205.

54. Teare MD, Dimairo M, Shephard N, Hayman A, Whitehead A, Walters SJ. Sample size



For peer review only - http://bmjopen.bmj.com/site/about/guidelines.xhtml

requirements to estimate key design parameters from external pilot randomised controlled trials: a simulation study. Trials. 2014;15:264.

55. Zhang J, Cunnigham JJ, Brown JS and Gatenby RA. Integrating evolutionary dynamics into treatment of metastatic castrate-resistant prostate cancer. Nat Commun. 2017;8(1):1861

## Acknowledgments

We would like to acknowledge the valuable support of the VARIANT Trial Oversight Committee (TOC) members Dr Alison Tree, Dr Andrew Feber, Dr Rhian Gabe and our patient representatives on this committee Robin Millman and Paul Nash. We are grateful for the assistance and support of our scientific collaborators Scott Dehm (University of Minnesota), Luke Gaughan (NICR), Jun Luo (John Hopkins), Gert Attard (UCL) and the All Wales Medical Genetics Laboratory (AWMGL).

Management of the study is by Newcastle University Clinical Trial Unit (NCTU).

## Authors' contribution

Emma Clark: Conception and design of the work, data collection, drafting of the article, critical revision of the article and final approval of the version to be published

Miranda Morton: Trial management, data collection and drafting of the article.

Shriya Sharma: Trial management, data collection and drafting of the article.

Holly Fisher: Conception and design of the work, drafting of the article and critical revision of the article.

Denise Howel: Data collection, drafting of the article, critical revision of the article and final approval of the version to be published.

Jenn Walker: Trial management and data collection.

Ruth Wood: Trial management, data collection, drafting of the article data collection, critical revision of the article and final approval of the version to be published

Helen Hancock:Ccritical revision of the article and final approval of the version to be published.

Rebecca Maier: Critical revision of the article.

John Marshall: Conception and design of the work, critical revision of the article and final approval of the version to be published.

Amit Bahl: conception and design of the work.

Simon Crabb: conception and design of the work and critical revision of the article.

Suneil Jain: conception and design of the work and critical revision of the article.

Ian Pedley: conception and design of the work, data collection, critical revision of the article and final approval of the version to be published



For peer review only - http://bmjopen.bmj.com/site/about/guidelines.xhtml

696 Rob Jones: conception and design of the work, data collection, drafting of the article, critical revision of the article and final approval of the version to be published

698 John Staffurth: conception and design of the work, data collection, drafting of the article, critical revision of the article and final approval of the version to be published

700 Rakesh Heer: conception and design of the work, drafting of the article, critical revision of the article and final approval of the version to be published

## Funding statement

This work was supported by a National Institute for Health Research (NIHR) Research for Patient Benefit (grant number PB-PG-0816-20043). The PHOTO trial is sponsored by Newcastle upon Tyne Hospitals National Health Service Foundation Trust.

## Competing Interests Statement

JS reports non-financial support from Bayer and personal fees from Janssen and Astellas outside of the submitted work. SC has an honoraria/advisory role with Roche, Clovis Oncology, Bayer, Janssen Cilag and Merck and receives research support from AstraZeneca, Astex Pharmaceuticals and Clovis Oncology.

**Word Count (excluding title page, tables and reference list): 4, 364**



For peer review only - http://bmjopen.bmj.com/site/about/guidelines.xhtml

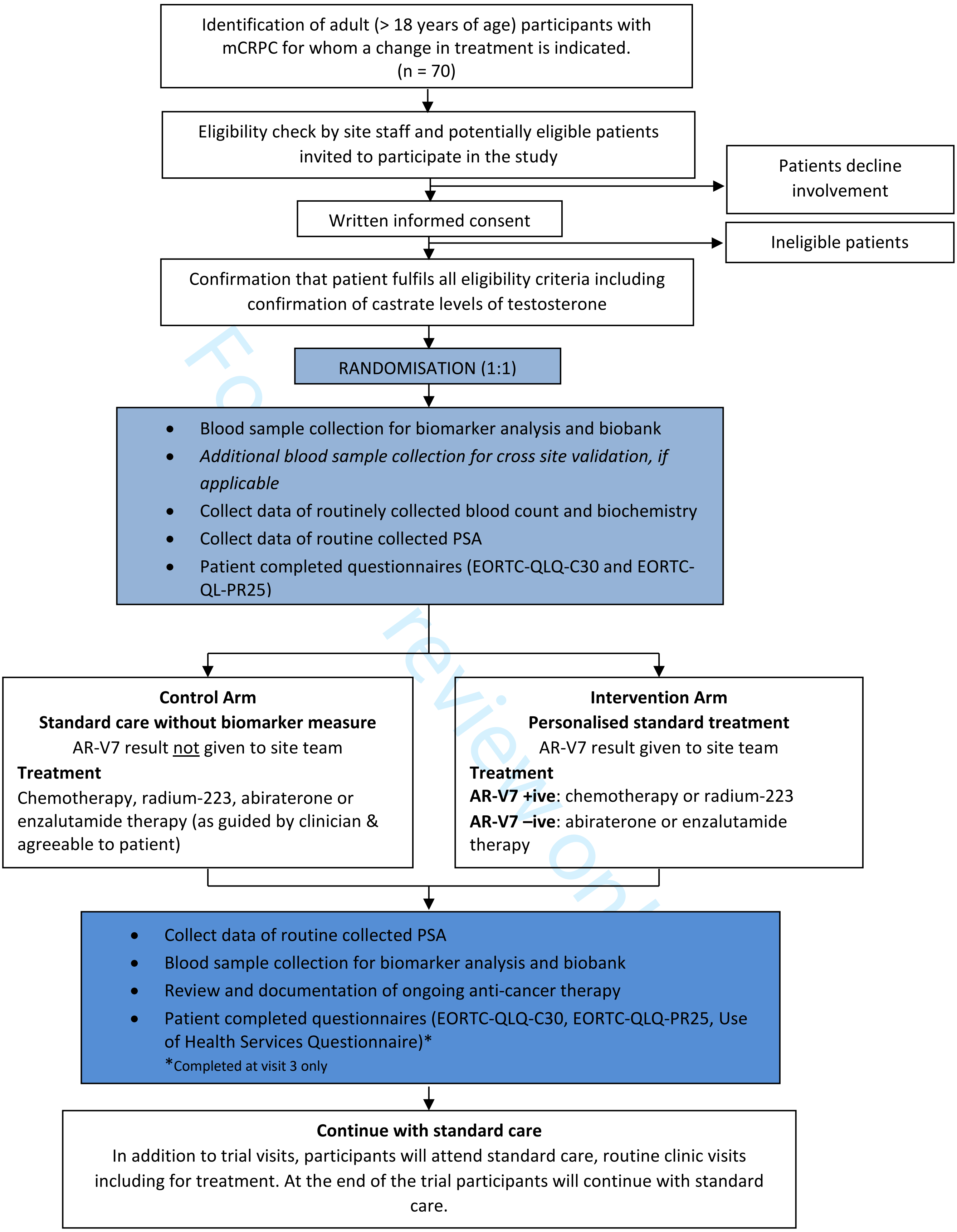


**Figure 1** VARIANT trial consort diagram. Planned flow of participants throughout the VARIANT study. mCRPC, Metastatic Castrate Resistant Prostate Cancer; PSA, Prostate Specific Antigen; EORTC-QLQ-C30/PR25, European Organisation for Research and Treatment of Cancer Quality of Life Questionnaire; AR-V7, Androgen Receptor Variant 7.

For peer review only - http://bmjopen.bmj.com/site/about/guidelines.xhtml

Supplementary Data – VARIANT Protocol

The constitution of the Trial Management Group (TMG): Chief Investigator, Sponsor representative, Co-Lead Investigator, Laboratory and Translational Science Lead, Statistician Lead, Trial Management Team, Co-Applicants and Collaborators who will attend TMG meetings as required.

The constitution of the Trial Oversight Committee (TOC) as a combined TOC whose members are independent of the trial: independent Chair, Independent Lab representative, Independent statistician and two independent patient representatives.

**Roles and Responsibilities**

Chief Investigator - Dr Rakesh Heer, Senior Lecturer and Consultant Urological Surgeon, Northern Institute for Cancer Research, Newcastle University

Co-Lead Investigator - Prof. John Staffurth, Professor in Oncology and Consultant Oncologist, Velindre Cancer Centre, Cardiff University

Laboratory and Translational Science Lead - Dr Emma Clark, Translational Research Associate, Northern Institute for Cancer Research, Newcastle University

Principle Investigator - Prof Rob Jones, Professor of Clinical Cancer Research/ Honorary Consultant in Medical Oncology, University of Glasgow

Principle Investigator - Dr Ian Pedley, Clinical Director of NCCC and Clinical Oncologist, The Newcastle upon Tyne Hospitals NHS Foundation Trust

Co-applicants – (1) Dr Amit Bahl, Senior Lecturer, Consultant Oncologist and Clinical Director, University Hospitals Bristol NHS Foundation Trust; (2) Dr Simon Crabb, Associate Professor and Honorary Consultant in Medical Oncology, University of Southampton; (3) Dr Suneil Jain, Senior Lecturer and Consultant in Clinical Oncology, Queen's University Belfast.

PPI Representative - Dr John Marshall

Senior Statistician - Denise Howel, Institute of Health and Society, Newcastle University

Statistician - Dr Holly Fisher, Institute of Health and Society, Newcastle University

Data Manager – Ruth Wood, Newcastle Clinical Trials Unit, Newcastle University

Senior Trial Manager – Jenn Walker, Newcastle Clinical Trials Unit, Newcastle University

Joint Trial Manager - Dr Miranda Morton and Shriya Sharma, Newcastle Clinical Trials Unit, Newcastle University

Sponsor - The Newcastle upon Tyne Hospitals NHS Foundation Trust

Funder - National Institute for Health Research – Research for Patient Benefit



For peer review only - http://bmjopen.bmj.com/site/about/guidelines.xhtml

Out of Hours Contact - Dr Rakesh Heer, Newcastle University

Trial Oversight Committee (TOC) Chair - Dr Alison Tree, Uro-oncology Trials Team Leader and Consultant Clinical Oncologist, The Institute of Cancer Research

## Recruitment and screening

Patients will be approached during routine clinic appointments from urology or oncology clinical services. Potentially eligible patients will have the trial explained to them, provided with a Patient Information Sheet (PIS) and their medical notes reviewed to establish if they are likely to be eligible to take part in the trial. In addition to assessing patient eligibility against the inclusion and exclusion criteria, a complete medical history including the patient's age and detailed information about their prostate cancer history and metastases will be collected after consent.

## Consent Procedure

Full written informed consent will be received by signing, dating and initialling the consent form, which will be witnessed by a member of the research team, who has documented and delegated responsibility, and will and check eligibility and counter-sign the consent form. The participant will specifically consent to; (1) their GP being contacted and informed of participation in the study; (2) access to relevant sections of their medical notes to carry out follow-up after the trial has ended; and (3) serial collection of blood samples for biomarker testing and storage in the Androgen Receptor Biology Bio-Bank (AR-3B) biobank for up to 10 years after the trial has ended.

## Data collection methods

In addition to collecting standard care assessment of disease status data from intervention and control groups, trial specific questionnaire assessment (EORTC QLQ-C30 Quality of Life of Cancer Patients, EORTC QLQ-PR25 Quality of Life Prostate Cancer Module) and blood sample collection will take place at baseline, 12 and 24 week visits. Use of Health Services Questionnaire will be completed end of trial assessments. Participant data will only be identified using a unique individual participant identifier.

### Standard care assessments:

The following clinical assessments will be conducted;

1. Cause of death (if appropriate)
2. Evidence of PSA progression (>25% and >2 ng/mL above the nadir and confirmed by a second value >3 weeks later)
3. Clinical evidence of progression, stage and type of progression (biochemical, radiological or symptomatic)
4. Result of routinely collected PSA and testosterone measurements, full blood count and biochemistry tests



For peer review only - http://bmjopen.bmj.com/site/about/guidelines.xhtml

5. Details of anti-cancer therapy, including dates of treatments for ongoing anti-cancer therapy.

**Questionnaires:**

The EORTC quality of life questionnaire is an integrated system for assessing the health-related quality of life (QOL) of cancer patients participating in clinical trials. There is a set of core questions (QLQ-C30), supplemented by a prostate cancer specific module (PR25). PR25 is a diagnosis-specific module designed to be used in conjunction with the QLQ-C30, it is intended for use among a wide range of patients with prostate cancer varying in disease stage and treatment modality.

The QLQ-C30 consists of thirty questions incorporating; (1) functional scales, symptom scales and a number of items assessing additional symptoms commonly reported by patients with cancer, assessed on a four point scale and (2) a global health status/quality of life scale, assessed on a seven point scale. The PR25 module consists of 25 questions incorporating functional and symptom scales, all assessed on four point scale. The Use of Health Services questionnaire consists of ten questions assessing participant's use of health services over the course of their participation in the trial.

## Data Management and Archiving

Data including the number of patients screened, approached and interested in taking part will be collected via a screening log. Trial and screening data is collected on electronic case report forms (eCRFs) using password limited access, secure web-based interface for data entry with inbuilt back-up facility, will be managed using a Clinical Data Management System (Elsevier's MACRO™) overseen by the Newcastle Clinical Trials Unit (NCTU). Individual access will be limited according to delegated roles and duties. Data will be handled, computerised and stored in accordance with the UK Data Protection Act 2018. All trial data will be retained in accordance with the latest Directive on GCP (2005/28/EC).

Participant clinical information will not be released without the written permission of the participant, except as necessary for monitoring and auditing by the Sponsor, its designee, Regulatory Authorities, the Trial oversight committee (TOC) or the Research Ethics Committee (REC). Trial data will be released to the trial statistician for analysis, to NICR biobank researchers after trial analysis and used in planning any future, definitive trial. All trial data will be stored for 10 years, in accordance with GCP and sponsor approved SOPs.

## AR-V7 biomarker driven personalised treatment pipeline

Biomarker driven personalised treatment will be based on current scientific evidence from the Adnatest assay biomarker founding lab (John Hopkins, Baltimore, USA). A biomarker positive result is when a read is detected at 35 qPCR cycles or less and the recommendation will be to proceed with chemotherapy. A biomarker negative result is when a read is detected over 35



For peer review only - http://bmjopen.bmj.com/site/about/guidelines.xhtml

qPCR cycles (or there is no read at any qPCR cycle), and the recommendation will be to proceed with hormonal therapy (Enzalutamide or Abiraterone).

Participants will be required to give 30ml of blood at baseline (0 weeks), 12 weeks and 24 weeks. Samples will be routinely processed for biomarker read-out and AR-3B biobank storage at the Northern Institute for Cancer Research (NICR) central analysis lab as detailed in the VARIANT biomarker flow chart below. Following biomarker data analysis and data verification (according to sponsor agreed analytical and validation plan and SOPs), for participants randomised to the intervention standard treatment arm (AR-V7 biomarker guided), the baseline biomarker result and biomarker treatment recommendation will be sent securely within 10 working days to the local PI and delegated research staff. The PI and local trial team will then communicate with the participant regarding the biomarker guided treatment. Any modifications to the recommended treatment for participants will be made at the discretion of the treating clinician, based on their clinical judgement. Biomarker results from blood samples taken at weeks 12 and 24 will not be made available to the PI, local trial team or participant.

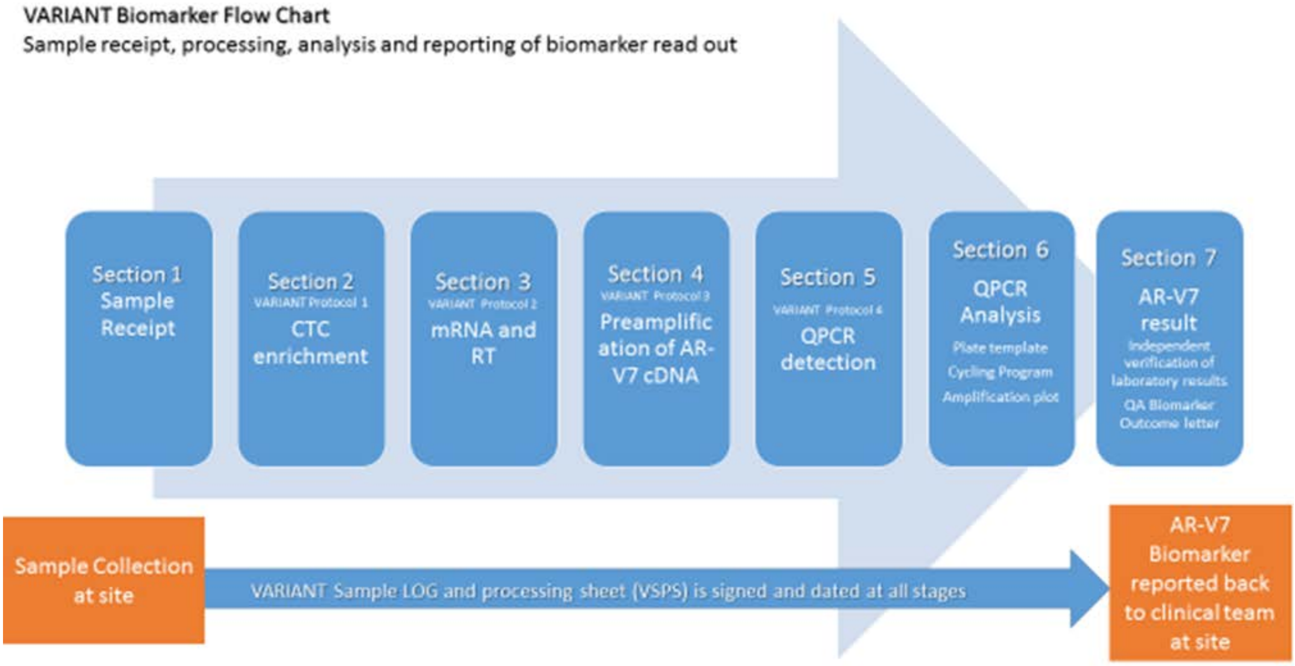


For participants randomised to the standard treatment arm (not AR-V7 biomarker guided), biomarker results will not be made available to the PI, local trial team or participant for any of the blood samples analysed (baseline, week 12 and week 24).

**AR-V7 biomarker cross-site validation**

An additional twenty participants selected at randomisation will provide a further (10ml) blood sample for cross site validation analysis of the biomarker at All Wales Medical Genetics Laboratory (AWMGL) using the commercially available AdnaTest ProstateCancerPanel AR-V7 circulating tumour cell (CTC) quantitative RT-PCR (RT-qPCR) assay (Qiagen®).

**Androgen Receptor Biology driving hormone resistance**



For peer review only - http://bmjopen.bmj.com/site/about/guidelines.xhtml

Research suggests that key 'sub-populations' or clones of molecular alterations (of which AR-V7 is an important subtype), compete with each other and are drivers of treatment resistance. In addition to performing AR-V7 biomarker assay, a fuller capture of AR-related CRPC biology will be achieved by collecting CTCs, plasma, buffy coat and red blood cell derivatives to compile an Androgen Receptor Biology Bio-Bank (AR-3B). Whole and CTC depleted blood sample derivatives will be collected and biobanked at 12 weekly intervals from baseline (prior to treatment) throughout follow up (to a maximum of 24 weeks +/- 2 weeks), including all trial captured data.

It is also important to acknowledge the expression of other AR splice variants are also associated with resistance to hormonal treatment(50,51), as are other AR pathway alterations such as AR mutations and amplifications(37). Ongoing discussions in the field as to the discriminatory value of solely detecting AR-V7 expression, may ultimately lead to a combination test that will improve treatment stratification and patient outcomes. However, at this time, AR-V7 remains the forerunner and a formal understanding of biomarker characteristics in the advanced prostate cancer treatment setting is required 'to best inform' a formal large-scale testing.

The AR-3B biorepository resource will be used to assess total VARIANT expression (capturing all VARIANTs in a single qPCR reaction) and explore (but not be limited to) AR hot spot mutations/sequencing/amplification and other mutations such as PTEN/p53/MYC gain/RB1 loss/MET gain/PARPi and further molecular pathways based on yet to be defined but new emerging data in time, in blood and blood derivatives.

This resource will provide added value to the feasibility study, by banking processed biomarker tissue for additional biomarker measures that may also contribute to hormone targeting resistance (or the emergence of biomarkers for chemo-sensitivity), and importantly be relevant in prostate cancer management. Blood samples will be transported, stored, accessed and processed in accordance with sponsor approved SOPs following appropriate legislation relating to the use and storage of human tissue for research purposes and such activities shall at least meet the requirements as set out in the 2004 Human Tissue Act and 2006 Human Tissue (Scotland) Act.

The results of any other research utilising blood samples (including those in the control arm), will not be reported back to the clinical team or to participants, this data is for research purposes only and will be published in appropriate peer reviewed scientific journals. Participants will remain anonymised in all publications.

**Androgen Receptor Biology Bio-Bank (AR-3B) sample storage governance**

Samples will be appropriately labelled in accordance with trial protocol as described in the current VARIANT trial site blood collection manual (Version 1.0 28th March 2019) to comply with the General Data Protection Regulation (GDPR) and Data Protection Act (GPA) 2018 and pseudo-anonymised with linkage to participant details possible only through the Participant Identification Log assessable only to delegated personnel. Sample shipment will be tracked and



For peer review only - http://bmjopen.bmj.com/site/about/guidelines.xhtml

sample receipt at central analysis labs recorded using a specific sample collection MACRO™ database which is separate to the main trial database.

All AR-3B biobank samples are stored under HTA license 12534, under the Designated Individual, Dr. Christopher Morris (NICR). All samples are held under the custodianship of Mr. Rakesh Heer (Chief Investigator), who is responsible for the curation of these samples and ensuring compliance with the Human Tissue Act (2004) and GCP. Samples will be tracked and stored using Achiever medical LIMS.

Blood samples sent to AWMGL will be consumed within 7 days of receipt and are not under the remit of the HTA. All RNA and DNA derivatives will be transferred to the NICR AR-3B biorepository for storage at the end of the trial.

For peer review only



For peer review only - http://bmjopen.bmj.com/site/about/guidelines.xhtml

To be printed on local headed paper

# PATIENT INFORMATION SHEET

# The Variant 7 Biomarker Feasibility Study

## The **VARIANT** Study

### We are inviting you to take part in a research study

Please read the following information to help you decide if you want to take part. It will explain why we are doing this research and what it might mean for you. You are free to decide whether or not to take part in this study. You do not have to decide straight away and you can talk to your friends/family about it. Ask us if you have any questions or you want to know more. If you choose not to take part, this will not affect the care you get from your doctors.

### Study Summary

- In this study we will be taking 3 blood samples and asking patients to complete some questionnaires during their usual hospital appointments.
- Patients with **advanced metastatic prostate cancer** (cancer that has spread from the prostate to other parts of the body), often eventually stop responding to initial hormone therapy. After this point, treatment is usually with either:
    1) **Further hormonal treatment called next generation hormone treatment OR**
    2) **Non-hormonal treatment such as chemotherapy or radiotherapy**
- There is currently no clear guidance for which of these two different standard care treatment options a patient should receive.
- Testing the level of AR-V7 (a type of protein) in blood could suggest which of these two treatments patients will respond to best. The AR-V7 test is known as a biomarker test.
- VARIANT is looking to find out if the AR-V7 biomarker test is helpful to support doctors and patients in choosing between these treatment options.
- In this study, half of patients will receive treatment guided by the AR-V7 biomarker test. The other half will receive treatment as usual.

**Please read the following information to see if you may be interested in taking part.**



For peer review only - http://bmjopen.bmj.com/site/about/guidelines.xhtml

## What is a biomarker test?

Biomarkers are substances that can be found and measured in parts of the body, in this case, the blood. Biomarker testing is a type of test that looks for these substances to give doctors information about a patient's health. **The AR-V7 blood test is a biomarker test looking for the AR-V7 protein.**

## Why is VARIANT needed?

Initial treatments for advanced metastatic prostate cancer include hormone treatment alone or chemotherapy in combination with hormone treatment. Eventually most patients with advanced prostate cancer stop responding to initial hormone treatment. At this point, patients usually receive treatment with either further hormone treatment, known as next generation hormone treatment (such as abiraterone or enzalutamide), or with non-hormonal options (typically chemotherapy, or in some cases radium-223 radiotherapy). Although there are fewer side effects associated with next generation hormone treatment relative to chemotherapy, only 30-50% of patients will respond well to further hormone treatment.

The **AR-V7 biomarker is found in the blood** of some men who have received initial hormone treatment for prostate cancer. Recent studies have suggested that patients who have this biomarker in their blood may be less likely to respond well to advanced hormone therapy. Measuring the amount of this biomarker in blood (which is not usually tested for), may be useful to help guide choice of treatment for patients with advanced metastatic prostate cancer. We hope this will improve patient experience and outcome by spending less time on and experiencing side effects of treatments that might not work, starting a different treatment earlier, and potentially reduce the cost to the NHS.

The VARIANT study is a feasibility study in which we will look at whether doctors and patients are willing to use the results of this blood test to decide on a treatment option. At this stage we do not know that the AR-V7 biomarker will lead to patients having better responses to treatments. However, we hope that the results of VARIANT will help us to plan a similar, but larger, study to find out if the AR-V7 blood test does result in better outcomes for patients and whether AR-V7 testing should be used in standard NHS practice. We hope that 70 patients will take part in VARIANT.

For peer review only

## Why have I been invited to take part?

You have been diagnosed with advanced metastatic prostate cancer and have already been treated with hormone therapy (also known as androgen deprivation therapy, or ADT). Your disease has stopped responding to the current therapy and you are due a change in treatment plan.

## Do I have to take part?

No, it is up to you to decide if you want to take part in VARIANT. If you do not want to take part, you will still get the standard treatment that has been arranged by your treating doctor.

For peer review only - http://bmjopen.bmj.com/site/about/guidelines.xhtml

If you agree to take part, you can change your mind and withdraw from the study at any time without having to give a reason.

## What does taking part involve?

- If you decide to take part, you will be selected to have either:
  - **a) treatment guided by AR-V7 blood test result**

    In this group, your treating doctor will receive the results of your AR-V7 blood test. Your treating doctor will tell you the result of the AR-V7 blood test and discuss this with you before arranging a treatment option for you. The results of the test may support you and your doctor in deciding whether next generation hormonal therapy or non-hormonal therapy would be more suitable for you.
  - **b) treatment as usual**

    In this group, your doctor will arrange a treatment option with you as they usually would if you were not in the study. You and your treating doctor will NOT receive the results of your AR-V7 blood test. The results will only be looked at by the trial management team at the end of the study.
- You will have equal chance of being in group a) or group b) (a 50:50 chance). Your group will be selected by a computer. We call this 'randomisation'. Your doctor will not have any say over which group you are in.
- It is important to note that we are not testing a new treatment in this study. All VARIANT patients will receive one of the usual treatment options available to patients in the NHS. The study is looking at whether doctors and patients are willing to use the AR-V7 blood test and whether it is helpful in deciding **which** treatment option is most suitable for individual patients.

## What will I have to do?

A member of the VARIANT team will discuss the study with you and answer any questions you may have. If you decide to take part, and your doctor confirms you are eligible for the study, you will be asked to sign a consent form.

As well as receiving the treatment arranged with your doctor, taking part in the study will mean (for ALL patients, that is patients in group a) and group b)):

- You will be asked to give a **blood sample on 3 occasions** (at the start of the study, after 12 weeks and 24 weeks). We would like to collect around 20 ml of blood (about 4 teaspoons full) each time.
- Some patients will be selected to give an **additional blood samples on one occasion** (at the start of the study). We would like to collect around 10 ml of blood (about 2 teaspoons full) for this sample. Patients will be randomly selected by a computer system to give this additional blood samples. The doctor or a member of their team will tell you if you have been selected to give this sample.



For peer review only - http://bmjopen.bmj.com/site/about/guidelines.xhtml

For peer review only

- On 2 of these visits, you will be asked to complete 2 **short questionnaires** about your quality of life. These will take around 15 minutes each to complete.
- Study visits will all take place during your usual hospital visits. There should be no extra visits to hospital required.
- We will use some information that is already collected about you as part of your standard clinical care. This includes information about your diagnosis, your treatment, results of scans and blood tests and your physical health. Taking part in VARIANT does not involve any extra scans to those you would receive normally.
- To take part in VARIANT, you will be asked to give your permission for your blood samples collected during this study to be stored at the Northern Institute of Cancer Research and used for future research as described below.

## Study timeline in addition to standard treatment

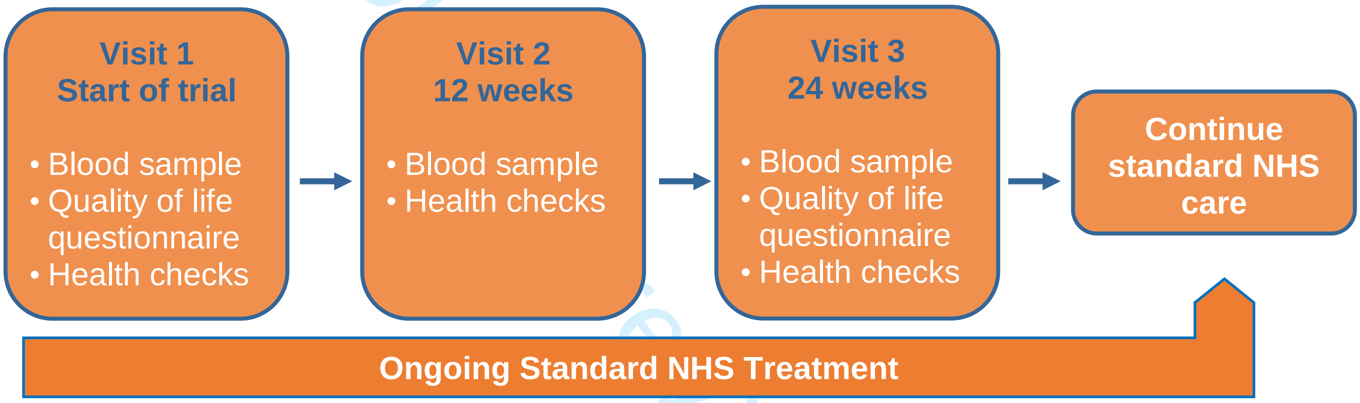


## What will happen to my blood samples?

Your blood samples will be tested for the AR-V7 biomarker at the Northern Institute for Cancer Research (NICR). If you are selected to give the additional blood sample this will be sent to the All Wales Medical Genetics Laboratory (AWMGL) for testing. You and your treating doctor will only find out the AR-V7 biomarker result if you are selected to have your treatment guided by the AR-V7 blood test result.

After the AR-V7 test, any remaining sample will be stored at the NICR biobank (a collection of samples) and will be used in future research to look at why people develop advanced prostate cancer and how cancer reacts to treatments. If you do not want your samples to be stored or used through the NICR biobank you will not be able to take part in this trial.

Your samples will be labelled with a unique study number. This number is used to link your sample back to you and only the study team and authorised people will be able to do this. Your sample will be sent with a form that includes your initials, date of birth and the date the sample was taken. This will help us make sure the samples can be linked back to the correct person.

For more detailed information about what will happen with your blood samples please see the section 'Further information about what will happen to blood samples' on page X, we recommend that you read this.


For peer review only - http://bmjopen.bmj.com/site/about/guidelines.xhtml

## Expenses and payments

You will not receive payment for taking part in VARIANT. All parts of the study will happen when you are already coming to hospital for your usual visits.

## What happens when the research study stops?

Your appointment at 24 weeks will be the last time we see you for the study. At the end of the study you will continue to receive usual clinical care as decided by your hospital and doctor. If appropriate, this may include continuing the anti-cancer drugs you are receiving, or changing to another if you and your doctor believe this to be in your best interests.

## What are the possible risks and benefits of taking part?

**Risks**: Taking blood samples may cause some discomfort and minor pain, and occasionally patients can feel faint during or after. Sometimes patients will have some bruising where the blood has been taken. Only trained members of staff will perform the blood tests and every effort will be made to prevent any discomfort.

VARIANT patients will receive one of the usual treatment options available to patients in the NHS. Your doctor will be able to tell you more about possible side effects of these treatments. The risk of these treatments is the same as if you were not taking part in the study.

**Benefits**: By taking part in VARIANT, you will be helping us gather information to learn about using the AR-V7 biomarker to guide treatment for patients with advanced metastatic prostate cancer. We hope that we can improve the quality of life of patients in the future from VARIANT.

For some patients, taking part in the study will mean that your doctor receives your AR-V7 biomarker result before deciding on a treatment for you. This may help to inform which treatment would be best for you after reviewing your medical history.

If you want to find out more about taking part in research studies, you can visit the NHS Choices Website www.nhs.uk/conditions/clinical-trials/. On this website you will also find contact details for your local Patient Advice Liaison Service (PALS) office if you would like to speak to someone.

For peer review only

## Further supporting information

## What will happen if I do not want to carry on in the study?

You can withdraw your consent at any time and for any reason, without having to tell us your reason. You will be fully cared for and supported as per your hospital's standard practice.

We will ask if you are happy for us to:

- Use any information already collected about you.
- Continue using information collected as part of your usual care until the end of the study.



For peer review only - http://bmjopen.bmj.com/site/about/guidelines.xhtml

You can change your mind about allowing your stored blood samples to be used for research at any time in the future, without giving any reasons, by contacting your local study team as listed on page x. Any samples left at the NICR will be destroyed. Any researchers to whom samples have been sent will be instructed to destroy the samples they have in their laboratories. It will not be possible to withdraw any findings from research work already undertaken using your donated samples.

## Can I take part in other research?

If you are already taking part in a clinical trial we would look to see what the trial involved and speak to the other trials team. We would need check that taking part in one trial would not affect the treatment or results in the other.

If you take part in VARIANT and want to join another trial in the future, we ask that you let the VARIANT team know so we can check with their team that there is no conflict. If there was a conflict, the study team would discuss your options with you and you can decide what it best for you.

For peer review only

## What if there is a problem?

If you are not happy with any part of VARIANT, you should ask to speak to the study team first who will do their best to help you. **Their contact details are on page X.** If you are still unhappy you may wish to raise your concerns with someone who is not directly involved in your care. You can contact the Patient Advice Liaison Service (PALS) who provide a confidential service on <site to localise with phone number and email address>

In the unlikely event that you are harmed during the research and this is due to someone's negligence (they were careless) you may have grounds for legal action for compensation, but you may need to meet your own legal costs. NHS indemnity does not offer no-fault compensation (for harm that is not anyone's fault).

## Will my GP be told about my involvement in VARIANT?

Yes, with your permission we will inform your GP that you are taking part in VARIANT. We will send you a copy of this letter so that you can see exactly what has been said. It will also be noted in your hospital medical records so that staff in the hospital know you took part in the study.

## What will happen to the results of the study?

- The study is due to finish at the end of 2020.
- The results will be written in medical journals and presented in meetings to other doctors, nurses and researchers.
- The anonymised data might be shared with other researchers and to help with future studies. Your identity will always be protected.
- A report will be written by the study funder and put on their website.

For peer review only - http://bmjopen.bmj.com/site/about/guidelines.xhtml

- You may request a summary of the results at the end of the trial by contacting the study team, their details are on page X.

## Will the information about me be kept confidential?

Yes. All of the information collected will be entered on computers that are kept secure and password protected.

- We will use a study number to identify you instead of using your name.
- Your contact details will never be shared with anyone else.
- You will not be named in any published results, reports or anything on our website.

The study information about you and your medical notes will be looked at by people directly involved in the study, clinical and lab staff as well as by people who are checking the study is running as it should. This may include the Newcastle Clinical Trials Unit at Newcastle University as they are managing the study. It may also include regulatory authorities, sponsor and funder.

Your local study team will collect information from you during the study. This will be entered onto a secure database using your study number instead of your name. Your local study team will be the only people that know that the study number refers to you. This means that the data on the database it is not identifiable to anyone outside of your local study team. The database is held by the Newcastle Clinical Trials Unit at Newcastle University. Access to this database is password protected and available to your doctors and research staff for the purpose of the trial. Data from the database will be analysed at the end of the whole study. Anonymised data from the database may be used when applying to carry out future related clinical trials or for other research projects related to prostate cancer.

After you have finished attending study visits, your local study team may review your health records and collect information for the study.

## Data Protection

All of the data we collect will be kept strictly confidential and in accordance with the General Data Protection Regulation (GDPR).

We have included information on page X of this document that tells you how we do this and included some websites where you can find out more.

## Who is organising and funding VARIANT?

The main study doctor (also called the 'Chief Investigator') is Mr Rakesh Heer, a Urological Surgeon at The Newcastle upon Tyne Hospitals NHS Foundation Trust. The study team includes senior doctors and nurses, university experts in research studies, and members of the public.

It is managed by the Newcastle University Clinical Trials Unit on behalf of the study sponsor – The Newcastle upon Tyne Hospitals NHS Foundation Trust. It is funded by the National Institute for Health Research, Research for Patient Benefit programme.


For peer review only - http://bmjopen.bmj.com/site/about/guidelines.xhtml

At least 3 hospitals will be taking part in VARIANT. Each hospital has a study doctor, called an 'Investigator'. The Investigator in your hospital is ……………………….

## Who has reviewed the study?

All research in the NHS is looked at by an independent group of people called a Research Ethics Committee. VARIANT has been reviewed and given a favourable opinion by the Wales REC 2 committee.

Patients have been involved in deciding how to do VARIANT from the start. For example, patients were involved in designing and applying for funding for the study, and continue to contribute as part of the study team. We also asked a group of patients and carers who have experienced cancer to look at the study information sheet to check the study is described in a clear way and is easy to understand.

For peer review only

## What if relevant new information becomes available?

The treatment management of metastatic prostate cancer is changing all of the time. All VARIANT patients will receive one of the standard treatment options available in the NHS. This will include any new treatment options that become available while the study takes place.

Information gathered during the course of this study will be reviewed by an independent Trial Oversight Committee. The role of this group is to protect the safety and wellbeing of participants by making sure the study is running safely.

## What if I have any questions?

Please ask the doctor or nurse who is looking after you. They can put you in touch with the research team or the Investigator for VARIANT at your hospital.

## What happens next?

You can take time to think about the study and whether you want to take part. A member of the research team will speak to you when you come back in to discuss your treatment options. They will go through this information sheet with you and answer any questions before you make your final decision.

## Thank you for taking the time to read this information sheet.

**VARIANT team contact details for your hospital:**

| | |
|---|---|
| **Principal Investigator:** | **Research Nurse:** |
| **Address:** | **Address:** |
| **Tel:** | **Tel:** |


For peer review only - http://bmjopen.bmj.com/site/about/guidelines.xhtml

## GDPR and Transparency Information

The Newcastle upon Tyne Hospitals NHS Foundation Trust (NUTH) is the sponsor for this study based in the United Kingdom and will act as the “data controller” for this study. **They are responsible for looking after your information and using it properly.**

This study is managed on behalf of the sponsor by the Newcastle Clinical Trials Unit who will act as the “data processor”. As data processor, this means that we are responsible for processing personal data on behalf of a controller. We will be using information from you in order to undertake this study, and will keep identifiable information about you for 10 years.

Your rights to access, change or move your information are limited, as we need to manage your information in specific ways in order for the research to be reliable and accurate. If you withdraw from the study, we will keep the information about you that we have already obtained. To safeguard your rights, we will use the least amount of personally-identifiable information possible.

You can find out more about how your information is used at http://www.newcastle-hospitals.org.uk/about-us/freedom-of-information_how-we-use-information

To find out more about research and general use of patient information please refer to the Health Research Authority Website https://www.hra.nhs.uk/information-about-patients/

The sponsor as an NHS Organisation and the Newcastle Clinical Trials Unit as a University use personally-identifiable information to conduct research to improve health, care and services. As a publicly-funded organisation, we have to ensure that it is in the public interest when we use personally-identifiable information from people who have agreed to take part in research. This means that when you agree to take part in a research study, we will use your data in the ways needed to conduct and analyse the research study.

Health and care research should serve the public interest, which means that we have to demonstrate that our research serves the interests of society as a whole. We do this by following the UK Policy Framework for Health and Social Care Research.

If you wish to raise a complaint on how we have handled your personal data, you can contact our Data Protection Officer who will investigate the matter. If you are not satisfied with our response or believe we are processing your personal data in a way that is not lawful you can complain to the Information Commissioner’s Office (ICO).

The sponsor Data Protection Officer is Richard Oliver and you can contact them at nuth.dpo@nhs.net.

The local study team at your hospital will collect information from you and/or your medical records for this research study in accordance with our instructions.

The local study team will use your name, NHS number and contacts details to contact you about the research study, and make sure that relevant information about the study is recorded for your care, and to oversee the quality of the study. Individuals from the sponsor, Newcastle Clinical Trials Unit and regulatory organisations may look at your medical and research records to check the accuracy of the research study. The local


For peer review only - http://bmjopen.bmj.com/site/about/guidelines.xhtml

study team will pass these details to the sponsor or the Newcastle Clinical Trials Unit along with information collected from you and/or your medical records. The only people at sponsor or the Newcastle Clinical Trials Unit who will have access to information that identifies you will be people who need to contact you or audit the data collection process. The people who analyse the information will not be able to identify you and will not be able to find out your name, NHS number or contact details.

The local study team will keep identifiable information about you from this study for 10 years after the study has finished.

When you agree to take part in a research study, the information about your health and care may be provided to researchers running other research studies in this organisation and in other organisations. These organisations may be universities, NHS organisations or companies involved in health and care research in this country or abroad. Your information will only be used by organisations and researchers to conduct research in accordance with the UK Policy Framework for Health and Social Care Research.

This information will not identify you and will not be combined with other information in a way that could identify you. The information will only be used for the purpose of health and care research, and cannot be used to contact you or to affect your care. It will not be used to make decisions about future services available to you, such as insurance.

For peer review only



For peer review only - http://bmjopen.bmj.com/site/about/guidelines.xhtml

## Further information about what will happen to blood samples

- Your samples will be sent securely to the Northern Institute of Cancer Research (NICR) for testing. For selected patients, a sample will also be sent to the All Wales Medical Genetics Laboratory (AWMGL) for testing (to confirm the results of the biomarker test).
- At the start of the study, your blood sample will be tested for the AR-V7 biomarker. Your doctor will receive the result of this test if you are in the group receiving treatment guided by AR-V7 blood test result. If you are in the treatment as usual group neither you nor your doctor will receive the result of the test.
- We may also test your blood sample for the AR-V7 biomarker at the end of the trial. These results will be used to inform the research. Neither you nor your doctor will not receive these results.
- Your blood samples will be stored at the NICR biobank (a collection of samples) and will be used in future research to look at why some patients develop advanced prostate cancer or how the cancer reacts to treatment. The DNA and RNA (the genetic material inside a cell), will be taken out of these stored samples and will also be stored at the NICR biobank. If you do not want your samples to be stored or used through the NICR biobank you will not be able to take part in this trial.
- Although unlikely for the VARIANT trial, there is a small possibility that your blood samples which have been stored in the NICR biobank may be used in research involving rodents (rats or mice). This is only done when it is essential to further our understanding of the way in which a disease develops or responds to treatment. These experiments are performed according to strict guidelines set out by the government and involve minimal stress to the rodents. **If you do not wish for your samples to be used in research using animals you should not complete the box referring to this on the consent form we will ask you to sign.** If you do not consent to your samples being used in research using animals you will still be able to participate in the VARIANT trial.
- If you are selected to provide an additional sample to be sent to AWMGL, the DNA and RNA (the genetic material inside a cell) may be taken out and stored at AWMGL before being transferred to the NICR biobank.
- You and your doctor will not find out the results of any tests done on your stored blood samples. Results of future research with your stored samples will be used to improve care of patients with advanced prostate cancer in the future.
- Your blood samples will be labelled with a unique study number. This number is used to link your sample back to you and only the study team and authorised people will be able to do this. Your sample will be sent with a form that includes your initials, date of birth and the date the sample was taken. This will help us make sure the samples can be linked back to the correct person which is especially important for feeding back the test results.
- Your stored samples may be used by researchers in the UK or overseas (including USA or Europe). Commercial partners may also use your samples for research

For peer review only



For peer review only - http://bmjopen.bmj.com/site/about/guidelines.xhtml

purposes. In most cases the commercial partners will be small companies that were started in universities (university/academic spin-off companies), or drug companies. Working with commercial partners is often important to have the resource to develop tests or products. We will ask partners to sign a legal contract to make sure samples are handled appropriately. Although the research will not be conducted to make money, it is possible that some of the results will be of value to commercial companies, for example in the development of new tests or treatments

- Any money made by sending samples to commercial laboratories will be put in to local research or used to improve patient care. Under UK law, sample donors are not entitled to a share of any profits that may result from this activity.
- The samples will be destroyed after 10 years of the last follow up for the last patient enrolled in the study. This will include all the blood samples and any linked data.

For peer review only


For peer review only - http://bmjopen.bmj.com/site/about/guidelines.xhtml

Consent Form

To be printed on local headed paper

# The Variant 7 Biomarker Feasibility Study

## The **VARIANT** Study

## PATIENT CONSENT FORM

**Site ID number:** ☐☐ **Participant ID Number:** ☐☐☐

**Principal Investigator Name:** ____________

**Please INITIAL these boxed if you agree:**

| | |
|---|---|
| 1. I have read and understood the Patient Information Sheet version _______ dated_____ for the above study. I have had the opportunity to consider the information, ask questions and I am happy with the answers given. | |
| 2. I understand that I do not have to take part in this study. I know that I can withdraw at any time without giving a reason and without my medical care or legal rights being affected. | |
| 3. I understand that information about me will be collected, recorded and used for this study unless I withdraw my consent. I understand that my information will be kept securely and confidentially. | |
| 4. I agree for a copy of my consent form to be sent securely to the Newcastle University Clinical Trials Unit for checking. | |
| 5. I agree to my General Practitioner (GP) being informed about me taking part in this study. | |
| 6. I understand that relevant sections of my medical notes and information collected during the study may be looked at by people from Newcastle Clinical Trials Unit, the study sponsor, regulatory authorities and the local NHS Trust, where it is relevant to my taking part in this research. I give permission for these individuals to have access to my records. | |

For peer review only



For peer review only - http://bmjopen.bmj.com/site/about/guidelines.xhtml

Consent Form

| | |
|---|---|
| 7. I understand that any personal information collected for the study will be kept confidential and not be made public. I understand that anonymised data from the study may be used for other research projects related to prostate cancer and will be published in medical journals, at research meetings and shared with other researchers. | |
| 8. I consent to relevant sections of my medical notes being accessed by members of my local study team to carry out follow-up, including after my participation in the trial has ended, and for there to be a link between my healthcare records and the data collected for the trial. | |

**Sample collection**

| | |
|---|---|
| 9. I consent to the collection of my blood samples as described in the VARIANT patient information sheet and understand that my samples will be sent to the Northern Institute of Cancer research (NICR) and the All Wales Medical Genetic Lab (AWMGL) for testing. | |
| 10. I understand that my samples will not be identifiable except to the trial management team and I give permission for my date of birth and initials to be sent with the blood samples to the lab and with the biomarker result to my study doctor. | |
| 11. I consent to my samples being stored in the NICR biobank for up to 10 years, and give permission for samples to be used as described in the VARIANT Patient Information Sheet. I understand that the biobank will keep my identity confidential and any information collected about me during the study will be anonymised in a way that protects my identity. | |
| 12. OPTIONAL: I consent to my samples to be used in experiments using rodents (rats or mice). | |

For peer review only

**Agree to Participate**

| | |
|---|---|
| 13. I agree to take part in the VARIANT study. | |

______________________ ______________________ ______________________

**Name of patient** **Signature** **Date**

______________________ ______________________ ______________________

**Name of Person** **Signature** **Date**



For peer review only - http://bmjopen.bmj.com/site/about/guidelines.xhtml

Consent Form

**taking consent**

**When completed: 1 copy for participant; 1 copy (original) for Investigator Site File; 1 copy to be kept in medical notes and 1 copy to be sent securely to Newcastle Clinical Trials Unit**

For peer review only


For peer review only - http://bmjopen.bmj.com/site/about/guidelines.xhtml

Completed SPIRIT checklist for VARIANT trial protocol submission:

Administrative Information:

1. Title
2. Trial Registration
3. Protocol Version
4. Funding
5. Roles and Responsibilities

Introduction:

6. Background and Rationale
7. Objectives
8. Trial Design

Methods: Participants, interventions, Outcomes

9. Study Setting
10. Eligibility criteria
11. Interventions
12. Outcomes
13. Participant timeline
14. Sample size
15. Recruitment

Methods: assignment of interventions (for controlled trials)

16. Allocation
17. Blinding (masking)

Methods: data collection, management, analysis

18. Data collection methods
19. Data management
20. Statistical methods

Methods: monitoring

21. Data monitoring
22. Harms
23. Auditing

Ethics and dissemination:

24. Research ethics approval
25. Protocol amendments
26. Consent or assent
27. Confidentiality
28. Declaration of interests
29. Access to data
30. Ancillary and post-trial care
31. Dissemination policy

Appendices

32. Informed consent materials
33. Biological specimens

For peer review only

For peer review only



For peer review only - http://bmjopen.bmj.com/site/about/guidelines.xhtml

BMJ Open

# The Prostate Cancer Androgen Receptor Splice Variant 7 Biomarker Study - A multicentre randomised feasibility trial of biomarker-guided personalised treatment in patients with advanced prostate cancer (The VARIANT Trial) study protocol.


| | |
|---|---|
| Journal: | *BMJ Open* |
| Manuscript ID | bmjopen-2019-034708.R1 |
| Article Type: | Protocol |
| Date Submitted by the Author: | 21-Nov-2019 |
| Complete List of Authors: | Clark, Emma; Newcastle University, Translational and Clinical Research Institute<br>Morton, Miranda; Newcastle University, Newcastle Clinical Trials Unit<br>Sharma, Shriya; Newcastle University, Newcastle Clinical Trials Unit<br>Fisher, Holly; Newcastle University, Population Health Sciences<br>Howel, Denise; Newcastle University, Population Health Sciences<br>Walker, Jenn; Newcastle University, Newcastle Clinical Trials Unit<br>Wood, Ruth; Newcastle Clinical Trials Unit,<br>Hancock, Helen; Newcastle University, Newcastle Clinical Trials Unit<br>Maier, Rebecca; Newcastle University, Newcastle Clinical Trials Unit<br>Marshall, John<br>Bahl, Amit; University Hospitals Bristol NHS Foundation Trust<br>Crabb, S; University of Southampton<br>Jain, Suneil; Queen's University Belfast<br>Pedley, Ian; Newcastle Upon Tyne Hospitals NHS Foundation Trust<br>Jones, Rob; University of Glasgow<br>Staffurth, John; Velindre Cancer Centre, Research; Cardiff University School of Medicine, Division of Cancer and Genetics<br>Heer, Rakesh; Newcastle University, Translational and Clinical Research Institute |
| <b>Primary Subject Heading</b>: | Oncology |
| Secondary Subject Heading: | Urology |
| Keywords: | Protocols & guidelines < HEALTH SERVICES ADMINISTRATION & MANAGEMENT, Prostate disease < UROLOGY, Adult oncology < ONCOLOGY, Urological tumours < ONCOLOGY, Cell biology < BASIC SCIENCES |
| | |

SCHOLARONE™
Manuscripts

## Title

The Prostate Cancer Androgen Receptor Splice Variant 7 Biomarker Study - A multicentre randomised feasibility trial of biomarker-guided personalised treatment in patients with advanced prostate cancer (The VARIANT Trial) study protocol.

Authors: Emma Clark[1], Miranda Morton[2], Shriya Sharma[2], Holly Fisher[3], Denise Howel[3,] Jenn Walker[2], Ruth Wood[2], Helen Hancock[2], Rebecca Maier[2], John Marshall[4], Amit Bahl[5], Simon Crabb[6], Suneil Jain[7], Ian Pedley[8], Rob Jones[9*], John Staffurth[10]* and Rakesh Heer[1]*
*Joint senior authors

1. Translational and Clinical Research Institute, Faculty of Medical Sciences, Newcastle University, Newcastle upon Tyne, UK
2. Newcastle Clinical Trials Unit, Newcastle University, Newcastle Upon Tyne, UK
3. Population Health Sciences Institute, Faculty of Medical Sciences, Newcastle University, Newcastle Upon Tyne, UK
4. VARIANT Trial patient representative
5. Department of Clinical Oncology, Bristol Cancer Institute, Bristol, UK
6. Southampton Experimental Cancer Medicine Centre, University of Southampton, Southampton, UK
7. Radiotherapy Department, Cancer Centre, Belfast City Hospital, Belfast, Northern Ireland, UK
8. Northern Centre for Cancer Care, Freeman Hospital, Newcastle-upon-Tyne, UK
9. Beatson West of Scotland Cancer Centre, University of Glasgow, Glasgow, UK
10. Velindre Cancer Centre, Cardiff University, Cardiff, UK

Corresponding authors:

1. Emma Clark:

• e-mail: emma.clark@ncl.ac.uk

• Phone: 0191 208 4456

2. Rakesh Heer:

• e-mail: rakesh.heer@ncl.ac.uk

• Phone: 0191 208 4300

Key Words:

- Prostate Disease
- Protocols and guidelines
- Adult Oncology
- Urological tumours
- Cell biology

This study opened to recruitment on the 09/07/2019 with the first patient consented 29/07/2019 and is expected to report in 18 months. This protocol is the current approved VARIANT protocol Version 2.0 8th March 2019



For peer review only

For peer review only - http://bmjopen.bmj.com/site/about/guidelines.xhtml

## Abstract

**Introduction:**

Prostate Cancer is the most common male cancer with 1 in 4 developing non-curable metastatic disease. Initial treatment responses to hormonal therapies are transient and further management options lie between [1] further hormone therapy or [2] a non-hormonal approach involving additional chemotherapy or molecular radiotherapy (radium-223). There is no clear rationale for choosing between these mechanistically different treatment approaches. The biology of hormone resistance is driven through abnormal androgen receptor activity and we can assay this through a blood test measuring androgen receptor variant 7 (AR-V7) expression in circulating tumour cells (CTCs). Despite increasing evidence supporting AR-V7's role as a prognostic marker, the clinical utility of such measures remains unknown in helping personalise treatment decisions.

**Methods and Design:**

The VARIANT feasibility trial is a pragmatic design, to be run over 18 months with participants randomised into the intervention arm receiving biomarker (AR-V7) guided clinical treatment and participants randomised into the control arm with conventional standard management (no biomarker guidance). AR-V7 positive participants (likely to be insensitive to further hormone treatment) will receive chemotherapy or in other cases radium-223 (where routinely available). Seventy male ≥18 years old participants with metastatic castrate resistant prostate cancer clinically indicated to proceed to further hormone therapy or chemotherapy, will be recruited from three National Health Service (NHS) Trusts based in England, Scotland and Wales. The feasibility primary outcome is willingness of patients to be randomised and clinicians to recruit to a biomarker-based treatment strategy, with trial data informing the basis of a definitive and appropriately powered randomised control trial (RCT).

**Ethics and Dissemination:**

Formal ethics review was undertaken with a favourable opinion, through Wales NRES Committee 2 18/WA/0419. Findings to be disseminated through patient and professional organisations that have expressed their support, media outlets and peer-reviewed journal publication.


## Article Summary

**Strengths and limitations of this study:**

- **Focuses on a priority area of need in advanced prostate cancer clinical practice.**
- **To date, the feasibility of delivering a randomised biomarker guided-treatment trial in prostate cancer to formally assess clinical utility is not established and will be addressed in this study.**
- **As a feasibility study, the planned sample size (70 participants) does not have sufficient power or precision to compare the 'event' rate between treatment arms, but will allow informed planning for a definitive randomised controlled trial with prominent clinicians from non-recruiting centres involved in feasibility, to aid with follow-on trial.**



For peer review only - http://bmjopen.bmj.com/site/about/guidelines.xhtml

- **Emerging evidence points to additional Androgen Receptor (AR) biology driving hormone resistance, such as other variant expression and mutations – these along with alternative biomarkers can be explored in the associated biobanked samples (including cell free tumour DNA).**
- **Strong patient and public involvement to inform study design with a clear commitment to informing participants of project outcomes setting a clear new gold standard for PPI.**

## INTRODUCTION

### Background

Prostate cancer (PC) is the most common male cancer in the UK and the second highest cause of male cancer death(1). In large part, PC is a slowly progressive disease and when detected at an early stage is managed by active surveillance, surgery or radiotherapy. However, 25% of patients will present with, or will progress to, advanced metastatic PC(2,3). Metastatic PC is incurable, with less than one third of patients surviving more than 5 years(1).

Medical castration (commonly referred to as hormonal treatment or androgen deprivation therapy (ADT)), blocks production of the hormone testosterone and/or targets the androgen receptor (AR) signalling axis that drives cancer cell growth. Although a good response to hormonal treatment seen often initially, disease progression to a lethal metastatic Castration-Resistant Prostate Cancer (mCRPC) is common(4). Clinical trials have shown that the addition of chemotherapy (docetaxel) or other hormonal approaches (abiraterone acetate or enzalutamide) to initial hormonal therapy have led to a substantial improvement (i.e. delay) in time to the development of mCRPC and overall survival (OS)(5-9). Furthermore, promising recent evidence from randomised trials of androgen-receptor axis-targeted drugs (ARATs) have shown addition of Apalutamide (an inhibitor of the ligand-binding domain of the AR), alongside hormone therapy results in longer overall survival and radiographic progression free survival compared to placebo(10). However despite these rapid advances, mCRPC typically manifests within 3 years and is uniformly fatal(11-13).

### Treatment management for mCRPC

Management pathways for mCRPC are still evolving in response to emerging new treatments however, it broadly follows one of two standard care approaches(14); [1] further hormonal treatment such as abiraterone or enzalutamide or [2] 'non-hormonal' treatment, typically chemotherapy or molecular radiotherapy (radium 223) (where available). There is no clear biological rationale for choosing between these mechanistically different treatment approaches. Suitable patients for this study can receive both approaches in a sequential manner if one is failing. Patients and clinicians often prefer hormonal treatment, being less toxic and easier to manage, however, only 30-50% of men respond well, with the remainder demonstrating a poor or an equivocal response(15,16). As many patients will not respond to either treatment approach, there are considerable costs from our current management pathways, both in terms of patient experience and outcomes (side effects and disease progression) and economic costs to the NHS (large burden of expensive treatments for the commonest male cancer). Personalised management pathways are urgently needed.

### Biology of the Androgen Receptor (AR selective treatment pressure)



For peer review only - http://bmjopen.bmj.com/site/about/guidelines.xhtml

A breakthrough in understanding the biology of PC revealed that hormonal treatments generate a selective pressure at the cellular level inducing complex molecular mechanisms characterised by an adaptation of the androgen-AR signalling axis. This results in tumour resistance mediated by the induced expression of alternative types of androgen receptor. These AR mRNA splice variants lack the important hormone-binding domain, resulting in a constitutively active cellular receptor, despite castration. The most widely studied variant is AR-V7(17,18). AR-V7 activity is not affected by 'hormonal treatment' such as enzalutamide and abiraterone that target the hormone-binding domain, potentially rendering these treatments ineffective in men with AR-V7(19-21). A surge in ARATs available for clinical use (e.g. Apalutamide and Darolutamide) will most likely enhance this burden (although of note, evidence demonstrating reduced effectiveness of these specific treatment in men who are positive for AR-V7 or other variant splice forms including AR point mutations, have not been published to date).

**Rationale**

Published clinical data demonstrates a strong link between AR-V7 expression and mCRPC progression and highlights the potential for AR-V7 to be utilised as a treatment stratification biomarker to identify those men likely to be sensitive to further hormonal treatment (AR-V7-ve patients) and avoid futile treatments in those predicted to be insensitive (AR-V7+ve patients)(21-29). Notably AR-V7 positivity is not associated with insensitivity to taxane chemotherapy treatment (relative reduction in risk of death of 76% maintained with chemotherapy, hazard ratio: 0.24; 95% CI, 0.10-0.57; P = 0.035)(27,30) and data from the recent PROPHECY trial reports on the prognostic value of the AR-V7 biomarker (prospective observational cohort of poor prognosis patients with advanced prostate cancer who receive abiraterone or enzalutamide treatment)(30). The commercially available AdnaTest ProstateCancerPanel AR V7 assay (Qiagen®) detects AR-V7 mRNA expression in circulating tumour cells (CTCs) in whole blood and has been independently and robustly clinically validated in terms of reproducibility and comparisons of sensitivity and specificity with other AR-V7 detection platforms(31-33). However to date, there have been no formal measures of the clinical utility of AR-V7 as a predictive biomarker

**Evidence gap**

Encouragingly, a cost saving analysis of performing ProstateAdnaTest AR-V7 biomarker testing in mCRPC demonstrated use of the biomarker would result in a substantial cost saving as long as the true prevalence of AR-V7 was >5% (well below the accepted prevalence rate of 30%)(34). However, formal cost effectiveness analyses based on incremental cost-effectiveness ratio (ICER) (cost per quality-adjusted life year gained) and assessing prevalence rates of this biomarker have yet to be carried out.

The National Comprehensive Cancer Network Task Force(35) and CRUK consensus statement on biomarker roadmap for cancer studies(36), have highlighted the key recommendations for accelerating a tumour biomarker into clinical practice by sequentially demonstrating evidence for; (1) analytic reproducibility; (2) clinical validity and; (3) clinical utility. Previous clinical studies on AR-V7 testing focused on retrospective or prospective cohort analyses of associated AR-V7 expression distinguishing subgroups with different clinical outcomes with hormonal treatment in men with metastatic PC(37-40). However, the highest level of assessment of clinical worth in improving patient outcomes (clinical utility) remains lacking. We have paid



For peer review only - http://bmjopen.bmj.com/site/about/guidelines.xhtml

particular focus to address clinical utility evidence gaps in the VARIANT trial using published levels of evidence standards for assessing biomarkers to inform study design(41-42). We aim to demonstrate improvement in patient outcome sufficiently to justify AR-V7 biomarker incorporation into routine clinical care (including feasibility of collecting quality of life measures for a future health economic evaluation).

**Emerging treatment landscape**

The treatment landscape of hormone sensitive (PC) is evolving, altering treatment pathways for mCRPC. Recent data from the USA based PROPHECY trial reporting on a prospective observational cohort showed mRNA AR-V7 (modified Qiagen ProstateCancerAdnaTest, Baltimore, MD) and protein AR-V7 (Epic nuclear-specific, San Diego) biomarker positivity associated with worse progression free survival (PFS) and overall survival (OS) in poor prognosis patients with advanced prostate cancer who receive abiraterone or enzalutamide treatment(30). Criticisms of the study included lack of testing with alternative treatment such as chemotherapy (which we have addressed in this study) and pre-selection of high risk CRPC patients (i.e. those with poor prognosis), ultimately generating results that cannot be extrapolated over the overall CRPC population(43-46). Of note, lower AR-V7 prevalence was reported in the overall CRPC population in the ARMOR3-SV phase III clinical trial which employed the Adnatest ProstateCancerSelect and Detect CTC assay (Qiagen®) to assess AR-V7 mRNA expression, where only 8% of men were AR-V7 positive (95% CI 6-10)(47,48). During reviewing of this protocol, results of the CARD trial (Cabazitaxel versus Abiraterone or Enzalutamide in Metastatic Prostate Cancer) were published showing median overall survival was 13.6 months with cabazitaxel and 11.0 months with androgen signalling targeted inhibitors (hazard ratio for death, 0.64; 95% CI, 0.46 to 0.89; P=0.008). CARD investigators plan to analyse CTCs for AR-V7 in order to determine the prognostic and predictive value of CTC-derived AR-V7 detection, further contributing important findings from this evolving treatment landscape(49).

We argue irrespective of the evolving treatment landscape, the opportunity to generate feasibility data for a biological (biomarker) informed approach to treatment selection over standard care protocol-based approaches, tests a highly relevant clinical question in these high risk CRPC patients (i.e. those who have more to loose from pursuing a ‘try and see’ approach). This would provide an appealing long-term strategy (for patients and service providers) to ultimately improve on clinical outcome (specifically for a clinical subgroup of poor prognosis patients, identifying those likely to be sensitive to further hormonal treatment and avoid futile treatments in those that are predicted to be insensitive).

**<u>Main Aim of Study</u>**

To determine the feasibility of conducting a definitive randomised control trial to evaluate the clinical utility of an AR-V7 blood biomarker assay in personalising treatment for men with mCRPC in UK NHS clinical practice.

**<u>Objectives</u>**

Feasibility study



For peer review only - http://bmjopen.bmj.com/site/about/guidelines.xhtml

**Primary:**

1. To establish if it is feasible to conduct a definitive trial comparing AR-V7 biomarker-driven management with the current standard care in patients with mCRPC.

**Secondary Objectives:**

2. To estimate AR-V7 biomarker prevalence in the trial population to inform sample size calculations for a definitive randomised control trial.

3. To assess recruitment, compliance and retention rates
4. To confirm outcome measures for a future definitive trial and establish trial data response rates, variability, and data quality.
5. To establish a blood sample biorepository to include baseline, 12 and 24 week blood samples for future translational studies.

**Exploratory objectives:**

6. To establish a complete serial blood tissue archive to include potential measures of cell free DNA and additional AR-Variants in CTCs and cfDNA biomarker measures (such as AR mutations, other AR splice forms and AR amplification and other mutations such as PTEN/p53/MYC gain/RB1 loss/MET gain and further molecular pathways yet to be defined), to complement AR-V7 reads, depending upon the ultimate biomarker performance characteristic established in this trial population. Blood will be collected, processed and archived at 0 weeks (baseline), 12 weeks and 24 weeks following the first treatment.
7. To explore thresholds of the magnitude of AR-V7 positivity to investigate relationships with outcomes and to estimate AR-V7 positivity rate assumptions regarding a cut-off point.
8. To undertake cross site validation of biomarker reads between two GCP laboratories (Newcastle University and Cardiff University).

## METHODS AND ANALYSIS

**Study design**

This feasibility study is a multi-centre, two-arm, randomised control trial (RCT). All patients who consent to take part in the trial and who are eligible, will have a blood test to assess prevalence rate of the AR-V7 biomarker. Participants will be randomised in the ratio 1:1 to receive personalised standard treatment (intervention) guided by AR-V7 biomarker status or standard care (control) without biomarker guided treatment. Those in the control group will not receive blood biomarker test results.

The treatment for each patient is expected to be dependent on various factors (e.g. clinician choice, patient choice, previous treatments, co-morbidities, concomitant medication and pattern of disease) as well as randomised allocation and AR-V7 status in the personalised treatment arm. All treatments are part of standard care for these participants. Treatment options for participants randomised to the personalised standard treatment arm will be recommended, but not mandated within this feasibility trial, with reasons for not following the



For peer review only - http://bmjopen.bmj.com/site/about/guidelines.xhtml

recommendation recorded and reported as outcome. A CONSORT diagram of study protocol (version 2.0 8th March 2019) is shown in figure 1.

**Study Setting**

Seventy patients with mCRPC who require a change in treatment will be recruited in three secondary care NHS Trusts in the UK spread across England (The Newcastle upon Tyne Hospitals NHS Foundation Trust), Scotland (NHS Greater Glasgow and Clyde) and Wales (Velindre University NHS Trust). We aim to recruit mCRPC patients with a predicted poor overall survival. We anticipate this group of mCRPC patients have the most to gain from a biological-based treatment approach as their disease is more likely to progress during a period of treatment with an inactive agent. Multivariate analysis from the metastatic population of STAMPEDE(50) has shown that worse overall survival was seen in men with the following features: presence of bone metastases (regardless of soft tissue metastases), worse WHO performance status (0 vs 1 or 2), higher (or unknown) initial Gleason sum score category (≥8 vs ≤7), and younger age at randomisation <60yrs. Poorer failure free survival (but not overall survival was additionally seen in men with worse primary tumour stage and higher PSA level before starting ADT. There is overlap between these poor prognostic features and factors associated with a high likelihood of harbouring AR-V7+ve CTCs(33, 40, 51).

**Eligibility Criteria**

Patients will be aged ≥18 years old with metastatic castrate resistant prostate cancer (high risk features) clinically indicated to proceed to further hormone therapy or chemotherapy and fulfil all of the following criteria:

1. Histologically or cytologically proven diagnosis of adenocarcinoma of the prostate.
2. Radiographic and/or histological and/or cytological evidence of metastatic disease.
3. Castrate levels of testosterone and documented ongoing medical or surgical castration. Testosterone level ≤50ng/dl /1.73 nmol/L and maintaining on androgen suppression therapy.
4. Disease progression since the last change in therapy defined by one or more of the following: (i) PSA progression as defined by the prostate cancer working group 3 (PCWG3) criteria ≥ 2ng/ml; (ii) bone disease progression as determined by the local radiology/ multidisciplinary team; (iii) radiographic progression of nodal or visceral metastases as determined by the local radiology/ multidisciplinary team.
5. Suitable for treatment with at least one novel hormonal treatment (with available treatments abiraterone acetate or enzalutamide) and one non-hormonal therapy (with available treatments docetaxel, cabazitaxel or radium-223).
6. At least two high risk features: (i) age <60 years at time of diagnosis of metastatic disease; (ii) bone metastases present at time of initial metastatic prostate cancer diagnosis (although not mandated, it is considered good clinical practice to have up to date imaging within 8 weeks); (iii) Gleason grade group 4 or 5 (Gleason score 8 to 10); (iv) presence of visceral metastases (e.g. liver or lung) at any time point. This does not include lymph node metastases; (v) PSA doubling time < 3 months; (vi) elevated alkaline phosphatase above institutional upper limit of normal; (vii) ECOG



For peer review only - http://bmjopen.bmj.com/site/about/guidelines.xhtml

Performance Status worse than or equal to 1; (viii) previous treatment for castration resistant prostate cancer with docetaxel chemotherapy; (ix) previous treatment for castration resistant prostate cancer with abiraterone and/or enzalutamide or equivalent agent.

7. Estimated life expectancy > 6 months.
8. Provision of written informed consent, including consent for bio-banking of blood samples.

Exclusion Criteria applied in the VARIANT trial are:

1. Histological variants of prostate cancers with small cell or neuroendocrine features.
2. Prior or current malignancy (except adenocarcinoma of the prostate) with an estimated ≥ 30% chance of relapse/progression within next 2 years.
3. Previously identified brain metastases or spinal cord compression unless treated with full functional recovery.
4. Administration of an investigational agent within 30 days of first dose of trial medication.

**Randomisation**

Patients will be randomised to receive either personalised standard treatment (guided by AR-V7 biomarker status) or standard care (not guided by biomarker status) on a 1:1 basis using a method of random permuted blocks of concealed variable block size and stratified by site.

**Study Intervention**

This three-centre randomised feasibility study incorporates a control and an intervention arm. All patients will undergo AR-V7 biomarker assessment with results only made known to the patients and clinical team in the intervention arm.

**Intervention arm**

Treatment will be given as per standard care with recommendations guided by biomarker status; (1) If the participant is found to be AR-V7 positive, then non-hormonal treatment is recommended (docetaxel chemotherapy, cabazitaxel chemotherapy or radium-223 therapy) or (2) if the participant is found to be AR-V7 negative, then next generation hormonal treatment is recommended (either enzalutamide or abiraterone).

The results of the AR-V7 biomarker assessment will be provided securely to the clinical team to enable tailored treatments based on AR-V7 expression from the biomarker result. Where a decision is made that the participant will receive a non recommended therapy (either by the clinician or patient) this therapy, and the reasons for giving this, will be documented.

**Control arm**

Participants with their clinical care team will make an informed and preference-based decision to receive standard care, including either next generation hormone treatments abiraterone or enzalutamide or non-hormonal approaches including docetaxel or cabazitaxel chemotherapy or radium-223. Details of all treatment administered, including doses, will be recorded as part of the trial.

The research team at sites will not receive the participants AR-V7 biomarker results.

**<u>Outcome Measures</u>**

Standardised clinical assessment tools used in monitoring CRPC disease and progression on treatment will be reported (listed in box 1). Primary outcome measures are related to feasibility (recruitment, retention and adherence) and will report the following;



For peer review only - http://bmjopen.bmj.com/site/about/guidelines.xhtml

(1) the proportion of prostate cancer patients identified through clinics who meet the eligibility criteria;
(2) the number of patients accrued per site per month over the course of the trial;
(3) baseline prevalence of AR-V7 expression in the participant cohort (this will be presented as a crude percentage of AR-V7 positivity of total participants, and in each arm);
(4) the willingness of patients to be randomised (defined as the proportion of patients consenting to be randomised from all eligible patients approached about the study);
(5) compliance rate (this will be defined as the number of patients who start randomised treatment as a proportion of the number randomised);
(6) the proportion of patients who: start AR-V7 recommended treatment; start treatment other than the recommended treatment; change treatment before disease progression; or withdraw. (This measure will capture information regarding patients who choose not to take recommended treatment because of strong preferences and patients who progress rapidly while waiting for treatment with a change in eligibility for treatment options).
(7) the proportion of trial participants with assessable blood samples for biomarker status (which would affect treatment targeting);
(8) the median time from the blood sample being drawn to; (i) AR-V7 result being sent back to the site and (ii) patient starting treatment (and compared with standard of care treatment).
(9) the proportion of randomised patients for whom data is collected on each clinical and health economic outcome at baseline, 12 and 24 weeks.

| Box 1 Standardised Clinical Assessment Tools |
|---|
| Clinical Outcome Measures: |
| (1)Time to PSA progression; (i) Confirmed rising PSA more than 12 weeks after randomisation. (*Where there has been a decline in PSA from baseline, progression will be a 25% or greater increase, and an absolute increase of at least 2ng/mL, from the nadir, which is confirmed by a second value obtained 3 or more weeks later. Where no decline from baseline is documented, progression must be a 25% or greater increase from the baseline value along with an increase in absolute value of 2 ng/mL or more. In all cases, the initial rise in PSA must occur after a minimum of 12 weeks from randomisation*). |
| (2)Clinical progression and survival within 6-months; (i) Number of patients who have progressed clinically at 6-months (includes change of systemic anti-cancer therapy and death from PC); (ii) Cancer specific survival at 6-months; and (iii) overall survival at 6-months. |
| (3)Quality of life for patients with cancer (EORTC QLQ-C30). |
| (4)Additional quality of life items patients with prostate cancer (EORTC QLQ-PR25). |
| (5)Participant costs questionnaire (Use of Health Services Questionnaire). |

Further information on recruitment, screening, the patient consent procedure and informed consent literature can be found in the supplementary section.

## Data collection

Table 1 shows a trial schedule of events. A more detailed description of all data collection including a data management plan, can be found in the supplementary section. In summary, in addition to collecting standard care assessment of disease status data from patients in the



For peer review only - http://bmjopen.bmj.com/site/about/guidelines.xhtml

356 intervention and control arms, trial specific questionnaire assessment (EORTC QLQ-C30
357 Quality of Life of Cancer Patients, EORTC QLQ-PR25 Quality of Life Prostate Cancer
358 Module) will take place at the baseline, 12 and 24 week visit.

| **Procedure** | **Screening** | **VISIT 1 Consent/ Baseline** | **VISIT 2 12 weeks (+/- 2 weeks)** | **VISIT 3 24 weeks (+/- 2 weeks)** |
|---|---|---|---|---|
| Medical History and Demographics | X | | | |
| Record results of standard care PSA test | X | X | X | X |
| Eligibility Assessment | X[a] | X[a] | | |
| Patient Information Sheet | X | | | |
| Informed consent | | X | | |
| Testosterone if no previous confirmation | | X[b] | | |
| Confirmation of eligibility | | X[a] | | |
| Randomisation | | X | | |
| Access to standard of care haemoglobin and biochemistry results | | X | | |
| Blood sample collection and shipment for CTC/ctDNA blood assessment and AR-V7 analysis (analysed at Newcastle University Central Analysis Lab) | | X | X | X |
| CTC blood sample collection and shipment for cross site validation[c] (analysed at Cardiff University Central Analysis Lab) | | X[c] | | |
| EORTC QLQ-C30/PR25 Questionnaires | | X | | X |
| AR-V7 blood test result feedback to patient[d] | | X[d] | | |
| Use of Health Services Questionnaire | | | | X |



For peer review only

| | | | | |
|---|---|---|---|---|
| Anti-cancer therapy review | | | X | X |
| Clinical assessment of disease status | | | X | X |

Table 1. Trial Schedule of Events

a = Eligibility assessment performed against trial eligibility criteria in screening, patients likely to be eligible will be given a VARIANT Information Sheet and trial information. Eligibility will be confirmed by an Investigator (medically qualified doctor) after patients have provided written informed consent and before randomisation.

b = in those cases where there is no previous confirmation of castrate levels of testosterone only. These patients will not be randomised until castration is confirmed and the patient is documented as eligible.

c = for selected patients only (confirmed at randomisation), for cross site validation of AR-V7 status

d = for patients randomised to the personalised standard treatment arm (guided by AR-V7 biomarker) only.

**AR-V7 biomarker measure**

A validated two-centre pipeline (consisting of preanalytical, analytical and postanalytical phases) to measure AR-V7 biomarker using the commercially available AdnaTest ProstateCancerPanel AR-V7 circulating tumour cell (CTC) quantitative RT-PCR (RT-qPCR) assay (Qiagen®) (intended for molecular biology applications), has been set up according to assay manufacturers recommendations, analytical methods and sponsor agreed SOP's. Following biomarker data analysis and data verification, for participants randomised to the intervention standard treatment arm (AR-V7 biomarker guided), the baseline biomarker result and biomarker treatment recommendation will be sent securely within 10 working days to the local PI and delegated research staff. Further information on the specifics of AR-V7 biomarker driven personalised treatment (sample receipt, processing, analysis and reporting of read-out) can be found in the supplementary section.

**Data Analysis Plan**

Analyses will be conducted on an intention-to-treat basis, with sensitivity analyses used to investigate the impact of non-compliance to allocated arm. Given the feasibility status of this study, all statistical analyses will be descriptive. The majority of the outcome data will be presented in simple descriptive tables presenting percentages, means and standard deviations or 5-number summary (as appropriate), for each arm of the study. Analysis of clinical and biomarker measures will be assessed by; (1) clinical progression and survival within 6-months; (2) PSA response/progression (confirmed rising PSA more than 12 weeks after randomisation); (3) clinical progression and survival (overall and cancer specific) within 6-months (includes change of cancer therapy for progression); and (4) survival (overall and progression free)



For peer review only - http://bmjopen.bmj.com/site/about/guidelines.xhtml

estimates will be derived using the Kaplan Meier method and presented as 6-month rates with confidence intervals. The relationship between survival estimates and continuous AR-V7 biomarker expression will be modelled considering non-linear transformations in a univariate Cox model, or parametric alternative, presented as parameter estimates (HR) with confidence intervals.

Compliance with quality of life and health economic measures will be assessed by; (1) number of patients completing measures as a proportion of the number randomised; (2) degree of completeness of each domain of the EORTC QoL and economic questionnaire measures. These scores will be presented graphically and with numeric descriptive statistics..

**Study statistical size calculations**

This trial is designed as feasibility trial according to definition of Eldridge *et al.*(52). Feasibility includes the deliverability of the intervention and in this case, assessment of the frequency of the positive assay measurements (predicted at approximately 30%). It has been recommended that data in an external pilot trial is collected on a minimum of 60 patients per arm to estimate the 'event' rate(53). However, we plan to calculate a pooled estimate of overall recruitment rate, and overall biomarker prevalence rate, and will recruit 70 patients in total to allow for attrition.

The performance of potential outcome measures for a definitive trial will be assessed by estimating data completeness of the instruments and any potential bias in the completion of follow-up data. This information will be used to inform the design, choice of outcomes, necessary sample size and approach to the analysis, of a future definitive trial.

**Safety Reporting**

This is a low risk trial and no specific safety reporting is required. Should an Investigator have any concern regarding participant safety as an outcome of their participation in the trial, they will contact the Trial Management Group (TMG) and Chief Investigator as soon as possible. The Trial Oversight Committee (TOC) will monitor concerns as required.

**Trial Conduct and Governance**

The trial will be conducted in accordance with the UK Policy Framework for Health and Social Care and, as applicable, the Guidelines for Good Clinical Practice. The TMG is responsible for the day-to-day management of the trial, overseeing all aspects of the conduct of trial to ensure that the protocol is adhered to and take appropriate actions to ensure patient and data safety. The TOC will review trial conduct and accumulating clinical trial data and provide overall supervision for the trial on behalf of the Sponsor and the Funder. The constitution of the TMG and TOC including roles and responsibilities delegation for this trial can be found in the supplementary section. Aggregated data will be analysed by the Trial Statisticians and reported to an external independent TOC at least annually.



For peer review only - http://bmjopen.bmj.com/site/about/guidelines.xhtml

## Public and Patient Involvement

The design, planning and management of this trial has been supported by two prostate cancer patient representatives (co-applicant on the funding grant and TMG member). Both have advocated the dissemination of trial findings to patients and ensured that the public was adequately considered during trial design. PPI has been embedded into the study, with the patient's voice a strong theme to inform and influence the on-going research and development of participant information resources in collaboration with the 'Cancer Perspectives' patient representative group (Newcastle upon Tyne Hospitals NHS Foundation Trust). A strong commitment is to inform the participants of the outcome of this project, a clear new gold standard for PPI.

## Ethics and dissemination

Favourable ethical opinion has been obtained from the Wales National Research Ethics Service (NRES) Committee 2 18/WA/0419. All parties will conduct the trial in accordance with this ethical opinion. No amendment to protocol will be made without consideration and approval by the Trial Management Committee.

Feasibility data will be published as a peer-reviewed article and if successful, these findings will contribute to gaining further funding for a HTA full trial. In addition, assessing clinical data and blood derivatives from the participant cohort will provide valuable material (circulating tumour cells CTCs, transcript & plasma ctDNA), to validate translational studies of other AR aberrations and hormone targeting resistance pathways (or the emergence of biomarkers for chemo-sensitivity), to inform and contribute further to the rapidly evolving treatment developments for CRPC. Participants will remain anonymised in all publications.

We will also utilise dissemination through patient and professional organisations that have expressed their support for this trial (PCF, CRUK, NCRI Prostate CSG and BAUS) and through media outlets including web resources, lay press, academic national and international conferences and peer-reviewed journal publication.

Trial registration number: ISRCTN10246848

## Figure legends

Figure 1 VARIANT trial consort diagram. Planned flow of participants throughout the VARIANT study. mCRPC, Metastatic Castrate Resistant Prostate Cancer; PSA, Prostate Specific Antigen; EORTC-QLQ-C30/PR25, European Organisation for Research and Treatment of Cancer Quality of Life Questionnaire; AR-V7, Androgen Receptor Variant 7.

## References

1. Cancer Research UK (CRUK). Prostate Cancer Statistics. Electronic citation. [accessed 2019 August]. Available from: https://www.cancerresearchuk.org/health-professional/cancer-statistics/statistics-by-cancer-type/prostate-cancer.



For peer review only - http://bmjopen.bmj.com/site/about/guidelines.xhtml

2. Wu JN, Fish KM, Evans CP, Devere White RW, Dall'Era MA. No improvement noted in overall or cause-specific survival for men presenting with metastatic prostate cancer over a 20-year period. Cancer. 2014;120(6):818-23.

3. Public Health England. Cancer Information Tools. Electronic citation. [accessed 2019 Aug]. Available from: http://www.ncin.org.uk/cancer_information_tools .

4. Feldman BJ, Feldman D. The development of androgen-independent prostate cancer. Nat Rev Cancer. 2001;1(1):34-45.

5. James ND, Sydes MR, Clarke NW, Mason MD, Dearnaley DP, Spears MR, et al. Addition of docetaxel, zoledronic acid, or both to first-line long-term hormone therapy in prostate cancer (STAMPEDE): survival results from an adaptive, multiarm, multistage, platform randomised controlled trial. Lancet. 2016;387(10024):1163-77

6. Kyriakopoulos CE, Chen YH, Carducci MA, Liu G, Jarrard DF, Hahn NM, et al. Chemohormonal Therapy in Metastatic Hormone-Sensitive Prostate Cancer: Long-Term Survival Analysis of the Randomized Phase III E3805 CHAARTED Trial. J Clin Oncol. 2018;36(11):1080-87

7. James ND, de Bono JS, Spears MR, Clarke NW, Mason MD, Dearnaley DP, et al. Aberaterone for Prostate Cancer Not Previously Treated with Hormone Therapy. N Engl J Med. 2017;377(4):338-351

8. Fizazi K, Tran N, Fein L, Matsubara N, Rodriguez-Antolin A, Alekseev BY, et al. Aberaterone plus Prednisone in Metastatic, Castration-Sensitive Prostate Cancer. N Engl J Med. 2017;377(4):352-360

9. Davis ID, Martin AJ, Stockler MR, Begbie S, Chi KN, Chowdhury S, et al. Enzalutamide with Standard First-Line Therapy in Metastatic Prostate Cancer. N Engl J Med. 2019;381(2):121-31.

10. Chi KN, Agarwal N, Bjartell A, Chung BH, Pereira de Santana Gomes AJ, Given R, et al. Apalutamide for Metastatic, Castration-Sensitive Prostate Cancer. N Engl J Med. 2019;381(1):13-24.

11. Ross RW, Xie W, Regan MM, Pomerantz M, Nakabayashi M, Daskivich TJ, et al. Efficacy of androgen deprivation therapy (ADT) in patients with advanced prostate cancer: association between Gleason score, prostate-specific antigen level, and prior ADT exposure with duration of ADT effect. Cancer. 2008;112(6):1247-53.

12. Eisenberger MA, Blumenstein BA, Crawford ED, Miller G, McLeod DG, Loehrer PJ, et al. Bilateral orchiectomy with or without flutamide for metastatic prostate cancer. N Engl J Med. 1998;339(15):1036-42.

13. Crawford ED, Eisenberger MA, McLeod DG, Spaulding JT, Benson R, Dorr FA, et al. A controlled trial of leuprolide with and without flutamide in prostatic carcinoma. N Engl J Med. 1989;321(7):419-24.




For peer review only

For peer review only - http://bmjopen.bmj.com/site/about/guidelines.xhtml

14. ASCO. Practice Guidelines. https://wwwascoorg/practice-guidelines/qualityguidelines/guidelines/genitourinary-cancer. Accessed 13/10/2016.

15. Ryan CJ, Smith MR, de Bono JS, Molina A, Logothetis CJ, de Souza P, et al. Abiraterone in metastatic prostate cancer without previous chemotherapy. N Engl J Med. 2013;368(2):138-48.

16. Scher HI, Fizazi K, Saad F, Taplin ME, Sternberg CN, Miller K, et al. Increased survival with enzalutamide in prostate cancer after chemotherapy. N Engl J Med. 2012;367(13):1187-97.

17. Dehm SM, Tindall DJ. Alternatively spliced androgen receptor variants. Endocr Relat Cancer. 2011;18(5):R183-96.

18. Luo J. Development of AR-V7 as a putative treatment selection marker for metastatic castration-resistant prostate cancer. Asian J Androl. 2016;18(4):580-5.

19. Ware KE, Garcia-Blanco MA, Armstrong AJ, Dehm SM. Biologic and clinical significance of androgen receptor variants in castration resistant prostate cancer. Endocr Relat Cancer. 2014;21(4):T87-T103.

20. Beltran H, Antonarakis ES, Morris MJ, Attard G. Emerging Molecular Biomarkers in Advanced Prostate Cancer: Translation to the Clinic. Am Soc Clin Oncol Educ Book. 2016;35:131-41.

21. Maughan BL, Antonarakis ES. Clinical Relevance of Androgen Receptor Splice Variants in Castration-Resistant Prostate Cancer. Curr Treat Options Oncol. 2015;16(12):57.

22. Daniel M, Dehm SM. Lessons from tissue compartment-specific analysis of androgen receptor alterations in prostate cancer. J Steroid Biochem Mol Biol. 2016.

23. Stone L. Prostate cancer: AR-V7 status in CTCs is a treatment-specific biomarker. Nat Rev Urol. 2016;13(8):433.

24. Wadosky KM, Koochekpour S. Molecular mechanisms underlying resistance to androgen deprivation therapy in prostate cancer. Oncotarget. 2016.

25. Nakazawa M, Lu C, Chen Y, Paller CJ, Carducci MA, Eisenberger MA, et al. Serial blood-based analysis of AR-V7 in men with advanced prostate cancer. Ann Oncol. 2015;26(9):1859-65.

26. Steinestel J, Luedeke M, Arndt A, Schnoeller TJ, Lennerz JK, Wurm C, et al. Detecting predictive androgen receptor modifications in circulating prostate cancer cells. Oncotarget. 2015.

27. Scher HI, Lu D, Schreiber NA, Louw J, Graf RP, Vargas HA, et al. Association of AR-V7 on Circulating Tumor Cells as a Treatment-Specific Biomarker With Outcomes and Survival in Castration-Resistant Prostate Cancer. JAMA Oncol. 2016.



For peer review only - http://bmjopen.bmj.com/site/about/guidelines.xhtml

28. Hu R, Dunn TA, Wei S, Isharwal S, Veltri RW, Humphreys E, et al. Ligand-independent androgen receptor variants derived from splicing of cryptic exons signify hormone-refractory prostate cancer. Cancer Res. 2009;69(1):16-22.

29. Antonarakis ES, Lu C, Wang H, Luber B, Nakazawa M, Roeser JC, et al. AR-V7 and resistance to enzalutamide and abiraterone in prostate cancer. N Engl J Med. 2014;371(11):1028-38.

30. Armstrong AJ, Halabi S, Luo J, Nanus DM, Giannakakou P, Szmulewitz RZ, et al. Prospective Multicenter Validation of Androgen Receptor Splice Variant 7 and Hormone Therapy Resistance in High-Risk Castration-Resistant Prostate Cancer: The PROPHECY Study. J Clin Oncol. 2019;37(13):1120-9.

31. Antonarakis ES, Lu C, Luber B, Wang H, Chen Y, Nakazawa M, et al. Androgen Receptor Splice Variant 7 and Efficacy of Taxane Chemotherapy in Patients With Metastatic Castration-Resistant Prostate Cancer. JAMA Oncol. 2015;1(5):582-91.

32. Todenhofer T, Hennenlotter J, Feyerabend S, Aufderklamm S, Mischinger J, Kuhs U, et al. Preliminary experience on the use of the Adnatest(R) system for detection of circulating tumor cells in prostate cancer patients. Anticancer Res. 2012;32(8):3507-13.

33. Antonarakis ES, Lu C, Luber B, Wang H, Chen Y, Zhu Y, et al. Clinical Significance of Androgen Receptor Splice Variant-7 mRNA Detection in Circulating Tumor Cells of Men with Metastatic Castration-Resistance Prostate Cancer Treated with First- and Second-Line Abiraterone and Enzalutamide. J Clin Oncol. 2017;35(19):2149-56

34. Markowski MC, Frick KD, Eshleman JR, Luo J, Antonarakis ES. Cost-savings analysis of AR-V7 testing in patients with metastatic castration-resistant prostate cancer eligible for treatment with abiraterone or enzalutamide. Prostate. 2016.

35. Febbo PG, Ladanyi M, Aldape KD, De Marzo AM, Hammond ME, Hayes DF, et al. NCCN Task Force report: Evaluating the clinical utility of tumor markers in oncology. J Natl Compr Canc Netw.2011;9 Suppl 5:S1-32; quiz S3.

36. O'Connor JP, Aboagye EO, Adams JE, Aerts HJ, Barrington SF, Beer AJ, et al. Imaging biomarker roadmap for cancer studies. Nat Rev Clin Oncol. 2017;14(3):169-86.

37. Hornberg E, Ylitalo EB, Crnalic S, Antti H, Stattin P, Widmark A, et al. Expression of androgen receptor splice variants in prostate cancer bone metastases is associated with castration-resistance and short survival. PLoS One. 2011;6(4)

38. Antonarakis ES. Predicting treatment response in castration-resistant prostate cancer: could androgen receptor variant-7 hold the key? Expert Rev Anticancer Ther. 2015;15(2):143-5.

39. Antonarakis ES, Luo J. Prostate cancer: AR splice variant dimerization-clinical implications. Nat Rev Urol. 2015;12(8):431-3



For peer review only - http://bmjopen.bmj.com/site/about/guidelines.xhtml

40. Sieuwerts AM, Mostert B, van der Vlugt-Daane M, Kraan J, Beaufort CM, Van M, et al. An In-Depth Evaluation of the Validity and Logistics Surrounding the Testing of AR-V7 mRNA Expression in Circulating Tumor Cells. J Mol Diagn. 2018;20(3):316-25.

41. Hayes DF, Bast RC, Desch CE, Fritsche H, Jr., Kemeny NE, Jessup JM, et al. Tumor markerutility grading system: a framework to evaluate clinical utility of tumor markers. J Natl Cancer Inst. 1996;88(20):1456-66.

42. Simon RM, Paik S, Hayes DF. Use of archived specimens in evaluation of prognostic and predictive biomarkers. J Natl Cancer Inst. 2009;101(21):1446-52.

43. Sharp A, Porta N, Lambros MBK, Welti JC, Paschalis A, Raj GV, et al. Dissecting Prognostic From Predictive Utility: Circulating AR-V7 Biomarker Testing for Advanced Prostate Cancer. J Clin Oncol. 2019;37(24):2182-4.

44. De Laere B, Ost P, Gronberg H, Lindberg J. Has the PROPHECY of AR-V7 Been Fulfilled? J Clin Oncol. 2019;37(24):2181-2.

45. Dirix L. Predictive Significance of Androgen Receptor Splice Variant 7 in Patients With Metastatic Castration-Resistant Prostate Cancer: The PROPHECY Study. J Clin Oncol. 2019;37(24):2180-1.

46. Armstrong AJ, Halabi S, Luo J, Nanus DM, Scher HI, Antonarakis ES, et al. Reply to L. Dirix, B. De Laere et al, and A. Sharp et al. J Clin Oncol. 2019;37(24):2184-6

47. Attard G, Borre M, Gurney H, Loriot Y, Andresen C, Kalleda R, et al. ASCO 2017: Clinical factors associated with AR-V7 detection in ARMOR3-SV, a randomized trial of galeterone vs enzalutamide in men with AR-V7+ metastatic castration-resistant prostate cancer. Abstract 2017 ASCO Annual Meeting. June 2-6, 2017, Chicago.

48. Taplin ME, Antonarakis ES, Ferrante KJ, Horgan K, Blumenstein BA, Saad F, et al. Clinical factors associated with AR-V7 detection in ARMOR3-SV, a randomized trial of galeterone (Gal) vs enzalutamide (Enz) in men with AR-V7+ metastatic castration-resistant prostate cancer (mCRPC). J Clin Oncol. 2017;35:15_suppl, 5005-5005

49. de Wit R, de Bono J, Sternberg CN, Fizazi K, Tombal B, Wülfing C, et al. Cabazitaxel versus Abiraterone or Enzalutamide in Metastatic Prostate Cancer. N Engl J Med. 2019: Sep 30.

50. James ND, Spears MR, Clarke NW, Dearnaley DP, De Bono JS, Gale J, et al. Survival with Newly Diagnosed Metastatic Prostate Cancer in the "Docetaxel Era": Data from 917 Patients in the Control Arm of the STAMPEDE Trial (MRC PR08, CRUK/06/019). Eur Urol. 2015;67(6):1028-38

51. Grafa R, Hullings M, Barnett E, Carbone E, Dittamore R, Howard S. Clinical Utility of the Nuclear-localized AR-V7 Biomarker in Circulating Tumor Cells in Improving Physician Treatment Choice in Castration-resistant Prostate Cancer. Eur Urol. 2019: 2019: Oct 21.



For peer review only - http://bmjopen.bmj.com/site/about/guidelines.xhtml

52. Eldridge SM, Lancaster GA, Campbell MJ, Thabane L, Hopewell S, Coleman CL, et al. Defining Feasibility and Pilot Studies in Preparation for Randomised Controlled Trials: Development of a Conceptual Framework. PLoS One. 2016;11(3).

53. Teare MD, Dimairo M, Shephard N, Hayman A, Whitehead A, Walters SJ. Sample size requirements to estimate key design parameters from external pilot randomised controlled trials: a simulation study. Trials. 2014;15:264.

## Acknowledgments

We would like to acknowledge the valuable support of the VARIANT Trial Oversight Committee (TOC) members Dr Alison Tree, Dr Andrew Feber, Dr Rhian Gabe and our patient representatives on this committee, Robin Millman and Paul Nash. We are grateful for the assistance and support of our scientific collaborators Scott Dehm (University of Minnesota), Luke Gaughan (NICR), Jun Luo (John Hopkins), Gert Attard (UCL) and the All Wales Medical Genetics Laboratory (AWMGL).

Management of the study is by Newcastle University Clinical Trial Unit (NCTU).

## Authors' contribution

Emma Clark: Conception and design of the work, data collection, drafting of the article, critical revision of the article and final approval of the version to be published.

Miranda Morton: Trial management, data collection and drafting of the article.

Shriya Sharma: Trial management, data collection and drafting of the article.

Holly Fisher: Conception and design of the work, drafting of the article and critical revision of the article.

Denise Howel: Data collection, drafting of the article, critical revision of the article and final approval of the version to be published.

Jenn Walker: Trial management, data collection and drafting of the article.

Ruth Wood: Trial management, drafting of the article data collection, critical revision of the article and final approval of the version to be published

Helen Hancock: Critical revision of the article and final approval of the version to be published.

Rebecca Maier: Critical revision of the article and final approval of the version to be published.

John Marshall: Conception and design of the work, critical revision of the article and final approval of the version to be published.

Amit Bahl: Conception and design of the work.

Simon Crabb: Conception and design of the work and critical revision of the article.

Suneil Jain: Conception and design of the work and critical revision of the article.



For peer review only

Ian Pedley: Conception and design of the work.

Rob Jones: Conception and design of the work, data collection, drafting of the article, critical revision of the article and final approval of the version to be published.

John Staffurth: Conception and design of the work, data collection, drafting of the article, critical revision of the article and final approval of the version to be published.

Rakesh Heer: Conception and design of the work, drafting of the article, critical revision of the article and final approval of the version to be published.

**Funding statement**

This work was supported by a National Institute for Health Research (NIHR) Research for Patient Benefit (grant number PB-PG-0816-20043). The VARIANT trial is sponsored by Newcastle upon Tyne Hospitals National Health Service Foundation Trust.

**Competing Interests Statement**

Dr. Jain reports personal fees from Astellas, personal fees from Bayer, personal fees from Janssen, personal fees from Boston Scientific, personal fees from Almac Diagnostics, personal fees from Sanofi Genzyme, personal fees from Movember, outside the submitted work; Dr. Bahl reports research funding and advisory roles with Sanofi and Janssen and an advisory role with Astellas and Bayer, outside the submitted work. Dr. Jones reports grants and personal fees from Astellas, grants and personal fees from AstraZeneca, personal fees and non-financial support from Bristol Myers Squibb, grants, personal fees and non-financial support from Bayer, grants and personal fees from Exelixis, personal fees and non-financial support from Janssen, personal fees and non-financial support from Ipsen, personal fees from Merck Serono, personal fees and non-financial support from MSD, personal fees from Novartis, personal fees from Pfizer, grants and personal fees from Roche, personal fees from Sanofi Genzyme, personal fees from EUSA, outside the submitted work; .Prof. Staffurth reports non-financial support from Bayer and personal fees from Janssen and Astellas outside of the submitted work. Dr. Crabb has an honoraria/advisory role with Roche, Clovis Oncology, Bayer, Janssen Cilag and Merck and receives research support from AstraZeneca, Astex Pharmaceuticals and Clovis Oncology.

**Word Count (excluding title page, tables and reference list): 4, 446**



For peer review only - http://bmjopen.bmj.com/site/about/guidelines.xhtml

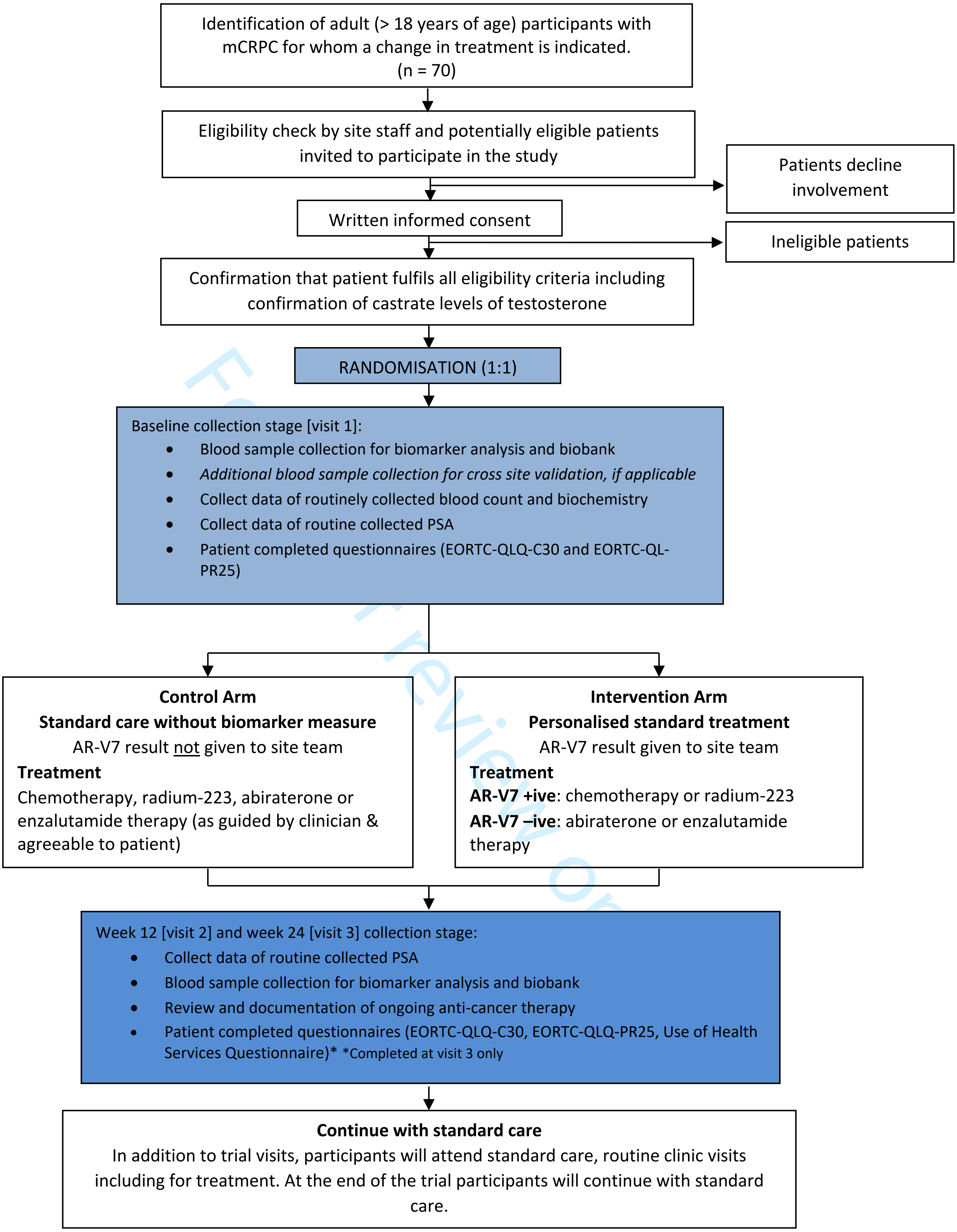


**Figure 1** VARIANT trial consort diagram. Planned flow of participants throughout the VARIANT study. mCRPC, Metastatic Castrate Resistant Prostate Cancer; PSA, Prostate Specific Antigen; EORTC-QLQ-C30/PR25, European Organisation for Research and Treatment of Cancer Quality of Life Questionnaire; AR-V7, Androgen Receptor Variant 7.

For peer review only - http://bmjopen.bmj.com/site/about/guidelines.xhtml

The constitution of the Trial Management Group (TMG): Chief Investigator(s), Sponsor representative, Co-Lead Investigator, Laboratory and Translational Science Lead, Statistician Lead, Trial Management Team, Co-Applicants and Collaborators who will attend TMG meetings as required.

The constitution of the Trial Oversight Committee (TOC) as a combined TOC whose members are independent of the trial: independent Chair, Independent Lab representative, Independent statistician and two independent patient representatives.

**Roles and Responsibilities**

Chief Investigator - Dr Rakesh Heer, Senior Lecturer and Consultant Urological Surgeon, Translational and Clinical Research Institute, Faculty of Medical Sciences, Newcastle University

Co-Lead Chief Investigator - Prof. John Staffurth, Professor in Oncology and Consultant Oncologist, Velindre Cancer Centre, Cardiff University

Laboratory and Translational Science Lead - Dr Emma Clark, Translational Research Associate, Translational and Clinical Research Institute, Faculty of Medical Sciences, Newcastle University

Principle Investigator - Prof Rob Jones, Professor of Clinical Cancer Research/ Honorary Consultant in Medical Oncology, University of Glasgow

Principle Investigator - Dr Ian Pedley, Clinical Director of NCCC and Clinical Oncologist, The Newcastle upon Tyne Hospitals NHS Foundation Trust

Co-applicants – [1] Dr Amit Bahl, Senior Lecturer, Consultant Oncologist and Clinical Director, University Hospitals Bristol NHS Foundation Trust; [2] Dr Simon Crabb, Associate Professor and Honorary Consultant in Medical Oncology, University of Southampton; [3] Dr Suneil Jain, Senior Lecturer and Consultant in Clinical Oncology, Queen's University Belfast.

PPI Representative - Dr John Marshall

Senior Statistician - Denise Howel, Population Health Sciences Institute, Faculty of Medical Sciences, Newcastle University

Statistician - Dr Holly Fisher, Population Health Sciences Institute, Faculty of Medical Sciences,, Newcastle University

Data Manager – Ruth Wood, Newcastle Clinical Trials Unit, Newcastle University

Senior Trial Manager – Jenn Walker, Newcastle Clinical Trials Unit, Newcastle University



For peer review only - http://bmjopen.bmj.com/site/about/guidelines.xhtml

Joint Trial Manager - Dr Miranda Morton and Shriya Sharma, Newcastle Clinical Trials Unit, Newcastle University

Sponsor - The Newcastle upon Tyne Hospitals NHS Foundation Trust

Funder - National Institute for Health Research – Research for Patient Benefit

Out of Hours Contact - Dr Rakesh Heer, Newcastle University

Trial Oversight Committee (TOC) Chair - Dr Alison Tree, Uro-oncology Trials Team Leader and Consultant Clinical Oncologist, The Institute of Cancer Research

## Recruitment and screening

Patients will be approached during routine clinic appointments from urology or oncology clinical services. Potentially eligible patients will have the trial explained to them, provided with a Patient Information Sheet (PIS) and their medical notes reviewed to establish if they are likely to be eligible to take part in the trial. In addition to assessing patient eligibility against the inclusion and exclusion criteria, a complete medical history including the patient's age and detailed information about their prostate cancer history and metastases will be collected after consent.

## Consent Procedure

Full written informed consent will be received by signing, dating and initialling the consent form, which will be witnessed by a member of the research team, who has documented and delegated responsibility, and will and check eligibility and counter-sign the consent form. The participant will specifically consent to; [1] their GP being contacted and informed of participation in the study; [2] access to relevant sections of their medical notes to carry out follow-up after the trial has ended; and [3] serial collection of blood samples for biomarker testing and storage in the Androgen Receptor Biology Bio-Bank (AR-3B) biobank for up to 10 years after the trial has ended.

## Data collection methods

In addition to collecting standard care assessment of disease status data from intervention and control groups, trial specific questionnaire assessment (EORTC QLQ-C30 Quality of Life of Cancer Patients, EORTC QLQ-PR25 Quality of Life Prostate Cancer Module) and blood sample collection will take place at baseline, 12 and 24 week visits. Use of Health Services Questionnaire will be completed end of trial assessments. Participant data will only be identified using a unique individual participant identifier.

**Standard care assessments:**

The following clinical assessments will be conducted;

1. Cause of death (if appropriate)



For peer review only - http://bmjopen.bmj.com/site/about/guidelines.xhtml

2. Evidence of PSA progression (>25% and >2 ng/mL above the nadir and confirmed by a second value >3 weeks later)
3. Clinical evidence of progression, stage and type of progression (biochemical, radiological or symptomatic)
4. Result of routinely collected PSA and testosterone measurements, full blood count and biochemistry tests
5. Details of anti-cancer therapy, including dates of treatments for ongoing anti-cancer therapy.

**Questionnaires:**

The EORTC quality of life questionnaire is an integrated system for assessing the health-related quality of life (QOL) of cancer patients participating in clinical trials. There is a set of core questions (QLQ-C30), supplemented by a prostate cancer specific module (PR25). PR25 is a diagnosis-specific module designed to be used in conjunction with the QLQ-C30, it is intended for use among a wide range of patients with prostate cancer varying in disease stage and treatment modality.

The QLQ-C30 consists of thirty questions incorporating; [1] functional scales, symptom scales and a number of items assessing additional symptoms commonly reported by patients with cancer, assessed on a four point scale and [2] a global health status/quality of life scale, assessed on a seven point scale. The PR25 module consists of 25 questions incorporating functional and symptom scales, all assessed on four point scale. The Use of Health Services questionnaire consists of ten questions assessing participant's use of health services over the course of their participation in the trial.

## Data Management and Archiving

Data including the number of patients screened, approached and interested in taking part will be collected via a screening log. Trial and screening data is collected on electronic case report forms (eCRFs) using password limited access, secure web-based interface for data entry with inbuilt back-up facility, will be managed using a Clinical Data Management System (Elsevier's MACRO™) overseen by the Newcastle Clinical Trials Unit (NCTU). Individual access will be limited according to delegated roles and duties. Data will be handled, computerised and stored in accordance with the UK Data Protection Act 2018. All trial data will be retained in accordance with the latest Directive on GCP (2005/28/EC).

Participant clinical information will not be released without the written permission of the participant, except as necessary for monitoring and auditing by the Sponsor, its designee, Regulatory Authorities, the Trial oversight committee (TOC) or the Research Ethics Committee (REC). Trial data will be released to the trial statistician for analysis, to Paul O'Gorman Newcastle University biobank researchers after trial analysis and used in planning any future, definitive trial. All trial data will be stored for 10 years, in accordance with GCP and sponsor approved SOPs.



For peer review only - http://bmjopen.bmj.com/site/about/guidelines.xhtml

## AR-V7 biomarker driven personalised treatment pipeline

Biomarker driven personalised treatment will be based on current scientific evidence from the Adnatest assay biomarker founding lab (John Hopkins, Baltimore, USA). A biomarker positive result is when a read is detected at 35 qPCR cycles or less and the recommendation will be to proceed with chemotherapy. A biomarker negative result is when a read is detected over 35 qPCR cycles (or there is no read at any qPCR cycle), and the recommendation will be to proceed with hormonal therapy (Enzalutamide or Abiraterone).

Participants will be required to give 30ml of blood at baseline (0 weeks), 12 weeks and 24 weeks. Samples will be routinely processed for biomarker read-out and AR-3B biobank storage at the Newcastle University central analysis lab as detailed in the VARIANT biomarker flow chart below. Following biomarker data analysis and data verification (according to sponsor agreed analytical and validation plan and SOPs), for participants randomised to the intervention standard treatment arm (AR-V7 biomarker guided), the baseline biomarker result and biomarker treatment recommendation will be sent securely within 10 working days to the local PI and delegated research staff. The PI and local trial team will then communicate with the participant regarding the biomarker guided treatment. Any modifications to the recommended treatment for participants will be made at the discretion of the treating clinician, based on their clinical judgement. Biomarker results from blood samples taken at weeks 12 and 24 will not be made available to the PI, local trial team or participant.

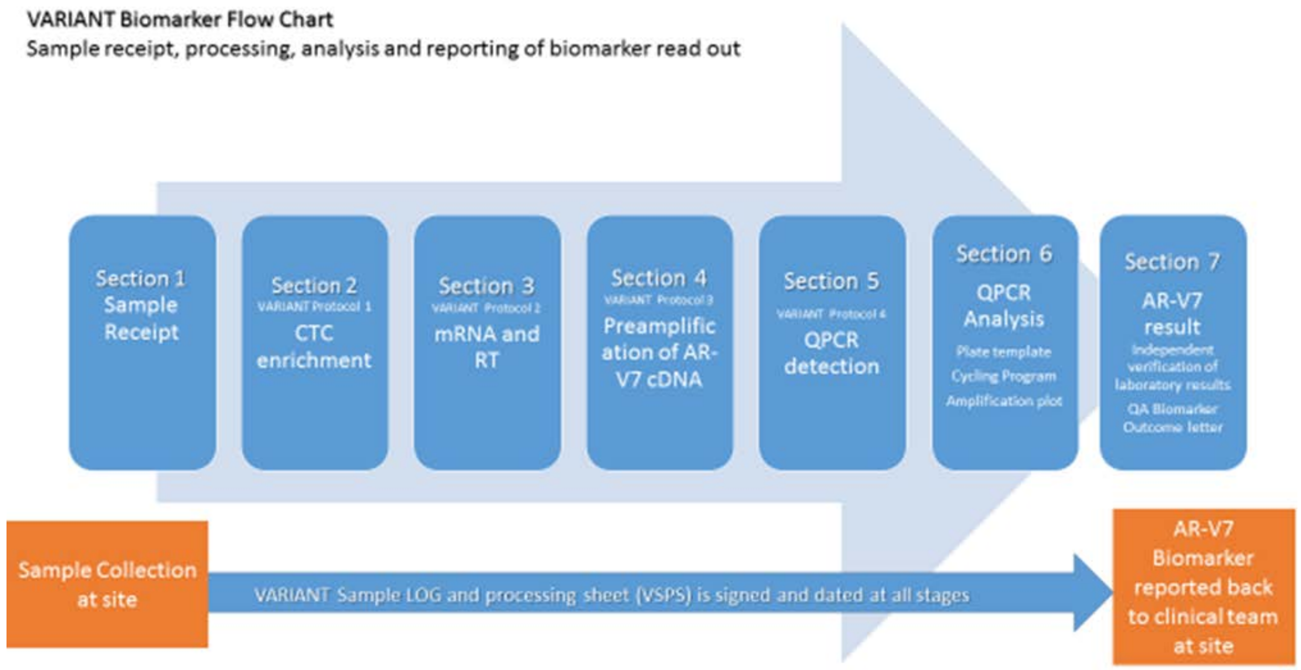


For participants randomised to the standard treatment arm (not AR-V7 biomarker guided), biomarker results will not be made available to the PI, local trial team or participant for any of the blood samples analysed (baseline, week 12 and week 24).



For peer review only - http://bmjopen.bmj.com/site/about/guidelines.xhtml

**AR-V7 biomarker cross-site validation**

An additional twenty participants selected at randomisation will provide a further (10ml) blood sample for cross site validation analysis of the biomarker at the Cardiff Central Analysis Lab using the commercially available AdnaTest ProstateCancerPanel AR-V7 circulating tumour cell (CTC) quantitative RT-PCR (RT-qPCR) assay (Qiagen®).

**Androgen Receptor Biology driving hormone resistance**

Research suggests that key 'sub-populations' or clones of molecular alterations (of which AR-V7 is an important subtype), compete with each other and are drivers of treatment resistance. In addition to performing AR-V7 biomarker assay, a fuller capture of AR-related CRPC biology will be achieved by collecting CTCs, plasma, buffy coat and red blood cell derivatives to compile an Androgen Receptor Biology Bio-Bank (AR-3B). Whole and CTC depleted blood sample derivatives will be collected and biobanked at 12 weekly intervals from baseline (prior to treatment) throughout follow up (to a maximum of 24 weeks +/- 2 weeks), including all trial captured data.

It is also important to acknowledge the expression of other AR splice variants are also associated with resistance to hormonal treatment(1,2), as are other AR pathway alterations such as AR mutations and amplifications(3). Ongoing discussions in the field as to the discriminatory value of solely detecting AR-V7 expression, may ultimately lead to a combination test that will improve treatment stratification and patient outcomes(4). However, at this time, AR-V7 remains the forerunner and a formal understanding of biomarker characteristics in the advanced prostate cancer treatment setting is required 'to best inform' a formal large-scale testing.

The AR-3B biorepository resource will be used to assess total VARIANT expression (capturing all VARIANTs in a single qPCR reaction) and explore (but not be limited to) AR hot spot mutations/sequencing/amplification and other mutations such as PTEN/p53/MYC gain/RB1 loss/MET gain/PARPi and further molecular pathways based on yet to be defined but new emerging data in time, in blood and blood derivatives.

This resource will provide added value to the feasibility study, by banking processed biomarker tissue for additional biomarker measures that may also contribute to hormone targeting resistance (or the emergence of biomarkers for chemo-sensitivity), and importantly be relevant in prostate cancer management. Blood samples will be transported, stored, accessed and processed in accordance with sponsor approved SOPs following appropriate legislation relating to the use and storage of human tissue for research purposes and such activities shall at least meet the requirements as set out in the 2004 Human Tissue Act and 2006 Human Tissue (Scotland) Act.

The results of any other research utilising blood samples (including those in the control arm), will not be reported back to the clinical team or to participants, this data is for research purposes only and will be published in appropriate peer reviewed scientific journals. Participants will remain anonymised in all publications.



For peer review only - http://bmjopen.bmj.com/site/about/guidelines.xhtml

## Androgen Receptor Biology Bio-Bank (AR-3B) sample storage governance

Samples will be appropriately labelled in accordance with trial protocol as described in the current VARIANT trial site blood collection manual (Version 1.0 28th March 2019) to comply with the General Data Protection Regulation (GDPR) and Data Protection Act (GPA) 2018 and pseudo-anonymised with linkage to participant details possible only through the Participant Identification Log assessable only to delegated personnel. Sample shipment will be tracked and sample receipt at central analysis labs recorded using a specific sample collection MACRO™ database which is separate to the main trial database.

All AR-3B biobank samples are stored under HTA license 12534, under the Designated Individual, Dr. Christopher Morris (NICR). All samples are held under the custodianship of Mr. Rakesh Heer (Chief Investigator), who is responsible for the curation of these samples and ensuring compliance with the Human Tissue Act (2004) and GCP. Samples will be tracked and stored using Achiever medical LIMS.

Blood samples sent to the Cardiff Central Analysis Lab, will be consumed within 7 days of receipt and are not under the remit of the HTA. All RNA and DNA derivatives will be transferred to the Newcastle University AR-3B biorepository for storage at the end of the trial.

Supplementary Section References:

1. Van Etten JL, Nyguist M, Li Y, Yang R, Ho Y, Johnson R, et al. Targeting a Single Alternative Polyadenylation Site Coordinately Blocks Expression of Androgen Receptor mRNA Splice Variants in Prostate Cancer. Cancer Res. 2017;77(19):5228-35

2. Henzler C, Li Y, Yang R, McBride T, Ho Y, Sprenger C, et al. Truncation and constitutive activation of the androgen receptor by diverse genomic rearrangements in prostate cancer. Nat Commun. 2016;7:13668

3. De Laere B, van Dam PJ, Whitington T, Mayrhofer M, Diaz EH, Van den Eynden G, et al. Comprehensive Profiling of the Androgen Receptor in Liquid Biopsies from Castration-resistant Prostate Cancer Reveals Novel Intra-AR Structural Variation and Splice Variant Expression Patterns. Eur Urol. 2017;72(2):192-200

4. Zhang J, Cunnigham JJ, Brown JS and Gatenby RA. Integrating evolutionary dynamics into treatment of metastatic castrate-resistant prostate cancer. Nat Commun. 2017;8(1):1861



For peer review only - http://bmjopen.bmj.com/site/about/guidelines.xhtml

To be printed on local headed paper

# PATIENT INFORMATION SHEET

# The Variant 7 Biomarker Feasibility Study

## The **VARIANT** Study

### We are inviting you to take part in a research study

Please read the following information to help you decide if you want to take part. It will explain why we are doing this research and what it might mean for you. You are free to decide whether or not to take part in this study. You do not have to decide straight away and you can talk to your friends/family about it. Ask us if you have any questions or you want to know more. If you choose not to take part, this will not affect the care you get from your doctors.

### Study Summary

- In this study we will be taking 3 blood samples and asking patients to complete some questionnaires during their usual hospital appointments.
- Patients with **advanced metastatic prostate cancer** (cancer that has spread from the prostate to other parts of the body), often eventually stop responding to initial hormone therapy. After this point, treatment is usually with either:
    1) **Further hormonal treatment called next generation hormone treatment OR**
    2) **Non-hormonal treatment such as chemotherapy or radiotherapy**
- There is currently no clear guidance for which of these two different standard care treatment options a patient should receive.
- Testing the level of AR-V7 (a type of protein) in blood could suggest which of these two treatments patients will respond to best. The AR-V7 test is known as a biomarker test.
- VARIANT is looking to find out if the AR-V7 biomarker test is helpful to support doctors and patients in choosing between these treatment options.
- In this study, half of patients will receive treatment guided by the AR-V7 biomarker test. The other half will receive treatment as usual.

**Please read the following information to see if you may be interested in taking part.**



For peer review only - http://bmjopen.bmj.com/site/about/guidelines.xhtml

For peer review only

## What is a biomarker test?

Biomarkers are substances that can be found and measured in parts of the body, in this case, the blood. Biomarker testing is a type of test that looks for these substances to give doctors information about a patient's health. **The AR-V7 blood test is a biomarker test looking for the AR-V7 protein.**

## Why is VARIANT needed?

Initial treatments for advanced metastatic prostate cancer include hormone treatment alone or chemotherapy in combination with hormone treatment. Eventually most patients with advanced prostate cancer stop responding to initial hormone treatment. At this point, patients usually receive treatment with either further hormone treatment, known as next generation hormone treatment (such as abiraterone or enzalutamide), or with non-hormonal options (typically chemotherapy, or in some cases radium-223 radiotherapy). Although there are fewer side effects associated with next generation hormone treatment relative to chemotherapy, only 30-50% of patients will respond well to further hormone treatment.

For peer review only

The **AR-V7 biomarker is found in the blood** of some men who have received initial hormone treatment for prostate cancer. Recent studies have suggested that patients who have this biomarker in their blood may be less likely to respond well to advanced hormone therapy. Measuring the amount of this biomarker in blood (which is not usually tested for), may be useful to help guide choice of treatment for patients with advanced metastatic prostate cancer. We hope this will improve patient experience and outcome by spending less time on and experiencing side effects of treatments that might not work, starting a different treatment earlier, and potentially reduce the cost to the NHS.

The VARIANT study is a feasibility study in which we will look at whether doctors and patients are willing to use the results of this blood test to decide on a treatment option. At this stage we do not know that the AR-V7 biomarker will lead to patients having better responses to treatments. However, we hope that the results of VARIANT will help us to plan a similar, but larger, study to find out if the AR-V7 blood test does result in better outcomes for patients and whether AR-V7 testing should be used in standard NHS practice. We hope that 70 patients will take part in VARIANT.

## Why have I been invited to take part?

You have been diagnosed with advanced metastatic prostate cancer and have already been treated with hormone therapy (also known as androgen deprivation therapy, or ADT). Your disease has stopped responding to the current therapy and you are due a change in treatment plan.

## Do I have to take part?

No, it is up to you to decide if you want to take part in VARIANT. If you do not want to take part, you will still get the standard treatment that has been arranged by your treating doctor.

For peer review only - http://bmjopen.bmj.com/site/about/guidelines.xhtml

If you agree to take part, you can change your mind and withdraw from the study at any time without having to give a reason.

## What does taking part involve?

- If you decide to take part, you will be selected to have either:
  - **a) treatment guided by AR-V7 blood test result**

    In this group, your treating doctor will receive the results of your AR-V7 blood test. Your treating doctor will tell you the result of the AR-V7 blood test and discuss this with you before arranging a treatment option for you. The results of the test may support you and your doctor in deciding whether next generation hormonal therapy or non-hormonal therapy would be more suitable for you.
  - **b) treatment as usual**

    In this group, your doctor will arrange a treatment option with you as they usually would if you were not in the study. You and your treating doctor will NOT receive the results of your AR-V7 blood test. The results will only be looked at by the trial management team at the end of the study.
- You will have equal chance of being in group a) or group b) (a 50:50 chance). Your group will be selected by a computer. We call this ‘randomisation’. Your doctor will not have any say over which group you are in.
- It is important to note that we are not testing a new treatment in this study. All VARIANT patients will receive one of the usual treatment options available to patients in the NHS. The study is looking at whether doctors and patients are willing to use the AR-V7 blood test and whether it is helpful in deciding **which** treatment option is most suitable for individual patients.

## What will I have to do?

A member of the VARIANT team will discuss the study with you and answer any questions you may have. If you decide to take part, and your doctor confirms you are eligible for the study, you will be asked to sign a consent form.

As well as receiving the treatment arranged with your doctor, taking part in the study will mean (for ALL patients, that is patients in group a) and group b)):

- You will be asked to give a **blood sample on 3 occasions** (at the start of the study, after 12 weeks and 24 weeks). We would like to collect around 20 ml of blood (about 4 teaspoons full) each time.
- Some patients will be selected to give an **additional blood samples on one occasion** (at the start of the study). We would like to collect around 10 ml of blood (about 2 teaspoons full) for this sample. Patients will be randomly selected by a computer system to give this additional blood samples. The doctor or a member of their team will tell you if you have been selected to give this sample.


For peer review only - http://bmjopen.bmj.com/site/about/guidelines.xhtml

- On 2 of these visits, you will be asked to complete 2 **short questionnaires** about your quality of life. These will take around 15 minutes each to complete.
- Study visits will all take place during your usual hospital visits. There should be no extra visits to hospital required.
- We will use some information that is already collected about you as part of your standard clinical care. This includes information about your diagnosis, your treatment, results of scans and blood tests and your physical health. Taking part in VARIANT does not involve any extra scans to those you would receive normally.
- To take part in VARIANT, you will be asked to give your permission for your blood samples collected during this study to be stored at the Northern Institute of Cancer Research and used for future research as described below.

## Study timeline in addition to standard treatment

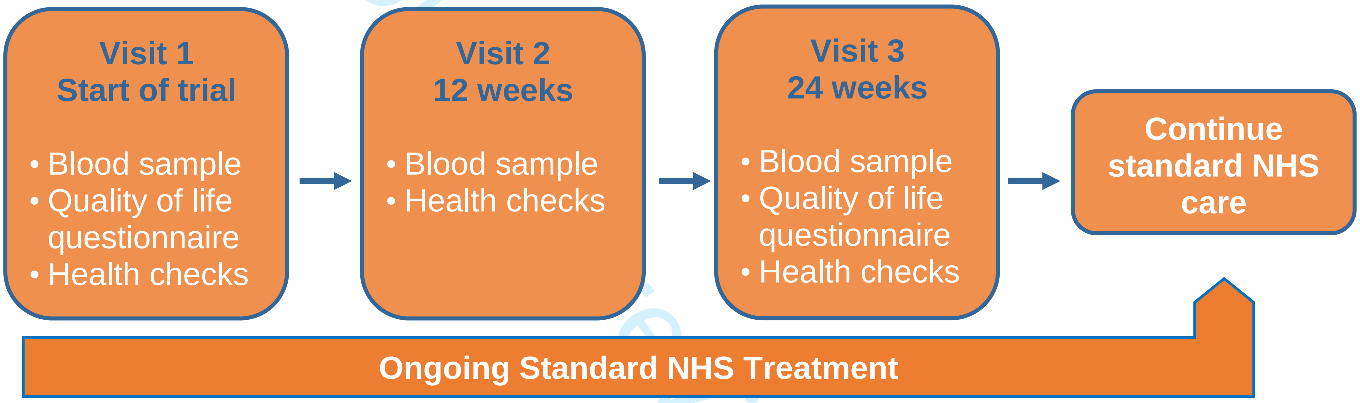


## What will happen to my blood samples?

Your blood samples will be tested for the AR-V7 biomarker at the Northern Institute for Cancer Research (NICR). If you are selected to give the additional blood sample this will be sent to the All Wales Medical Genetics Laboratory (AWMGL) for testing. You and your treating doctor will only find out the AR-V7 biomarker result if you are selected to have your treatment guided by the AR-V7 blood test result.

After the AR-V7 test, any remaining sample will be stored at the NICR biobank (a collection of samples) and will be used in future research to look at why people develop advanced prostate cancer and how cancer reacts to treatments. If you do not want your samples to be stored or used through the NICR biobank you will not be able to take part in this trial.

Your samples will be labelled with a unique study number. This number is used to link your sample back to you and only the study team and authorised people will be able to do this. Your sample will be sent with a form that includes your initials, date of birth and the date the sample was taken. This will help us make sure the samples can be linked back to the correct person.

For more detailed information about what will happen with your blood samples please see the section ‘Further information about what will happen to blood samples’ on page X, we recommend that you read this.


For peer review only - http://bmjopen.bmj.com/site/about/guidelines.xhtml

## Expenses and payments

You will not receive payment for taking part in VARIANT. All parts of the study will happen when you are already coming to hospital for your usual visits.

## What happens when the research study stops?

Your appointment at 24 weeks will be the last time we see you for the study. At the end of the study you will continue to receive usual clinical care as decided by your hospital and doctor. If appropriate, this may include continuing the anti-cancer drugs you are receiving, or changing to another if you and your doctor believe this to be in your best interests.

## What are the possible risks and benefits of taking part?

**Risks**: Taking blood samples may cause some discomfort and minor pain, and occasionally patients can feel faint during or after. Sometimes patients will have some bruising where the blood has been taken. Only trained members of staff will perform the blood tests and every effort will be made to prevent any discomfort.

VARIANT patients will receive one of the usual treatment options available to patients in the NHS. Your doctor will be able to tell you more about possible side effects of these treatments. The risk of these treatments is the same as if you were not taking part in the study.

**Benefits**: By taking part in VARIANT, you will be helping us gather information to learn about using the AR-V7 biomarker to guide treatment for patients with advanced metastatic prostate cancer. We hope that we can improve the quality of life of patients in the future from VARIANT.

For some patients, taking part in the study will mean that your doctor receives your AR-V7 biomarker result before deciding on a treatment for you. This may help to inform which treatment would be best for you after reviewing your medical history.

If you want to find out more about taking part in research studies, you can visit the NHS Choices Website www.nhs.uk/conditions/clinical-trials/. On this website you will also find contact details for your local Patient Advice Liaison Service (PALS) office if you would like to speak to someone.

For peer review only

# Further supporting information

## What will happen if I do not want to carry on in the study?

You can withdraw your consent at any time and for any reason, without having to tell us your reason. You will be fully cared for and supported as per your hospital's standard practice.

We will ask if you are happy for us to:

- Use any information already collected about you.
- Continue using information collected as part of your usual care until the end of the study.


For peer review only - http://bmjopen.bmj.com/site/about/guidelines.xhtml

You can change your mind about allowing your stored blood samples to be used for research at any time in the future, without giving any reasons, by contacting your local study team as listed on page x. Any samples left at the NICR will be destroyed. Any researchers to whom samples have been sent will be instructed to destroy the samples they have in their laboratories. It will not be possible to withdraw any findings from research work already undertaken using your donated samples.

## Can I take part in other research?

If you are already taking part in a clinical trial we would look to see what the trial involved and speak to the other trials team. We would need check that taking part in one trial would not affect the treatment or results in the other.

If you take part in VARIANT and want to join another trial in the future, we ask that you let the VARIANT team know so we can check with their team that there is no conflict. If there was a conflict, the study team would discuss your options with you and you can decide what it best for you.

## What if there is a problem?

If you are not happy with any part of VARIANT, you should ask to speak to the study team first who will do their best to help you. **Their contact details are on page X.** If you are still unhappy you may wish to raise your concerns with someone who is not directly involved in your care. You can contact the Patient Advice Liaison Service (PALS) who provide a confidential service on <site to localise with phone number and email address>

In the unlikely event that you are harmed during the research and this is due to someone's negligence (they were careless) you may have grounds for legal action for compensation, but you may need to meet your own legal costs. NHS indemnity does not offer no-fault compensation (for harm that is not anyone's fault).

## Will my GP be told about my involvement in VARIANT?

Yes, with your permission we will inform your GP that you are taking part in VARIANT. We will send you a copy of this letter so that you can see exactly what has been said. It will also be noted in your hospital medical records so that staff in the hospital know you took part in the study.

## What will happen to the results of the study?

- The study is due to finish at the end of 2020.
- The results will be written in medical journals and presented in meetings to other doctors, nurses and researchers.
- The anonymised data might be shared with other researchers and to help with future studies. Your identity will always be protected.
- A report will be written by the study funder and put on their website.

For peer review only


For peer review only - http://bmjopen.bmj.com/site/about/guidelines.xhtml

- You may request a summary of the results at the end of the trial by contacting the study team, their details are on page X.

## Will the information about me be kept confidential?

Yes. All of the information collected will be entered on computers that are kept secure and password protected.

- We will use a study number to identify you instead of using your name.
- Your contact details will never be shared with anyone else.
- You will not be named in any published results, reports or anything on our website.

The study information about you and your medical notes will be looked at by people directly involved in the study, clinical and lab staff as well as by people who are checking the study is running as it should. This may include the Newcastle Clinical Trials Unit at Newcastle University as they are managing the study. It may also include regulatory authorities, sponsor and funder.

Your local study team will collect information from you during the study. This will be entered onto a secure database using your study number instead of your name. Your local study team will be the only people that know that the study number refers to you. This means that the data on the database it is not identifiable to anyone outside of your local study team. The database is held by the Newcastle Clinical Trials Unit at Newcastle University. Access to this database is password protected and available to your doctors and research staff for the purpose of the trial. Data from the database will be analysed at the end of the whole study. Anonymised data from the database may be used when applying to carry out future related clinical trials or for other research projects related to prostate cancer.

After you have finished attending study visits, your local study team may review your health records and collect information for the study.

## Data Protection

All of the data we collect will be kept strictly confidential and in accordance with the General Data Protection Regulation (GDPR).

We have included information on page X of this document that tells you how we do this and included some websites where you can find out more.

## Who is organising and funding VARIANT?

The main study doctor (also called the ‘Chief Investigator’) is Mr Rakesh Heer, a Urological Surgeon at The Newcastle upon Tyne Hospitals NHS Foundation Trust. The study team includes senior doctors and nurses, university experts in research studies, and members of the public.

It is managed by the Newcastle University Clinical Trials Unit on behalf of the study sponsor – The Newcastle upon Tyne Hospitals NHS Foundation Trust. It is funded by the National Institute for Health Research, Research for Patient Benefit programme.



For peer review only - http://bmjopen.bmj.com/site/about/guidelines.xhtml

At least 3 hospitals will be taking part in VARIANT. Each hospital has a study doctor, called an 'Investigator'. The Investigator in your hospital is …………………………..

## Who has reviewed the study?

All research in the NHS is looked at by an independent group of people called a Research Ethics Committee. VARIANT has been reviewed and given a favourable opinion by the Wales REC 2 committee.

Patients have been involved in deciding how to do VARIANT from the start. For example, patients were involved in designing and applying for funding for the study, and continue to contribute as part of the study team. We also asked a group of patients and carers who have experienced cancer to look at the study information sheet to check the study is described in a clear way and is easy to understand.

For peer review only

## What if relevant new information becomes available?

The treatment management of metastatic prostate cancer is changing all of the time. All VARIANT patients will receive one of the standard treatment options available in the NHS. This will include any new treatment options that become available while the study takes place.

Information gathered during the course of this study will be reviewed by an independent Trial Oversight Committee. The role of this group is to protect the safety and wellbeing of participants by making sure the study is running safely.

## What if I have any questions?

Please ask the doctor or nurse who is looking after you. They can put you in touch with the research team or the Investigator for VARIANT at your hospital.

## What happens next?

You can take time to think about the study and whether you want to take part. A member of the research team will speak to you when you come back in to discuss your treatment options. They will go through this information sheet with you and answer any questions before you make your final decision.

## Thank you for taking the time to read this information sheet.

**VARIANT team contact details for your hospital:**

| | |
|---|---|
| **Principal Investigator:** | **Research Nurse:** |
| **Address:** | **Address:** |
| **Tel:** | **Tel:** |



For peer review only - http://bmjopen.bmj.com/site/about/guidelines.xhtml

## GDPR and Transparency Information

The Newcastle upon Tyne Hospitals NHS Foundation Trust (NUTH) is the sponsor for this study based in the United Kingdom and will act as the "data controller" for this study. **They are responsible for looking after your information and using it properly.**

This study is managed on behalf of the sponsor by the Newcastle Clinical Trials Unit who will act as the "data processor". As data processor, this means that we are responsible for processing personal data on behalf of a controller. We will be using information from you in order to undertake this study, and will keep identifiable information about you for 10 years.

Your rights to access, change or move your information are limited, as we need to manage your information in specific ways in order for the research to be reliable and accurate. If you withdraw from the study, we will keep the information about you that we have already obtained. To safeguard your rights, we will use the least amount of personally-identifiable information possible.

You can find out more about how your information is used at http://www.newcastle-hospitals.org.uk/about-us/freedom-of-information_how-we-use-information

To find out more about research and general use of patient information please refer to the Health Research Authority Website https://www.hra.nhs.uk/information-about-patients/

The sponsor as an NHS Organisation and the Newcastle Clinical Trials Unit as a University use personally-identifiable information to conduct research to improve health, care and services. As a publicly-funded organisation, we have to ensure that it is in the public interest when we use personally-identifiable information from people who have agreed to take part in research. This means that when you agree to take part in a research study, we will use your data in the ways needed to conduct and analyse the research study.

Health and care research should serve the public interest, which means that we have to demonstrate that our research serves the interests of society as a whole. We do this by following the UK Policy Framework for Health and Social Care Research.

If you wish to raise a complaint on how we have handled your personal data, you can contact our Data Protection Officer who will investigate the matter. If you are not satisfied with our response or believe we are processing your personal data in a way that is not lawful you can complain to the Information Commissioner's Office (ICO).

The sponsor Data Protection Officer is Richard Oliver and you can contact them at nuth.dpo@nhs.net.

The local study team at your hospital will collect information from you and/or your medical records for this research study in accordance with our instructions.

The local study team will use your name, NHS number and contacts details to contact you about the research study, and make sure that relevant information about the study is recorded for your care, and to oversee the quality of the study. Individuals from the sponsor, Newcastle Clinical Trials Unit and regulatory organisations may look at your medical and research records to check the accuracy of the research study. The local



For peer review only - http://bmjopen.bmj.com/site/about/guidelines.xhtml

study team will pass these details to the sponsor or the Newcastle Clinical Trials Unit along with information collected from you and/or your medical records. The only people at sponsor or the Newcastle Clinical Trials Unit who will have access to information that identifies you will be people who need to contact you or audit the data collection process. The people who analyse the information will not be able to identify you and will not be able to find out your name, NHS number or contact details.

The local study team will keep identifiable information about you from this study for 10 years after the study has finished.

When you agree to take part in a research study, the information about your health and care may be provided to researchers running other research studies in this organisation and in other organisations. These organisations may be universities, NHS organisations or companies involved in health and care research in this country or abroad. Your information will only be used by organisations and researchers to conduct research in accordance with the UK Policy Framework for Health and Social Care Research.

This information will not identify you and will not be combined with other information in a way that could identify you. The information will only be used for the purpose of health and care research, and cannot be used to contact you or to affect your care. It will not be used to make decisions about future services available to you, such as insurance.

For peer review only


For peer review only - http://bmjopen.bmj.com/site/about/guidelines.xhtml

## Further information about what will happen to blood samples

- Your samples will be sent securely to the Northern Institute of Cancer Research (NICR) for testing. For selected patients, a sample will also be sent to the All Wales Medical Genetics Laboratory (AWMGL) for testing (to confirm the results of the biomarker test).
- At the start of the study, your blood sample will be tested for the AR-V7 biomarker. Your doctor will receive the result of this test if you are in the group receiving treatment guided by AR-V7 blood test result. If you are in the treatment as usual group neither you nor your doctor will receive the result of the test.
- We may also test your blood sample for the AR-V7 biomarker at the end of the trial. These results will be used to inform the research. Neither you nor your doctor will not receive these results.
- Your blood samples will be stored at the NICR biobank (a collection of samples) and will be used in future research to look at why some patients develop advanced prostate cancer or how the cancer reacts to treatment. The DNA and RNA (the genetic material inside a cell), will be taken out of these stored samples and will also be stored at the NICR biobank. If you do not want your samples to be stored or used through the NICR biobank you will not be able to take part in this trial.
- Although unlikely for the VARIANT trial, there is a small possibility that your blood samples which have been stored in the NICR biobank may be used in research involving rodents (rats or mice). This is only done when it is essential to further our understanding of the way in which a disease develops or responds to treatment. These experiments are performed according to strict guidelines set out by the government and involve minimal stress to the rodents. **If you do not wish for your samples to be used in research using animals you should not complete the box referring to this on the consent form we will ask you to sign.** If you do not consent to your samples being used in research using animals you will still be able to participate in the VARIANT trial.
- If you are selected to provide an additional sample to be sent to AWMGL, the DNA and RNA (the genetic material inside a cell) may be taken out and stored at AWMGL before being transferred to the NICR biobank.
- You and your doctor will not find out the results of any tests done on your stored blood samples. Results of future research with your stored samples will be used to improve care of patients with advanced prostate cancer in the future.
- Your blood samples will be labelled with a unique study number. This number is used to link your sample back to you and only the study team and authorised people will be able to do this. Your sample will be sent with a form that includes your initials, date of birth and the date the sample was taken. This will help us make sure the samples can be linked back to the correct person which is especially important for feeding back the test results.
- Your stored samples may be used by researchers in the UK or overseas (including USA or Europe). Commercial partners may also use your samples for research

For peer review only



For peer review only - http://bmjopen.bmj.com/site/about/guidelines.xhtml

purposes. In most cases the commercial partners will be small companies that were started in universities (university/academic spin-off companies), or drug companies. Working with commercial partners is often important to have the resource to develop tests or products. We will ask partners to sign a legal contract to make sure samples are handled appropriately. Although the research will not be conducted to make money, it is possible that some of the results will be of value to commercial companies, for example in the development of new tests or treatments

- Any money made by sending samples to commercial laboratories will be put in to local research or used to improve patient care. Under UK law, sample donors are not entitled to a share of any profits that may result from this activity.
- The samples will be destroyed after 10 years of the last follow up for the last patient enrolled in the study. This will include all the blood samples and any linked data.

For peer review only


For peer review only - http://bmjopen.bmj.com/site/about/guidelines.xhtml

Consent Form

To be printed on local headed paper

# The Variant 7 Biomarker Feasibility Study

## The **VARIANT** Study

## PATIENT CONSENT FORM

For peer review only

**Site ID number:** ☐☐ **Participant ID Number:** ☐☐☐

**Principal Investigator Name:** ____________

**Please INITIAL these boxed if you agree:**

| | |
|---|---|
| 1. I have read and understood the Patient Information Sheet version _______ dated_____ for the above study. I have had the opportunity to consider the information, ask questions and I am happy with the answers given. | |
| 2. I understand that I do not have to take part in this study. I know that I can withdraw at any time without giving a reason and without my medical care or legal rights being affected. | |
| 3. I understand that information about me will be collected, recorded and used for this study unless I withdraw my consent. I understand that my information will be kept securely and confidentially. | |
| 4. I agree for a copy of my consent form to be sent securely to the Newcastle University Clinical Trials Unit for checking. | |
| 5. I agree to my General Practitioner (GP) being informed about me taking part in this study. | |
| 6. I understand that relevant sections of my medical notes and information collected during the study may be looked at by people from Newcastle Clinical Trials Unit, the study sponsor, regulatory authorities and the local NHS Trust, where it is relevant to my taking part in this research. I give permission for these individuals to have access to my records. | |



For peer review only - http://bmjopen.bmj.com/site/about/guidelines.xhtml

Consent Form

| | |
|---|---|
| 7. I understand that any personal information collected for the study will be kept confidential and not be made public. I understand that anonymised data from the study may be used for other research projects related to prostate cancer and will be published in medical journals, at research meetings and shared with other researchers. | |
| 8. I consent to relevant sections of my medical notes being accessed by members of my local study team to carry out follow-up, including after my participation in the trial has ended, and for there to be a link between my healthcare records and the data collected for the trial. | |

**Sample collection**

| | |
|---|---|
| 9. I consent to the collection of my blood samples as described in the VARIANT patient information sheet and understand that my samples will be sent to the Northern Institute of Cancer research (NICR) and the All Wales Medical Genetic Lab (AWMGL) for testing. | |
| 10. I understand that my samples will not be identifiable except to the trial management team and I give permission for my date of birth and initials to be sent with the blood samples to the lab and with the biomarker result to my study doctor. | |
| 11. I consent to my samples being stored in the NICR biobank for up to 10 years, and give permission for samples to be used as described in the VARIANT Patient Information Sheet. I understand that the biobank will keep my identity confidential and any information collected about me during the study will be anonymised in a way that protects my identity. | |
| 12. OPTIONAL: I consent to my samples to be used in experiments using rodents (rats or mice). | |

**Agree to Participate**

| | |
|---|---|
| 13. I agree to take part in the VARIANT study. | |

____________________ ____________________ ____________________

**Name of patient** **Signature** **Date**

____________________ ____________________ ____________________

**Name of Person** **Signature** **Date**



For peer review only - http://bmjopen.bmj.com/site/about/guidelines.xhtml

**taking consent**

**When completed: 1 copy for participant; 1 copy (original) for Investigator Site File; 1 copy to be kept in medical notes and 1 copy to be sent securely to Newcastle Clinical Trials Unit**

For peer review only



For peer review only - http://bmjopen.bmj.com/site/about/guidelines.xhtml

Completed SPIRIT checklist for VARIANT trial protocol submission:

Administrative Information:

1. Title
2. Trial Registration
3. Protocol Version
4. Funding
5. Roles and Responsibilities

Introduction:

6. Background and Rationale
7. Objectives
8. Trial Design

Methods: Participants, interventions, Outcomes

9. Study Setting
10. Eligibility criteria
11. Interventions
12. Outcomes
13. Participant timeline
14. Sample size
15. Recruitment

Methods: assignment of interventions (for controlled trials)

16. Allocation
17. Blinding (masking)

Methods: data collection, management, analysis

18. Data collection methods
19. Data management
20. Statistical methods

Methods: monitoring

21. Data monitoring
22. Harms
23. Auditing

Ethics and dissemination:

24. Research ethics approval
25. Protocol amendments
26. Consent or assent
27. Confidentiality
28. Declaration of interests
29. Access to data
30. Ancillary and post-trial care
31. Dissemination policy

Appendices

32. Informed consent materials
33. Biological specimens

For peer review only



For peer review only - http://bmjopen.bmj.com/site/about/guidelines.xhtml

For peer review only



For peer review only - http://bmjopen.bmj.com/site/about/guidelines.xhtml
